# Ultrasound-triggered prodrug activation *via* sonochemically induced cleavage of a 3,5-dihydroxybenzyl carbamate scaffold

**DOI:** 10.1039/d5sc05710h

**Published:** 2025-09-30

**Authors:** Xuancheng Fu, Bowen Xu, Hirusha Liyanage, Cijun Zhang, Warren F. Kincaid, Amber L. Ford, Luke G. Westbrook, Seth D. Brown, Tatum DeMarco, James L. Hougland, John M. Franck, Xiaoran Hu

**Affiliations:** a Department of Chemistry, BioInspired Institute, Syracuse University Syracuse New York 13244 USA xhu156@syr.edu; b Department of Biology, Syracuse University Syracuse New York 13244 USA

## Abstract

Spatiotemporal control of drug release in deep tissues is crucial for targeted treatment precision and minimized systemic side effects. Ultrasound is a non-invasive and clinically safe stimulus capable of deep-tissue penetration without requiring optical transparency. Here, we introduce an innovative strategy for controlling cargo release *via* ultrasound-triggered sonochemical cleavage of a 3,5-dihydroxybenzyl carbamate (DHBC) prodrug platform. We demonstrate that low-intensity therapeutic ultrasound (LITUS) effectively generates hydroxyl radicals in aqueous solutions, which hydroxylate DHBC to initiate spontaneous cleavage and cargo release. Using a prototype chemotherapy prodrug (ProDOX) as a proof-of-concept, we show that LITUS irradiation triggers doxorubicin release to kill cancer cells *in vitro*. Remarkably, this sonochemical activation was successfully achieved through 2 cm of chicken breast, highlighting the deep-penetrating capability of our approach. Extending this strategy, we developed ProR848, a sono-activable prodrug of the Toll-like receptors (TLR) agonist R848, enabling remotely triggered, on-demand immune cell activation. Collectively, our results establish a novel and versatile sonochemical cleavage platform for ultrasound-targeted prodrug activation, offering significant potential for applications including controlled therapeutic release and responsive biomaterials.

## Abstract 2

Deep-penetrating low-intensity therapeutic ultrasound (LITUS) induces the cleavage of a 3,5-dihydroxybenzyl carbamate (DHBC) prodrug scaffold *via* a sonochemical activation mechanism, enabling spatiotemporally controlled drug release.

## Graphical abstract

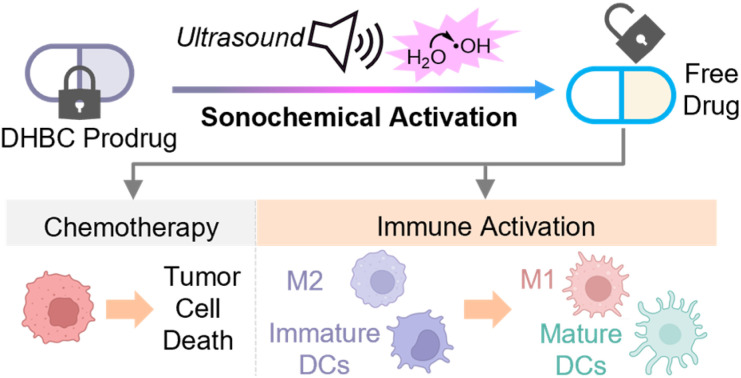

## Introduction

Achieving spatiotemporal control over cleavage chemistries deep within biological tissues is critically important for biomedical applications, such as site-specific drug release and dynamically tunable biomaterials.^1–3^ However, current methods for remotely controlling chemical bond cleavage in deep tissue remain limited. Photo-responsive chemistry has been widely used to control drug release *in vitro* and on skin surfaces, but the limitation of tissue penetration hampers its application in deep tissues.^4,5^ Radiation-controlled drug release has received increasing attention due to its deep-penetrating ability,^6–8^ but it requires specialized equipment, and managing radiotherapy-associated side effects remains a significant concern. Ultrasound (U/S), mechanical sound waves beyond human hearing (20 kHz to MHz range), is widely used in biomedical fields such as deep-tissue imaging and oncology treatment.^9–12^ Ultrasound as a stimulus features a unique combination of advantages: it operates remotely and non-invasively, penetrates deep tissues without needing optical transparency, offers precise targeting, and utilizes cost-effective setups that have been proven safe in clinical applications.

**Fig. 1:**
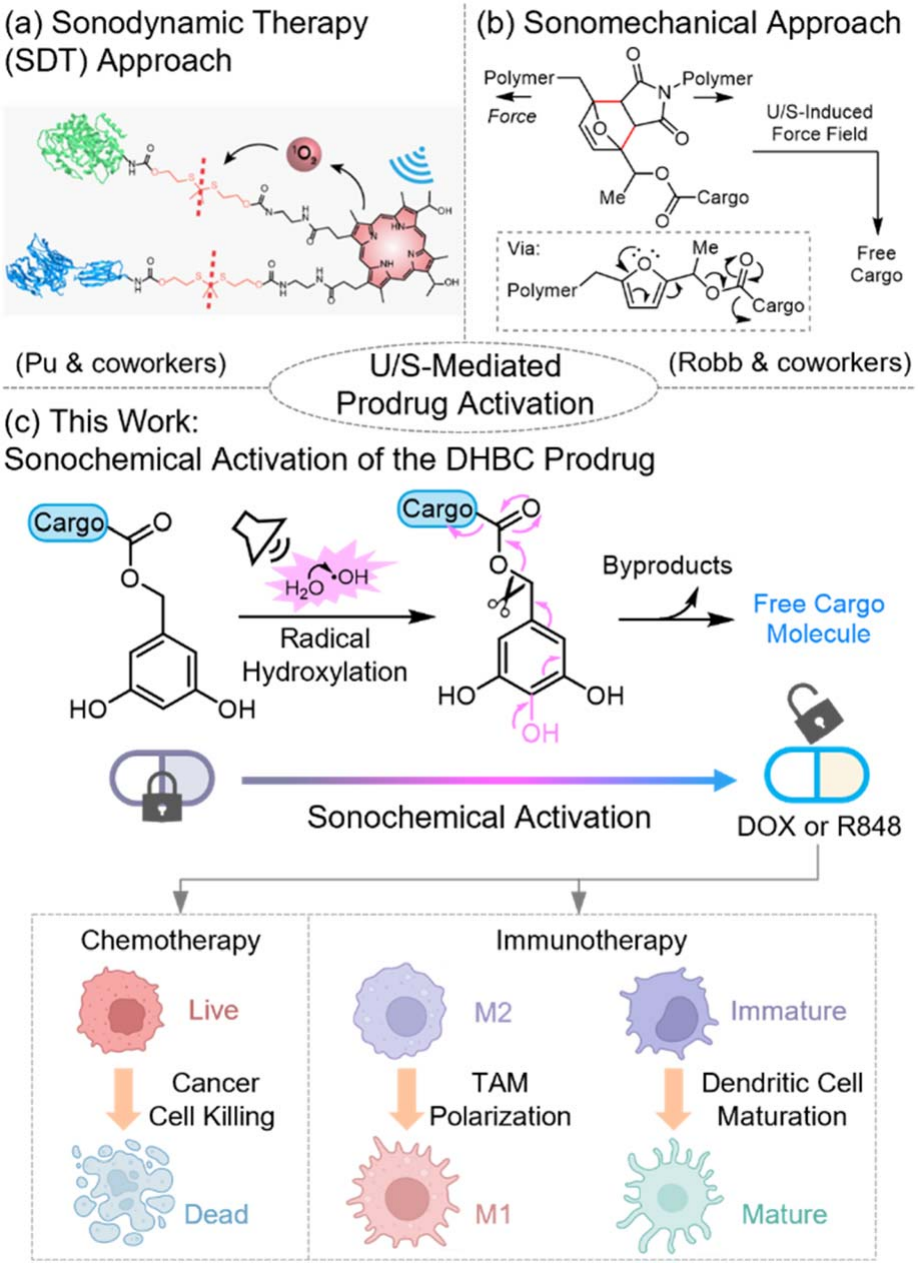
An overview of ultrasound-mediated prodrug activation strategies. This work introduces a sonochemical approach that harnesses the intrinsic chemical effects of ultrasound in aqueous solutions to activate DHBC prodrugs *via* a radical hydroxylation mechanism. Fig. 1a is adapted with permission from ref. 24. Copyright 2022, Springer Nature.

Conventional ultrasound-targeted drug delivery systems harness the physical effects of acoustic waves, such as sonoporation (*i.e.*, ultrasound-induced formation of transient pores in cell membranes, improving membrane permeability) and enhanced extravasation, to improve local pharmacokinetics and drug biodistribution.^13^ However, the utilization of active drugs still poses a risk of off-target side effects. An emerging strategy^14^ addresses this challenge by employing ultrasound-controlled cleavage chemistry (Fig. 1) to activate covalently modified, nontoxic prodrugs exclusively at the target site, enabling localized activation of therapeutic effects while minimizing systemic drug exposure. One such approach (Fig. 1a) utilizes the sonodynamic effect (*i.e.*, ultrasonic generation of reactive oxygen species from sonosensitizers^15–21^) to induce chemical transformations for drug release.^22–26^ However, the requirement for sonosensitizers increases formulation complexity in sonodynamic-based prodrug delivery systems. On the other hand, another ultrasound-mediated bond cleavage strategy (Fig. 1b) leverages the ultrasound-induced shear force field in solution to mechano-chemically activate force-sensitive structures, resulting in bond cleavage and cargo release.^27–33^ Despite recent advancements in the field,^34–36^ conventional polymer-mechanochemistry approaches often involve harsh, high-intensity ultrasonication conditions and necessitate the incorporation of long polymers to prodrug structures (restricting drug loading to <1 wt%), presenting challenges for clinical applicability.

Ultrasound-induced generation of hydroxyl radicals (˙OH) in aqueous environments is a well-established phenomenon in sonochemistry.^37–43^ Clinical acoustic conditions are known to cause acoustic cavitation both *in vivo* and *in vitro*.^44–47^ This cavitation bubble, essentially a vacuum, collapses near-adiabatically and results in extreme pressures over 1000 atm and temperatures above 5000 K, while only slightly affecting the temperature of the bulk liquid. The extreme cavitation environment in collapsing cavitation bubbles serves as sonochemical micro-reactors and is sufficient to cause the pyrolysis of vapor molecules trapped in the bubble, generating primary radicals.^48–51^ For example, Riesz used the methods of spin trapping electron spin resonance (ESR) to directly observe the formation of ˙OH and ˙H in the cavitation bubbles.^42,47,52,53^ These primary radicals can either recombine or diffuse from the gas phase into the vicinity of the bubble and induce a wide variety of secondary chemical reactions in the bulk solution.^54–60^ However, applying these intrinsic chemical effects of ultrasound to drive predictable and constructive chemistry for biomedical applications remains an underexplored yet potentially transformative research venue.^61^

The hydroxyl radical (˙OH), with a Hammett *σ* value of −0.41,^62^ is known to undergo electrophilic substitution reactions, and its ability to hydroxylate aromatic compounds has been studied primarily using ˙OH generated by Fenton's reagent^63,64^ or water radiolysis.^65–67^ Recently, Liu and coworkers elegantly harnessed ˙OH produced from radiolysis to hydroxylate an electron-rich 3,5-dihydroxybenzyl carbamate, triggering cascade chemical transformations that lead to the release of covalently conjugated drugs.^65^ Inspired by ultrasound's intrinsic ability to generate ˙OH radicals and the reactivity of ˙OH in mediating radical hydroxylation,^50,65^ we have developed a sonochemically controlled cleavage platform based on a 3,5-dihydroxybenzyl carbamate (DHBC) prodrug scaffold (Fig. 1c). Using a commercially available, FDA-registered low-intensity therapeutic ultrasound (LITUS) device, sonochemically generated ˙OH radicals react with the DHBC *via* radical hydroxylation, triggering a subsequent elimination cascade that releases the molecular cargo. As a proof-of-concept, we synthesized a model prodrug ProDOX incorporating a chemotherapy drug doxorubicin (DOX), which is selectively activated under LITUS to release DOX and kill cancer cells *in vitro*. To demonstrate the deep-penetration ability of our strategy, we successfully activated ProDOX through a 2 cm thick chicken breast. Further, we extended the platform to immunotherapy by developing ProR848, a sono-activable prodrug of the toll-like receptor (TLR) agonist R848, designed to mitigate the systemic toxicity associated with TLR-based treatments. Upon LITUS irradiation, ProR848 released active R848, selectively activating tumor-associated macrophages (TAMs) and dendritic cells (DCs), as evidenced by upregulation of pro-inflammatory markers and inflammatory cytokine secretion. Together, these chemotherapeutic and immunotherapeutic applications demonstrate the versatility and effectiveness of our deep-penetrating, ultrasound-triggered cleavage platform, offering significant potential for applications ranging from controlled therapeutic release to responsive biomaterials.

## Results and discussion

**Fig. 2:**
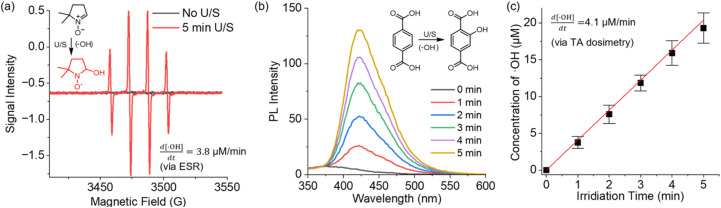
(a) ESR spectra of a 5 mM solution of DMPO in PBS before and after sonication. (b) Sonochemical conversion of TA (20 mM in PBS) to hTA monitored by fluorescence spectroscopy. (c) Concentration of sonochemical ˙OH as a function of sonication time, calculated by multiplying the concentration of hTA by 1/0.35.

We first investigated the sonochemical production of ˙OH radicals using an FDA-registered, commercially available ultrasound device. Under our standard LITUS conditions (frequency: 1 MHz; power: 1.0 W cm^2^; duty cycle: 50%) (see SI for mechanical index calculations and biosafety discussions), the generation of ˙OH in the acoustically irradiated PBS buffer solutions was monitored using ESR with 5,5-dimethyl-1-pyrroline-N-oxide (DMPO), a ˙OH-specific spin trap that forms a well-understood DMPO-OH spin adduct in presence of ˙OH radicals.^53^ LITUS irradiation produced a new set of four-line peaks (Fig. 2a) which are characteristic of the hyperfine coupling in the DMPO–OH adduct, while DMPO–OOH signals were not seen. Comparison with reported hyperfine coupling constants^68^ as well as simulated ESR spectrum (Fig. S1) confirms that these new peaks correspond to the expected DMPO-OH spin adduct. From ESR spin-counting analysis (see SI for details), the concentration of DMPO-OH was determined to be 18.8 μM after 5 min sonication of a 5 mM DMPO solution.

We further performed a quantitative study of ultrasonic ˙OH generation using an established terephthalic acid (TA) dosimetry method—the nonfluorescent TA readily reacts with ˙OH to yield fluorescent 2-hydroxy terephthalic acid (hTA).^69,70^ The fluorescence emission of an irradiated TA solution linearly increased in the first five minutes of ultrasonication (Fig. 2b and c), indicating the steady sonochemical conversion of TA to hTA. It is understood that about 35% of sonochemical ˙OH radicals react with TA to produce hTA,^71,72^ and therefore, the concentration of ˙OH produced in 5 minutes of ultrasonication was calculated to be 20.4 μM (4.1 μM min^−1^), which aligns closely with that estimated by ESR. To confirm the radical nature of the observed hydroxylation of TA, we conducted a control experiment using a highly reactive radical quencher, hydroquinone (rate constant of 11 × 10^9^ M^−1^ s^−1^ with ˙OH).^73^ The addition of 100 mM hydroquinone into a 20 mM TA solution near completely inhibited TA hydroxylation, confirming the key role of radicals (Fig.S5).

**Fig. 3:**
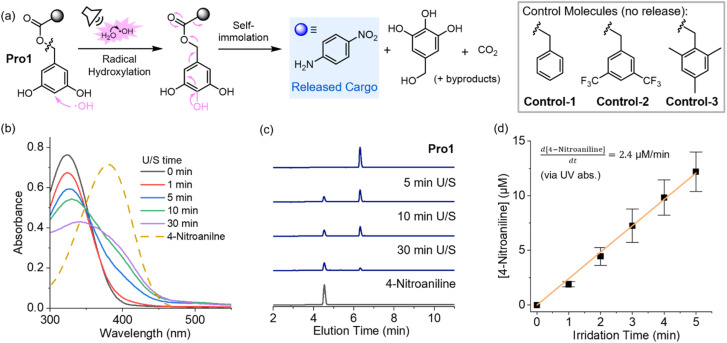
(a) Ultrasonic activation of Pro1 mediated by sonochemical ˙OH radicals. For simplicity, only the hydroxylation reaction at the 4-position is depicted, although hydroxylation at the 2-position is also possible (Fig. S6).^65^ Structures of control molecules are also shown. (b) Absorption spectra of a 50 μM solution of Pro1 in PBS as a function of sonication time. The dashed curve corresponds to the absorbance of a separately prepared 50 μM solution of 4-nitroaniline. (c) Sonolysis of Pro1 monitored by HPLC. (d) The concentration of free 4-nitroaniline in the Pro1 solution in the first 5 minutes of ultrasound irradiation, calculated from the absorbance increase at 400 nm.

Following the sonochemical TA dosimetry experiments, we explored the potential of harnessing sonochemical ˙OH to trigger the radical hydroxylation and cascade molecular release from a DHBC-based model prodrug Pro1. Electron-rich DHBC motifs are designed to react with sonochemically generated ˙OH through a radicalphilic reaction, triggering a cascade elimination process that releases the 4-nitroaniline payload (Fig. 3a).^65^ The release of 4-nitroaniline results in the emergence of a characteristic absorption around 400 nm, providing a convenient signal for monitoring its release using UV-vis spectroscopy. As shown in Fig. 3b, ultrasound irradiation of a 50 μM solution of Pro1 in PBS results in an absorbance increase around 400 nm, corresponding to nitroaniline release. HPLC measurements further confirmed the identity of 4-nitroaniline (Fig. 3c). The rate of 4-nitroaniline release in the first 5 min was estimated at 2.4 μM min^−1^ based on absorbance measurements (Fig. 3d), indicating this model DHBC prodrug was effectively activated under our sonochemical conditions to release the cargo molecules. The release of 4-nitroaniline plateaued at approximately 22 μM after 20 min sonication (Fig. S8). The incomplete conversion is anticipated due to nonspecific sonochemical side reactions—sonochemical degradation of both Pro1 and the released nitroaniline can occur in or near the cavitation microbubbles, which feature extreme environments. Currently, we are unable to identify the sonochemical byproduct(s).

We conducted a series of control experiments to validate the proposed sonolysis mechanism. By introducing 100 mM hydroquinone (radical quencher) into the Pro1 solution, ultrasound-triggered cargo release from Pro1 was inhibited (Fig. S10), supporting that the observed sonochemical activation of Pro1 is through a radical mechanism. Additionally, we designed Control-1/2/3 molecules where the electron-rich 3,5-dihydroxybenzyl motif was replaced: Control-1 and Control-2 contain a less ˙OH-reactive benzyl motif and 3,5-bis(trifluoromethyl)benzyl motif, respectively, while Control-3 comprises a 2,4,6-trimethylbenzyl group, whose hydroxylation product is inactive toward the elimination cascade (Fig. S12). Irradiation of Control-1/2/3 molecules under identical acoustic conditions as used for Pro1 lead to minor increase in 4-nitroaniline absorbance (Fig. S11). HPLC analysis also confirmed the absence of cargo release from sonicated control molecules (Fig. S12).

**Fig. 4:**
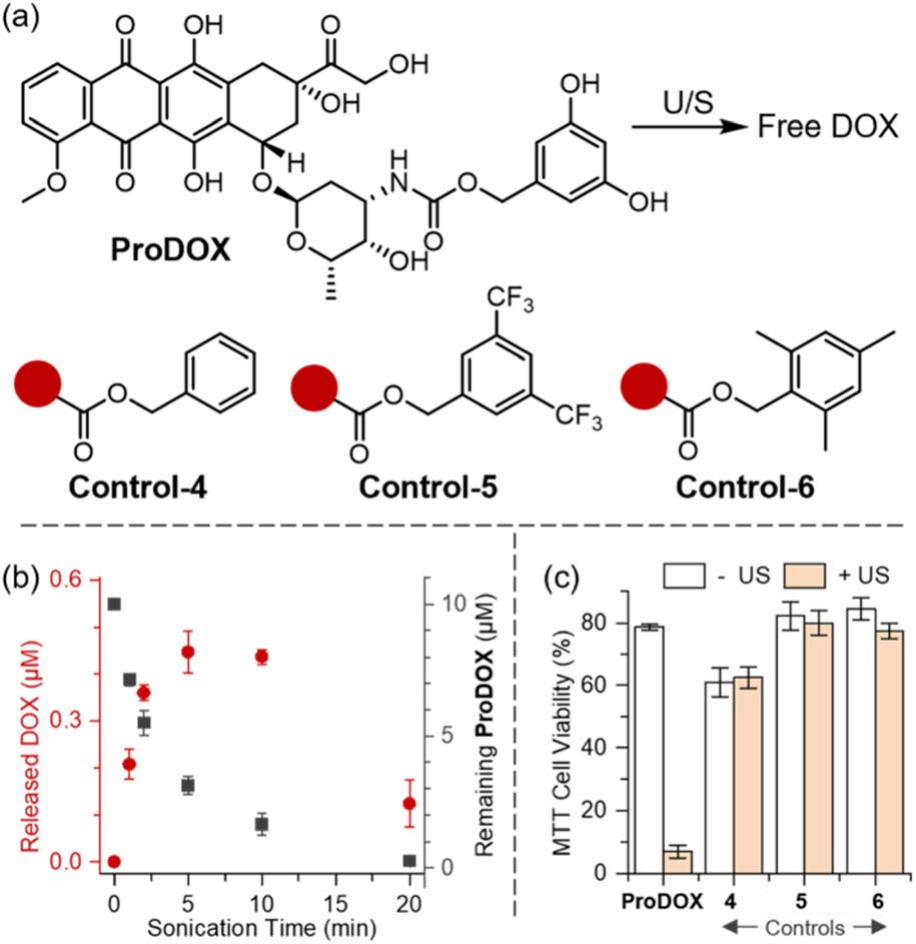
(a) Structures of ProDOX and control prodrugs. (b) Concentration of released DOX from a solution of 10 μM ProDOX in PBS as a function of sonication time. (c) MTT viability assay results demonstrate increased cytotoxicity in LITUS-irradiated ProDOX solution, compared to a nonirradiated ProDOX solution. This ultrasound-induced cytotoxicity is not observed in control prodrugs.

As a proof of concept, we demonstrated ultrasound-triggered release of a cytotoxic chemotherapy drug DOX from the sonochemically responsive DHBC prodrug platform (Fig. 4a). This prototype model prodrug ProDOX was exposed to standard LITUS irradiation, with the reaction monitored by HPLC equipped with a UV detector (monitored at 254 nm). The sonicated solutions displayed a distinct peak at around 4.3 min elution time, corresponding to free DOX released from the activated prodrug (Fig. S14). The DOX peak steadily increased during the first five minutes of ultrasonication, reaching a peak concentration of around 0.5 μM. However, prolonged sonication reduced the DOX concentration, presumably due to the nonspecific sonolysis of DOX under cavitational conditions (Fig. 4b). The appearance of the inflection point for DOX concentration matches the trend observed for 4-nitroaniline release from Pro1 (Fig. S8). Given the electron-rich, anthraquinone structure of DOX, it is particularly susceptible to non-specific degradation under sonochemical conditions (Fig. S15).

While future research will explore the structure-activity relationships affecting the sonochemical stability of therapeutically active structures and will identify candidates with enhanced resistance to sonolysis, the efficacy of our current prototype model prodrug is sufficient to demonstrate the ultrasound-controlled DOX release for *in vitro* cancer treatment. *HeLa* cells were treated *in vitro* by solutions of ProDOX, with or without ultrasonic irradiation (Fig. 4c, left). Only the sonicated ProDOX (yellow bar) exhibited significant cytotoxicity, confirming that ultrasonic irradiation activated the cytotoxicity of ProDOX. Meanwhile, control groups with DOX masked by various benzyl derivatives showed limited toxicity both in the presence and in the absence of sonication, with HPLC confirming no DOX release (Fig. S14).

**Fig. 5:**
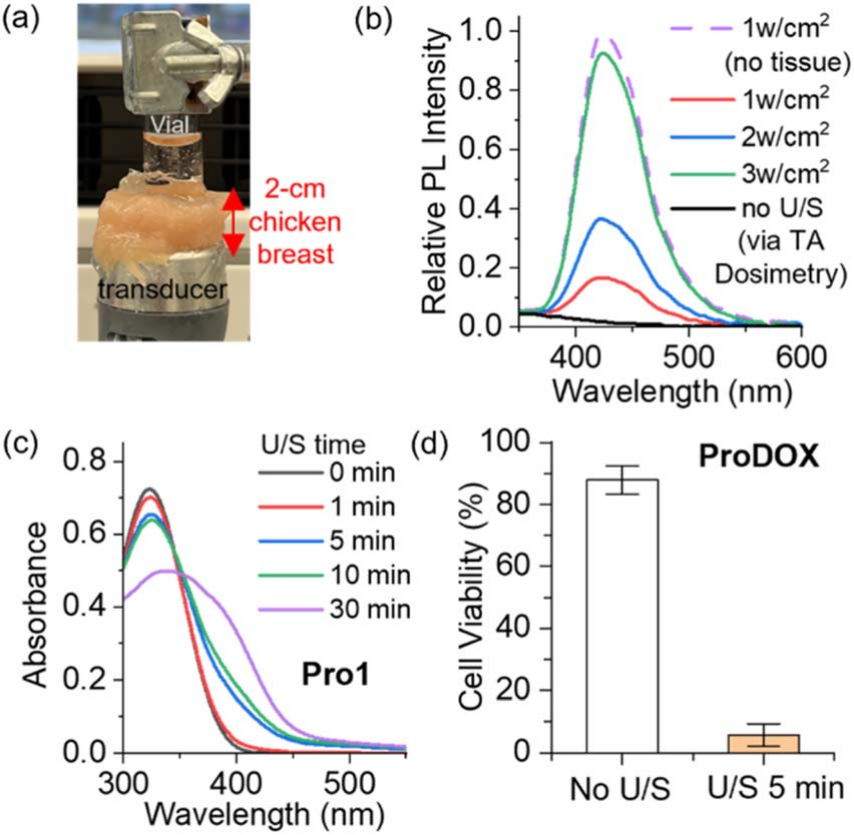
(a) A photograph showing our setup applying LITUS through a 2 cm thick chicken breast tissue to a solution. (b) Fluorescence spectra of 20 mM TA solutions after 5 min of LITUS irradiation at varied sound intensity (1 MHz, 50% duty cycle) applied through chicken breast. All traces are normalized relative to the fluorescence of a TA solution sonicated (1 Wcm^−2^, 5 min) without chicken breast (dashed line). (c) Absorption spectra monitoring the release of 4-nitroaniline from a 50 μM Pro1 solution as a function of sonication (3 W cm^−2^, through chicken breast). (d) MTT viability assay with *HeLa* cells show LITUS-induced (3 W cm^−2^, through chicken breast) cytotoxicity of ProDOX.

Then, we demonstrate the tissue-penetration ability of our controlled-release techniques by remotely manipulating the chemical transformation of prodrugs using LITUS through a 2 cm-thick chicken breast (Fig. 5a). Through the animal tissue, our standard 1 W cm^−2^ LITUS condition successfully triggered the hydroxylation of TA as indicated by fluorescence turn-on, while moderately increased acoustic intensity at 3 W cm^−2^ exhibits more pronounced sonochemical effects (Fig. 5b)–this 3 W cm^−2^ intensity was used in all tissue-penetrating experiments. Ultrasound irradiation applied through the chicken breast successfully triggered the release of 4-nitroaniline from Pro1 (Fig. 5c) as well as DOX from ProDOX (Fig. S18). Ultrasound applied through chicken breast effectively activated ProDOX solutions, enhancing their cytotoxicity against *HeLa* cells *in vitro* (Fig. 5d).

**Fig. 6:**
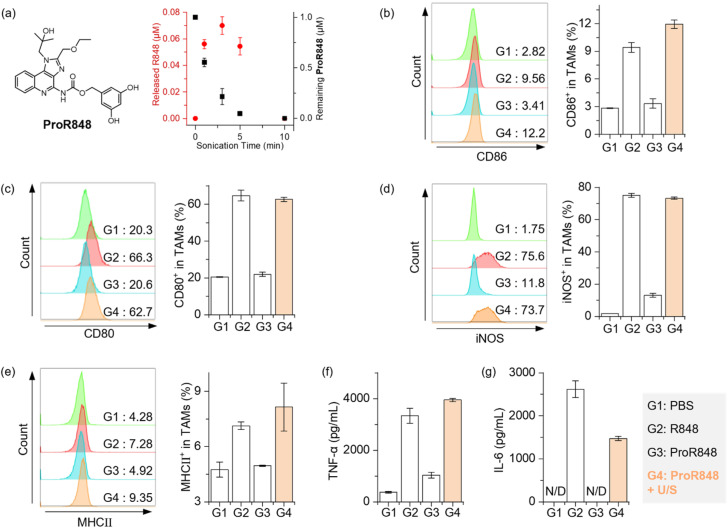
(a) Structures of ProR848 and concentration of released R848 from a solution of 1 μM ProR848 in PBS as a function of sonication time (1 MHz, 50% duty cycle, 1 W cm^−2^), quantified by HPLC. (b–e) Flow cytometry analysis showing enhanced expression of pro-inflammatory markers on tumor-associated macrophages (TAMs) after treatment with ProR848 activated by 3 min ultrasound irradiation (G4, orange bars), compared with PBS negative control (G1), free R848 positive control (0.07 μM, G2), and non-sonicated ProR848 (G3): (b) CD86, (c) CD80, (d) iNOS, (e) MHC class II. (f and g) ELISA measurements demonstrating secretion of pro-inflammatory cytokines from TAM supernatants after various treatments (G1 to G4): (f) TNF-α, (g) IL-6.

TLR agonists represent potent immunotherapeutic agents capable of enhancing immune activation and remodeling immunosuppressive tumor microenvironments.^74,75^ This effect is primarily mediated through the activation of immune cells, particularly by polarizing tumor-associated macrophages (TAMs) from an anti-inflammatory, pro-tumoral M2-like phenotype to a pro-inflammatory, anti-tumoral M1-like phenotype.^76–78^ However, systemic administration of TLR agonists is limited clinically by severe side effects, notably cytokine storm.^79^ Therefore, strategies enabling targeted release of TLR agonists have shown great potential to confine immune activation to the tumor site and reduce systemic toxicity.^80–85^ Herein, we leverage our sono-responsive DHBC platform to precisely control the release of the TLR agonist (R848) under LITUS. Our pro-agonist, ProR848, demonstrates outstanding biocompatibility towards TAMs, exhibiting negligible toxicity at 10 μM (Fig. S19a). Evaluation of inflammatory markers CD86 and CD80 revealed that TAMs activation by 1 μM ProR848 was minimal (Fig. S19b and c), indicating its potential for minimizing systemic immune activation. Subsequent LITUS-mediated activation of 1 μM ProR848 was monitored using HPLC (Fig. 6a). Ultrasonicated samples exhibited a distinct chromatographic peak at approximately 6.6 min (Fig. S20), indicative of the release of active R848 cargos. R848 release peaked at approximately 0.07 μM within the first three minutes of sonication and subsequently decreased upon prolonged irradiation (Fig. 6a).

Having confirmed LITUS-triggered R848 release, we evaluated its ability to induce TAMs polarization. PBS-treated and 0.07 μM R848-treated groups served as negative and positive controls, respectively. Flow cytometric analysis demonstrated that ultrasound-activated ProR848 (G4, orange bars) significantly upregulated pro-inflammatory markers CD86, CD80, and inducible nitric oxide synthase (iNOS) in TAMs, mirroring the response elicited by free R848 (G2) treatment (Fig. 6b–d). In contrast, TAMs exposed to non-sonicated ProR848 (G3) exhibited marker expression comparable to PBS controls (G1), demonstrating that the TAMs polarization was due to LITUS-mediated drug release. Additionally, a substantial enhancement in major histocompatibility complex class II (MHCII) expression endowed macrophages with augmented antigen-presenting capabilities, facilitating improved activation and maturation of CD4^+^ helper T cells and subsequent adaptive immune responses (Fig. 6e).^86,87^ The immunostimulatory efficacy of ProR848 under LITUS irradiation was further corroborated by enzyme-linked immunosorbent assay (ELISA) data, revealing significantly elevated secretion of proinflammatory cytokines tumor necrosis factor-alpha (TNF-α) and interleukin-6 (IL-6) following ultrasound treatment (Fig. 6f and g).

Dendritic cells (DCs) are another immune cell type with important roles in orchestrating innate and adaptive immunity. We further evaluated the effects of ultrasound-activated ProR848 on DC maturation using the DC2.4 cell line. Similar to TAMs, DC2.4 cells exhibited excellent tolerance to 1 μM ProR848, without evidence of DCs maturation (Fig. S21a and b). Remarkably, upon ultrasound exposure, significant maturation of DC2.4 cells was observed, as evidenced by pronounced increases in MHCII expression (Fig. S21c). Collectively, our results demonstrate that LITUS-triggered R848 release from ProR848 effectively activates TAMs and promotes DCs maturation. This strategy enables on-demand and localized immune cell activation and holds promise for targeted immunotherapy with reduced systemic immune-related adverse effects.

## Conclusions

This work introduces a sonochemical strategy to control prodrug activation through ultrasound-triggered cleavage of a DHBC prodrug platform. Using a commercially available, FDA-registered therapeutic ultrasound device, we demonstrated that our standard LITUS conditions generate ˙OH radicals at a rate of several μM min^−1^. The DHBC prodrug scaffold is designed to undergo radical hydroxylation by these sonochemically generated ˙OH radicals, triggering a self-immolative cascade to release the cargo molecule. Using a chemotherapy prodrug model ProDOX as a proof-of-concept, we show that LITUS irradiation triggers DOX release, effectively killing *HeLa* cells *in vitro*. Notably, sonochemical manipulation of the DHBC prodrugs was successfully achieved through a layer of chicken breast, highlighting the deep-penetration capability of our approach. Moreover, to address systemic toxicity associated with TLR agonists in immunotherapy, we developed a LITUS-activable prodrug ProR848. Upon LITUS activation, ProR848 released R848 and induced the polarization of TAMs and maturation of DC cells, demonstrating the potential to trigger localized immunostimulatory activity through our sonochemical strategy. Together, these results demonstrate the versatility of our sonochemical cleavage platform for controlled release of chemotherapy and immunomodulatory drugs, offering potential for targeted delivery in deep tissues inaccessible by conventional noninvasive stimuli. Future work will focus on understanding structure-sonochemical reactivity relationships in bioactive substances and designing prodrug molecules with enhanced resistance to unspecific sonolysis.

## Ethical statements

All experimental procedures, including chemical synthesis, analytical measurements, cell culture studies, and experiments with chicken breast tissue were conducted in compliance with the relevant national regulations and institutional guidelines at Syracuse University (approval committees include the Environmental Health and Safety Services, the Institutional Biosafety Committee, and the Institutional Animal Care and Use Committee).

## Author contributions

X. Fu led the study and contributed to the manuscript writing. B. Xu, H. Liyanage, C. Zhang, W. F. Kincaid, A. L. Ford, L. G. Westbrook, S. D. Brown, and T. DeMarco contributed to the experimental work. J. L. Hougland and J. M. Franck supervised students and provided research resources. X. Hu conceived and oversaw the project, secured funding and resources, and contributed to the manuscript writing.

## Conflicts of interest

There are no conflicts to declare.

## Supplementary Material

SC-OLF-D5SC05710H-s001

## Data Availability

Supplementary information: experimental details, supporting figures, synthetic procedures, UV-vis, fluorescence, HPLC, and NMR spectra. See DOI: https://doi.org/10.1039/d5sc05710h.
