# Supplementary material for: Ultrasound-triggered prodrug activation via sonochemically induced cleavage of a 3,5-dihydroxybenzyl carbamate scaffold
Source: Chem Sci. 2025 Sep 30;16(44):21000–9. doi: 10.1039/d5sc05710h (PMC12516518; doi:10.1039/d5sc05710h)
Supplement: SC-016-D5SC05710H-s001 [file SC-016-D5SC05710H-s001.pdf]

Supplementary Information (SI) for Chemical Science.
This journal is © The Royal Society of Chemistry 2025

Supporting Information

Ultrasound-Triggered Prodrug Activation via Sonochemically Induced Cleavage of a 3,5-Dihydroxybenzyl Carbamate Scaffold

Xuancheng Fu,[a] Bowen Xu,[a] Hirusha Liyanage,[a] Cijun Zhang,[a] Warren F. Kincaid,[a] Amber L. Ford,[a] Luke G.Westbrook,[a] Seth D. Brown,[a] Tatum DeMarco,[a] James L. Hougland,[a] [b] John M. Franck,[a] Xiaoran Hu*[a]

[a] Department of Chemistry, BioInspired Institute, Syracuse University, Syracuse, New York, 13244, United States
[b] Department of Biology, BioInspired Institute, Syracuse University, Syracuse, New York, 13244, United States

*To whom all correspondence should be addressed: Prof. Dr. Xiaoran Hu, xhu156@syr.edu

Contents

1. General Considerations 2
2. Supplementary Figures 4
3. MTT Experiments 17
4. Immune Cell Activation Experiments 17
5. Synthetic Details 18
5. NMR Spectra 28

1. General Considerations

All reactions were conducted under standard air-free conditions under an atmosphere of nitrogen gas with magnetic stirring unless otherwise mentioned. All reactants and solvents were purchased from commercial suppliers and used without further purification unless otherwise noted. Flash chromatography was performed on a Biotage Isolera System with Yamazen Corp. universal silica gel columns (Pore Size 60 angstroms, Particle Size 40-63 microns). APC anti-mouse CD86 Antibody, APC anti-mouse CD80 Antibody, PE anti-Nos2 (iNOS) Antibody, APC anti-mouse I-A/I-E Antibody, ELISA MAX™ Deluxe Set Mouse TNF-α, and ELISA MAX™ Deluxe Set Mouse IL-6 were purchased from Biolegend and were used according to the manufacturers' instructions.

NMR spectra were acquired on a Bruker Avance III HD 400 MHz spectrometer. ^{1}H NMR spectra are reported relative to residual protonated solvent (7.26 ppm for $CDCl_3$, 2.50 ppm for DMSO-d_6, 3.31 for Methanol-d_4). ^{13}C NMR spectra are reported relative to residual protonated solvent (77.16 ppm for $CDCl_3$, 39.52ppm for DMSO-d_6, 49.00 for Methanol-d_4). Multiplicity abbreviations are as follows: s = singlet, d = doublet, t = triplet, q = quartet, dd = doublet of doublets, ABq = AB quartet, m = multiplet, br = broad.

Mass spectra were acquired on a DART-SVP (Direct Analysis in Real Time) ion source (IonSense, Saugus, MA) coupled to an Exactive Orbitrap mass spectrometer (Thermo Scientific, Bremen, Germany) at the Cornell Chemistry Mass Spectrometry Facility.

High-Performance Liquid Chromatography (HPLC) measurements were performed with Shimadzu LC-20 HPLC system equipped with Shimazu Nexcol C18 5 µm column with a flow rate of 1 mL/min.

All solution optical spectra were acquired of samples in quartz cuvettes. Electronic absorbance spectra were acquired with an Evolution 201 UV-visible spectrophotometer in double-beam mode using a solvent-containing cuvette for background subtraction spectra of solution samples. Fluorescence spectra were measured with an Agilent Cary Eclipse G9800A Fluorescence Spectrophotometer.

Ultrasonication experiments were performed using an **Intelect Transport Ultrasound system** equipped with a 3 cm Intelect ultrasound transducer. Unless otherwise mentioned, all sonication experiments were performed using our standard ultrasound parameters: frequency = 1 MHz, power = 1.0 W/cm^2, duty cycle = 50%. In a typical experiment, a 2 mL solution was added to a 4 mL glass vial and placed directly on the transducer. To enhance ultrasonic transmission efficiency, Aquasonic 100 Ultrasound Transmission Gel was applied between the glass vial and the transducer. The temperature of ultrasonically irradiated solutions is controlled between 15-18 °C during the course of ultrasonication.

Safety Aspects of Ultrasound: The acoustic peak pressure P can be calculated as $P = \sqrt{2 * \rho * c * I}$, where ρ is the medium density, c is the sound speed in the medium, I is the acoustic

intensity. Higher acoustic intensity leads to stronger cavitational effects and compromised safety. The **mechanical index (*MI*)**, calculated as $MI = P * \sqrt{f}$ where f is the U/S frequency, is used to assess the risk of cavitation in tissues. Higher MI values indicate a greater likelihood of mechanical damage. The FDA recommends a safety limit of MI = 1.9 for diagnostic U/S. Note that **the MI value of our standard LITU setup (frequency = 1 MHz, power = 1.0 W/cm², duty cycle = 50%) is theoretically estimated to be approximately 0.2** using the equations described above, well below the FDA limit.

A stock solution of terephthalic acid (TA) was prepared with the following composition (Ref: *Ultrason. Sonochem.* **1994**, *1*(2), S91–S95.): 0.332 g TA (2.0×10^{-3} mol), 0.200 g NaOH (5.0×10^{-3} mol), and a phosphate buffer (pH 7.4) made up from 0.589 g KH_2PO_4 (4.4×10^{-3} mol) and 0.981 g Na_2HPO_4 (7.0×10^{-3} mol). This solution was then made up to 100 mL with water, giving a TA concentration of 20.0×10^{-3} mol/L. This solution was used directly for ultrasound experiments without further dilution.

Preparation of **Pro1** ,**Control-1**, **Control-2**, and **Control-3** for ultrasound experiments: Stock solutions of **Pro1**, **Control-1**, **Control-2**, and **Control-3** were prepared at a concentration of 100 mM in anhydrous DMF. These stock solutions were then diluted in 10 mM pH 7.4 PBS to obtain a final concentration of 50 μM for ultrasound experiments.

Preparation of **ProDOX**, **Control-4**, **Control-5**, **Control-6**, and **ProR848** for ultrasound experiments: Stock solutions of **ProDOX**, **Control-4**, **Control-5**, **Control-6**, and **ProR848** were prepared at a concentration of 10 mM in anhydrous DMF. These stock solutions were then diluted in 10 mM pH 7.4 PBS to achieve a final concentration of 10 μM for ultrasound experiments.

ESR Assay and Determination ·OH Concentration from ESR Spectra: Electron spin resonance (ESR) spectroscopy can detect unpaired electrons by observing their coupling to a magnetic field. The measurements detailed here were acquired on a Bruker E500 cw EPR spectrometer with a SuperX Bridge and ELEXSYS Super High Sensitivity Probehead, with a conversion time of 12 ms, 3413 points over a field sweep width of 150 G (15 mT), at a microwave power of 2.0 mW and frequency of 9.835 GHz. Samples were positioned inside a capillary with inner diameter 0.7 mm, and length of greater than 41 mm. As for all cw ESR spectra, the y-axis of the spectrum corresponds to the derivative of the absorption. In these spectra, hyperfine interactions perturb the main Zeeman interaction between the spins and the magnetic field in order to generate splittings. These hyperfine splittings depend on the nature of the nuclei accessible to the unpaired electron spin. The concentration of the generated radicals was determined with the Quantitative EPR tool in the Bruker Xepr software. From the first derivative spectra, Xepr allows the user to visually select the region for double integration, as well as a region on either side of the signal that defines the baseline. Xepr's spin count program accepts the sample's expected spin quantum number (here 1/2) sample diameter and positioning to determine the number of detected spins and thereby the sample's concentration.

2. Supplementary Figures

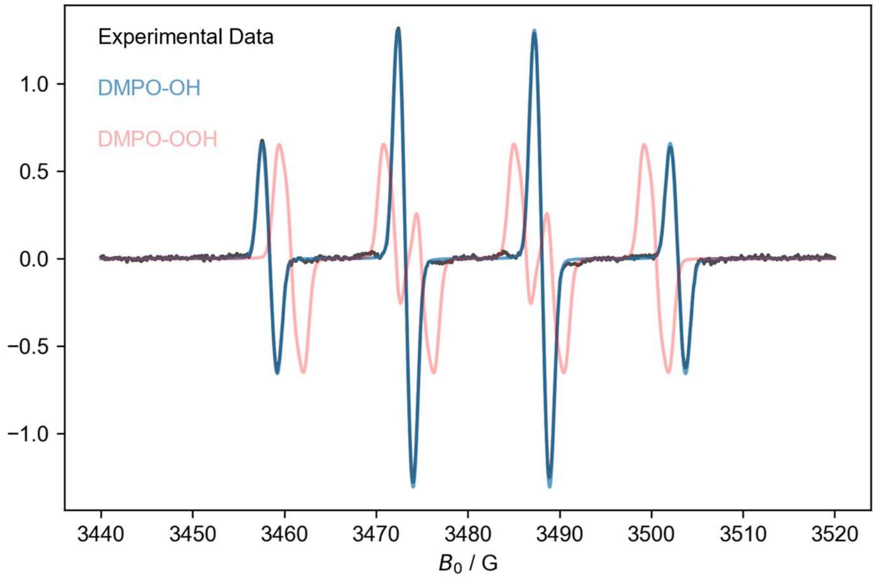

Figure S1. Experimental ESR spectrum (black), simulated spectrum of DMPO–OH (blue), and simulated spectrum of DMPO–OOH (pink). The observed four-line peaks match the expected DMPO–OH adduct, while DMPO–OOH signals were not detected experimentally.

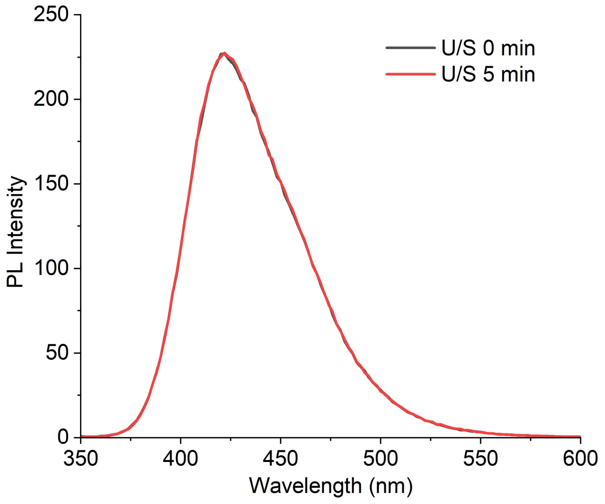

Figure S2. Fluorescence spectra of 10 μM 2-hydroxyterephthalic acid (hTA) in 10 mM pH 7.4 PBS before and after 5 min of ultrasound exposure. The lack of spectral changes suggests that hTA remains stable during the first 5 minutes of ultrasonication, ensuring the reliability of hTA generation as a quantitative measure of ultrasound-induced hydroxyl radical production.

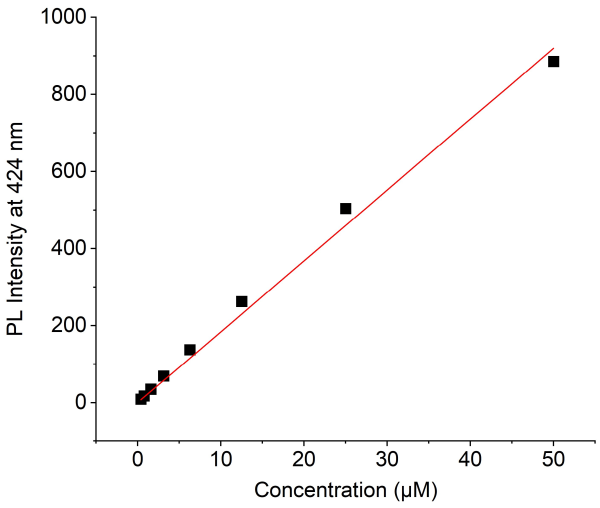

Figure S3. Construction of a calibration curve for experimental determination of the concentration of hTA, plotting the PL (photoluminescence) intensity at 424 nm for hTA solutions in PBS against concentration. A linear regression of the data in plot gives the calibration function, Y= 18.399*X

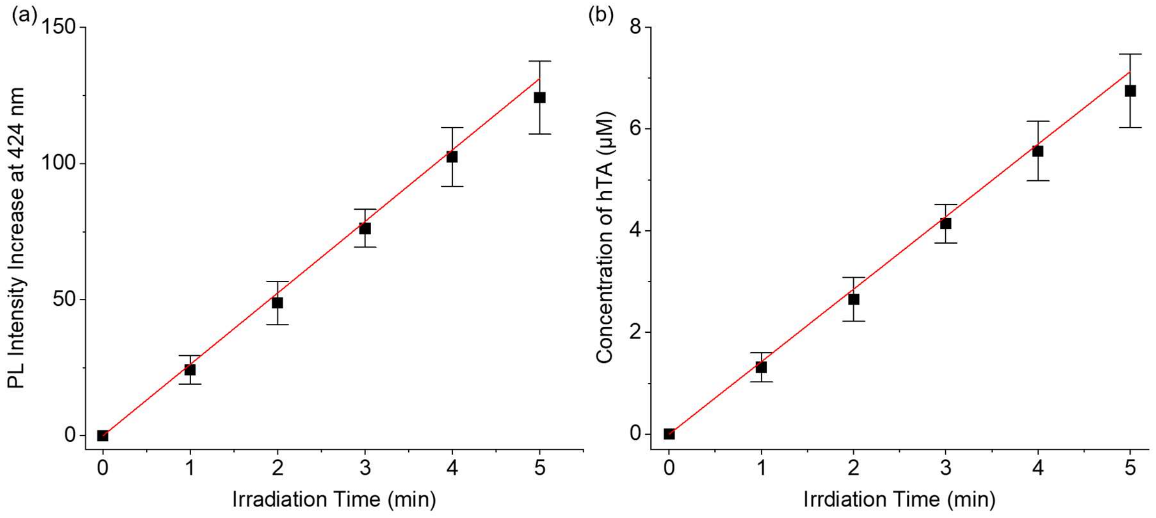

Figure S4. (a) PL intensity at 424 nm of a 20 mM TA solution as a function of ultrasound exposure time. The increase of PL signal at 424 nm indicates the hydroxylation of TA from ultrasound-induced hydroxyl radical generation. (b) Corresponding the concentration of generated hTA over ultrasound time. Data represents the mean of three independent trials.

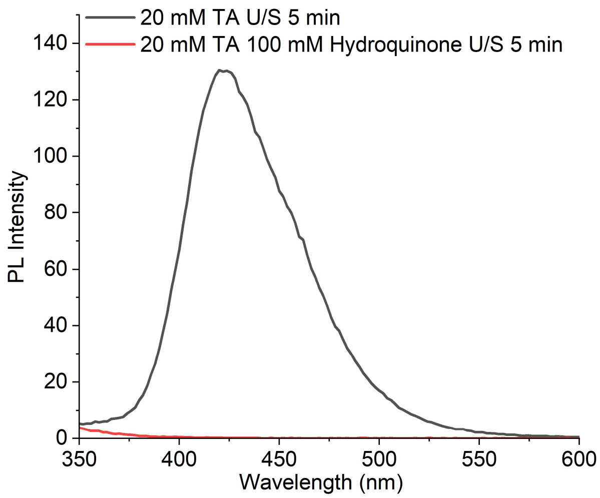

Figure S5. 5 min of ultrasonic irradiation of a 20 mM TA solution turned on its fluorescence (black), while identical irradiation of a 20 mM TA solution containing 100 mM hydroquinone as a radical quencher caused minor change in its emission.

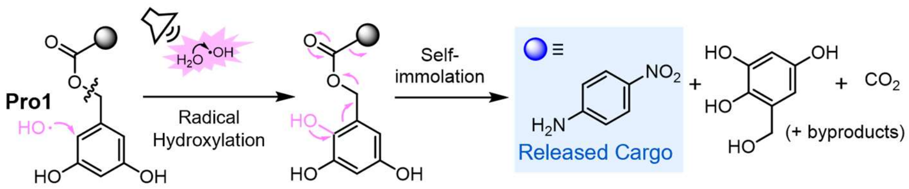

Figure S6. Ultrasonic activation of **Pro1** at the 2-position mediated by sonochemical ·OH radicals. Both hydroxylation products at the 2- and 4-positions of DHBC were confirmed in Liu's study under radiolysis conditions (Ref 65 in the manuscript).

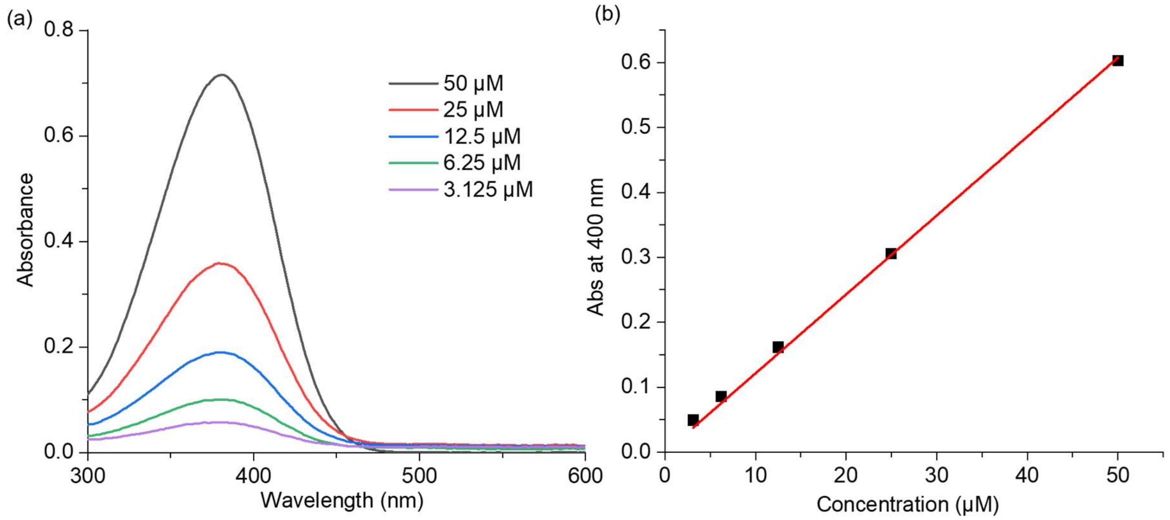

Figure S7. Construction of a calibration curve for experimental determination of the concentration of 4-nitroaniline. (a) Absorbance spectra and (b) intensity at 400 nm for solutions of 4-nitroaniline in PBS as a function of concentration. A linear regression of the data in (b) gives the calibration function, Y= 0.01215*X.

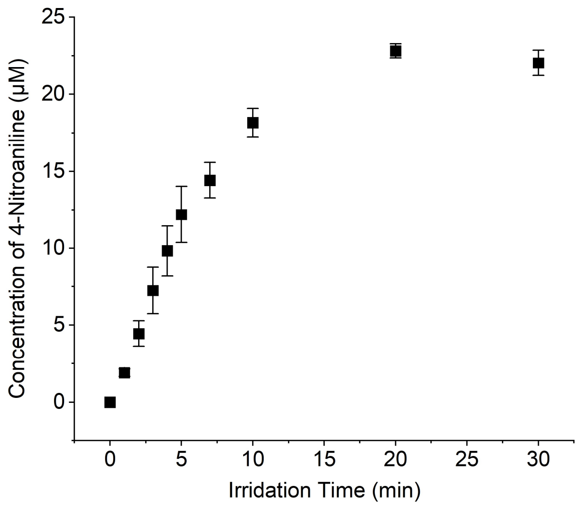

Figure S8. Concentration of released 4-nitroaniline from a solution of 50 μM **Pro1** in PBS as a function of sonication time. The maximum released concentration from **Pro1** was determined to be 22 μM. The incomplete conversion may be due to the pyrolysis of **Pro1** and 4-nitroaniline caused by the extreme conditions within cavitation microbubbles, where temperature can reach ~5000°C and pressure ~1000 atm. Data represent the mean of three independent trials.

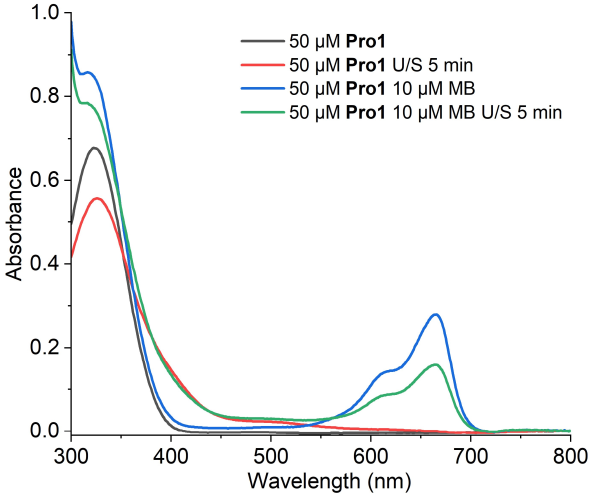

Figure S9. Absorbance spectra of 50 μM **Pro1** with or without 10 μM sonosensitizer methylene blue (MB) before and after 5 min of ultrasound exposure. The comparable increase at 400 nm indicates that the presence of MB does not enhance 4-nitroaniline generation. This is because sonosensitizer primarily produces singlet oxygen under ultrasound, rather than ·OH radicals. In contrast, the release of nitroaniline from **Pro1** is specifically triggered by ·OH generated during sonication.

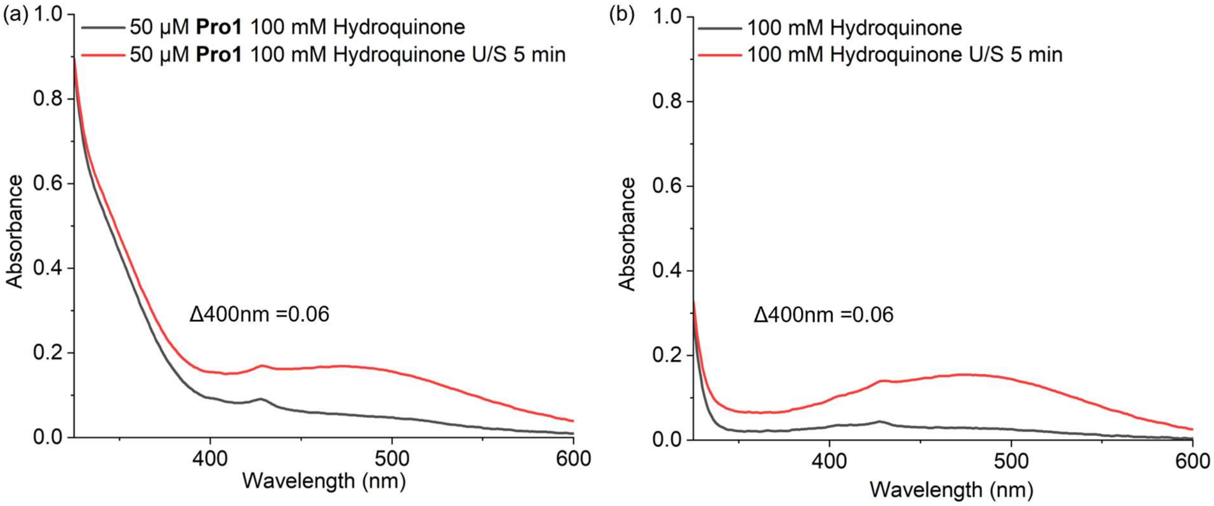

Figure S10. Absorbance spectra of 50 μM **Pro1** with 100 mM hydroquinone (a) and 100 mM hydroquinone alone (b) before and after 5 min of ultrasound exposure. The absorbance increase at 400 nm was measured as 0.06 in both the **Pro1** with hydroquinone group and the hydroquinone-alone group. This confirms that the increase is due to the oxidation of hydroquinone rather than the release of 4-nitroaniline from **Pro1**. The absence of 4-nitroaniline release from **Pro1** when ·OH was quenched further supports the mechanism of hydroxyl radical-induced 4-nitroaniline release.

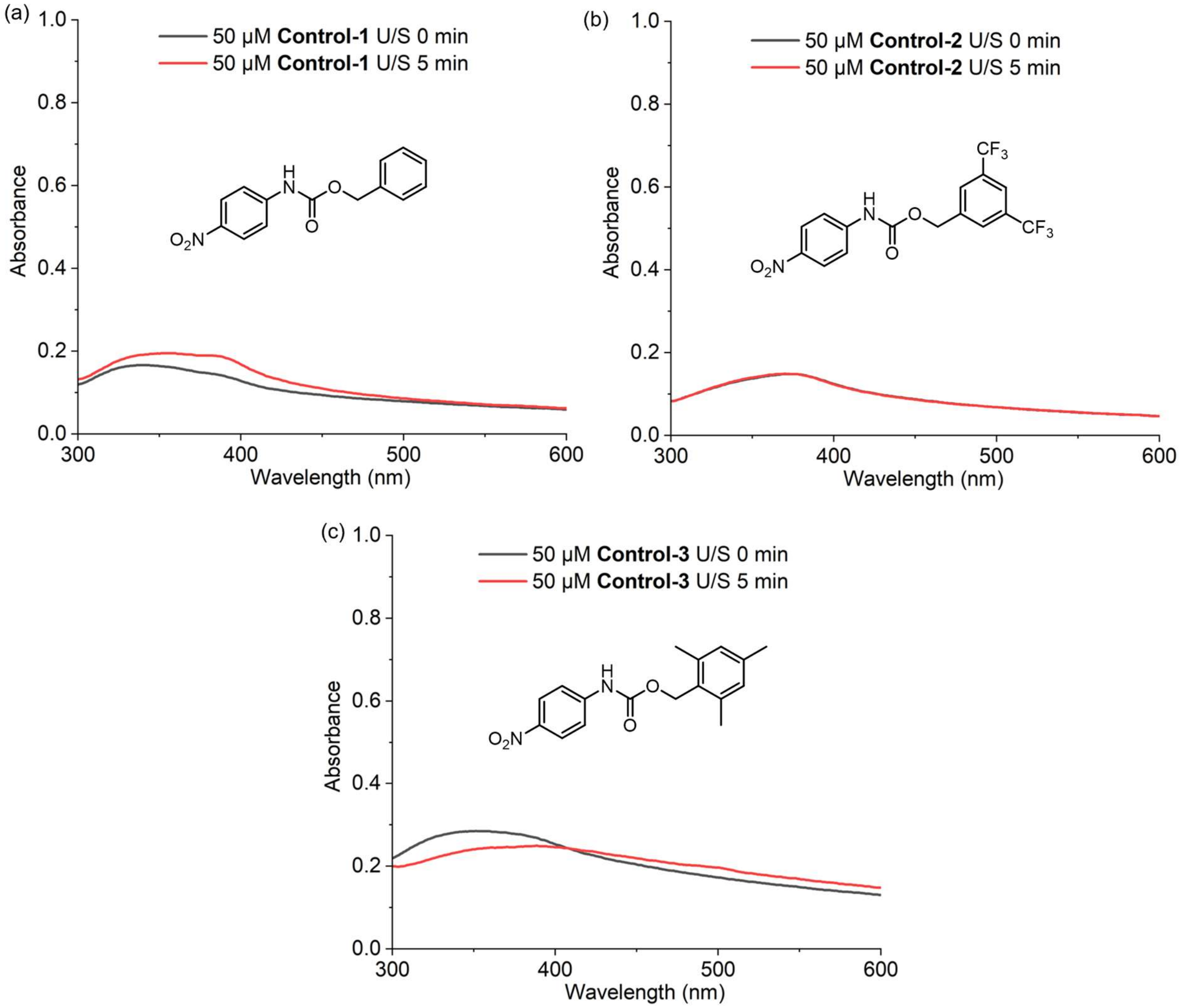

Figure S11. Absorbance spectra of control molecules (a: **Control-1**, b: **Control-2**, c: **Control-3**) before and after ultrasound treatment. Minor change in absorbance around 400 nm was observed after ultrasound exposure, indicating negligible 4-nitroaniline release from these control molecules. This result supports the reactivity of the 3,5-dihydroxybenzyl unit toward electrophilic hydroxyl radicals.

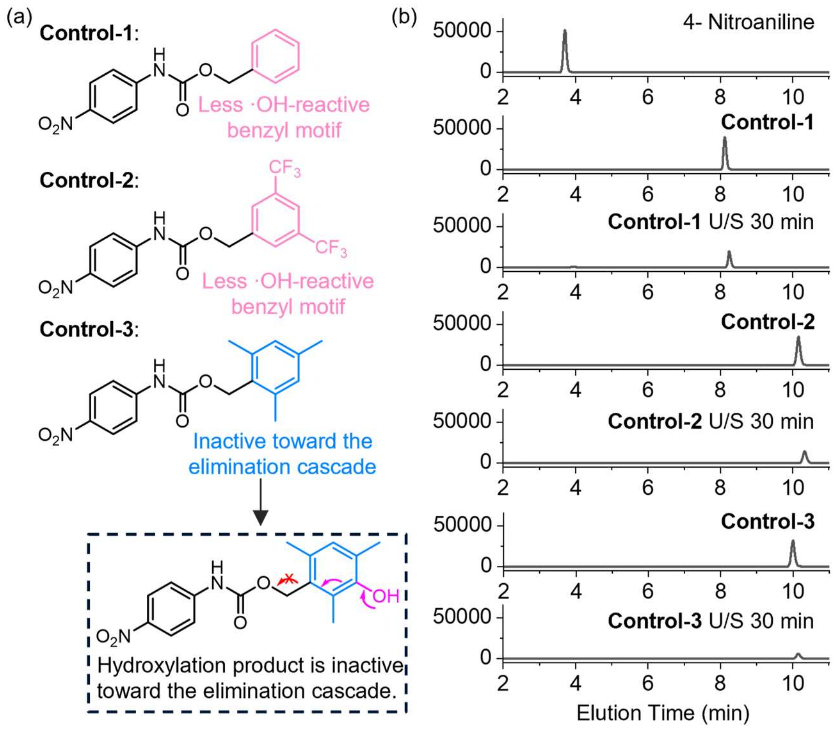

Figure S12. (a) Structures and rational design of **Control-1/2/3**. (b) Ultrasonic activation (30 min standard LITUS) of **Control-1/2/3** monitored by HPLC equipped with a UV-vis detector (λ = 350 nm). 4-Nitroaniline release are absent in control groups, which is consistent with the absorbance data in Figure S11, further validating the importance of the reactive 3,5-dihydroxybenzyl unit in our design. The concentration of all analyte was 50 μM. HPLC conditions: MeCN/water gradient (10:90 to 65:35 over 11 min), C18 column, 25°C, flow rate = 1 mL/min.

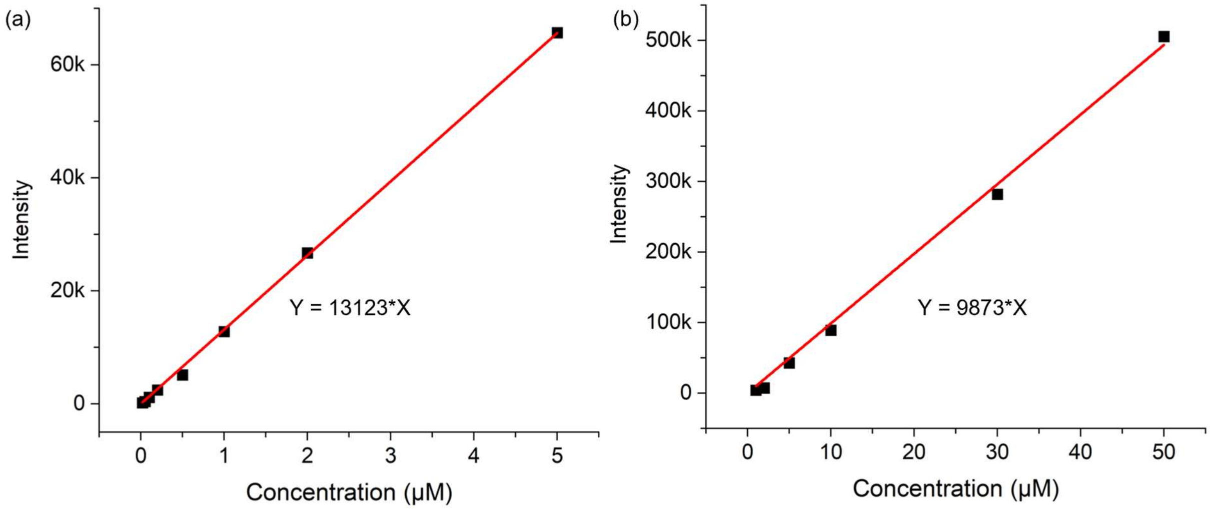

Figure S13. Construction of a calibration curve for the experimental determination of the concentration of DOX (a) and **ProDOX** (b) using HPLC.

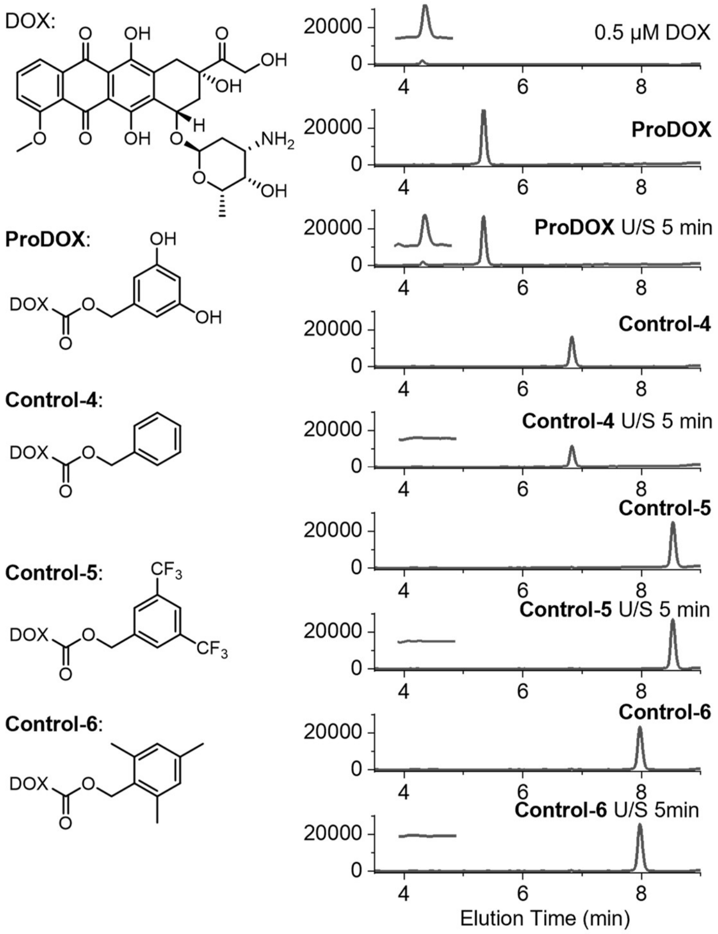

Figure S14. Ultrasonic activation (5 min standard LITUS) of **ProDOX** and corresponding control molecules monitored by HPLC equipped with a UV-vis detector (λ = 254 nm). Each plot for the irradiated groups include an inset showing the enlarged region around 4.2 min elution time. DOX release is only observed for the **ProDOX** group, but are absent in control groups. This result is consistent with findings from Figure 3c and Figure S12. The concentration of **ProDOX**, **Control-4**, **Control-5**, and **Control-6** was 10 µM for ultrasound treatment and analyzed directly by HPLC without further dilution. The concentration of DOX released from 5 min sonicated **ProDOX** solutions was determined to be 0.5 µM, which is sufficient to inhibit the growth of HeLa cells. HPLC conditions: MeCN/water gradient (10:90 to 65:35 over 11 min), C18 column (25°C), flow rate of 1 mL/min.

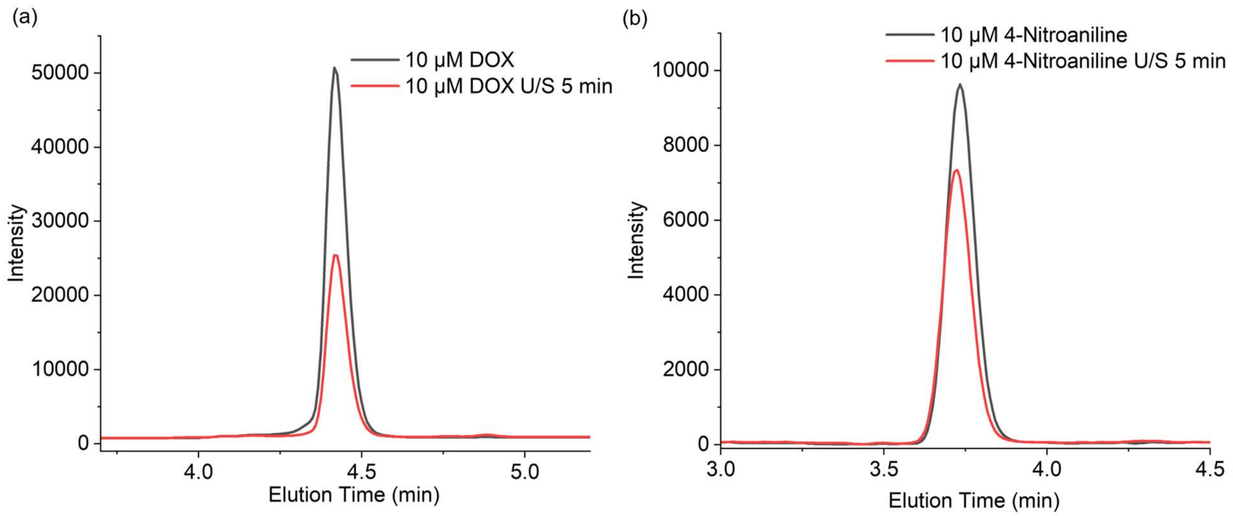

Figure S15. HPLC spectra of DOX (a) and 4-Nitroaniline (b) before and after 5 min LITUS irradiation. $[DOX]_0$ = 10 µM. $[4\text{-Nitroaniline}]_0$ = 10 µM.

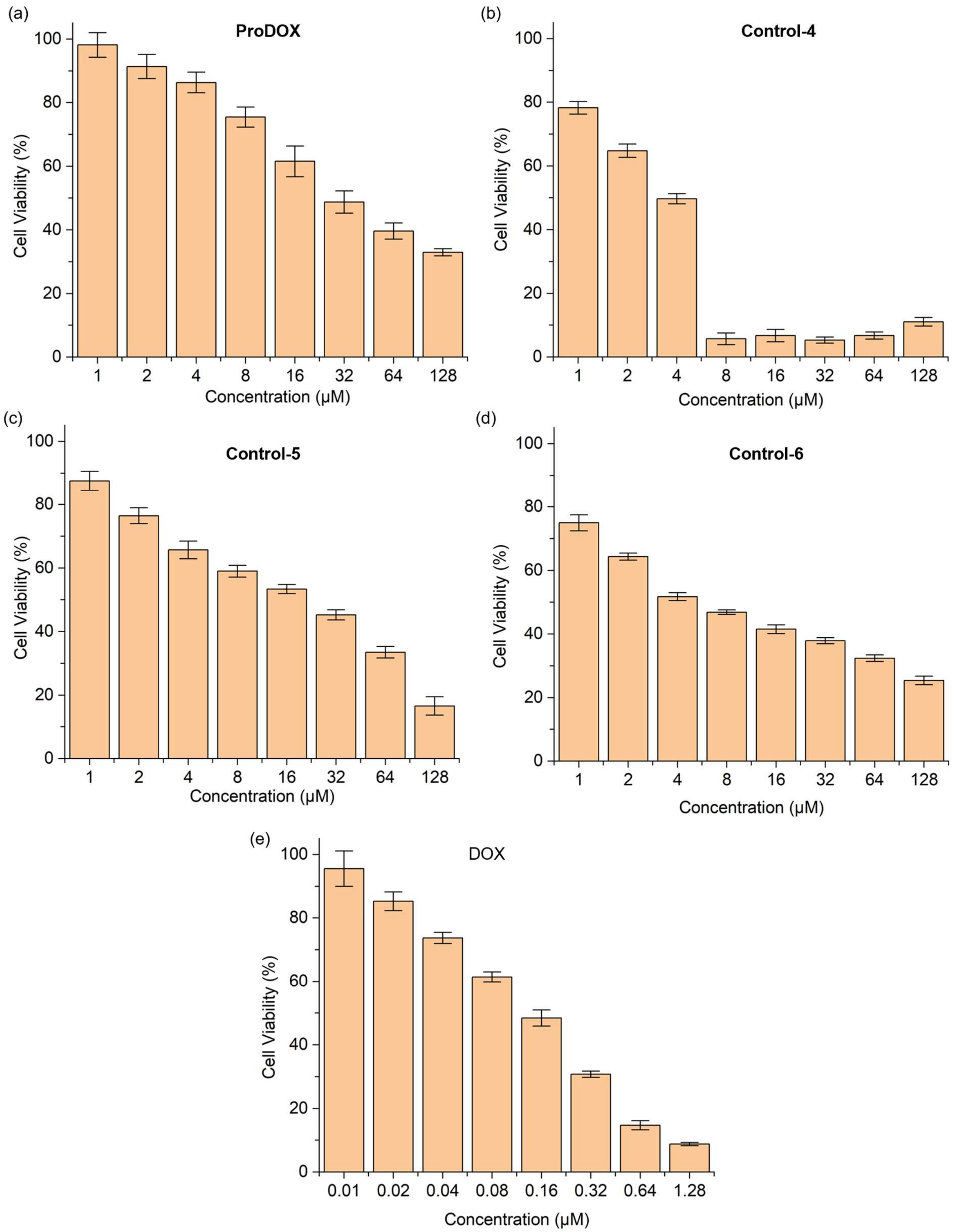

Figure S16. Cytotoxicity of **ProDOX** (a), **Control-4** (b), **Control-5** (c), **Control-6** (d), and DOX drug molecules (e) toward HeLa cells are assessed through MTT assays in vitro.

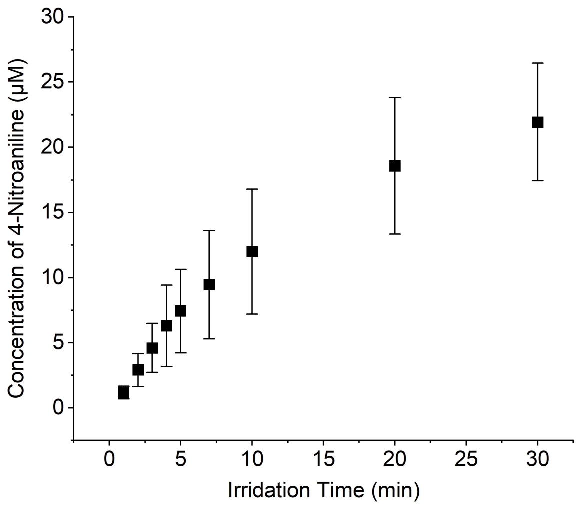

Figure S17. Concentration of 4-nitroaniline released from a 50 μM **Pro1** solution in PBS over time during ultrasound exposure through chicken breast. Ultrasound condition: 1 MHz, 3.0 W/cm^2, 50% duty cycle.

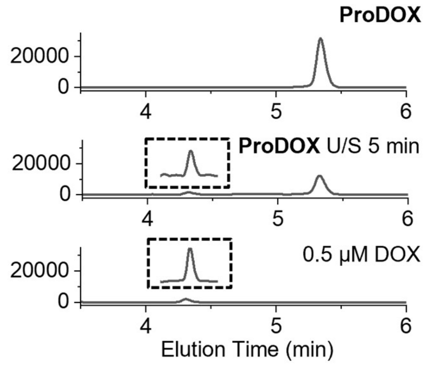

Figure S18. HPLC spectra of 10 μM **ProDOX** solution before and after 5 min of LITUS irradiation applied through chicken breast. As a reference, HPLC spectra of 0.5 μM DOX is shown. Ultrasound condition: 1 MHz, 3.0 W/cm^2, 50% duty cycle.

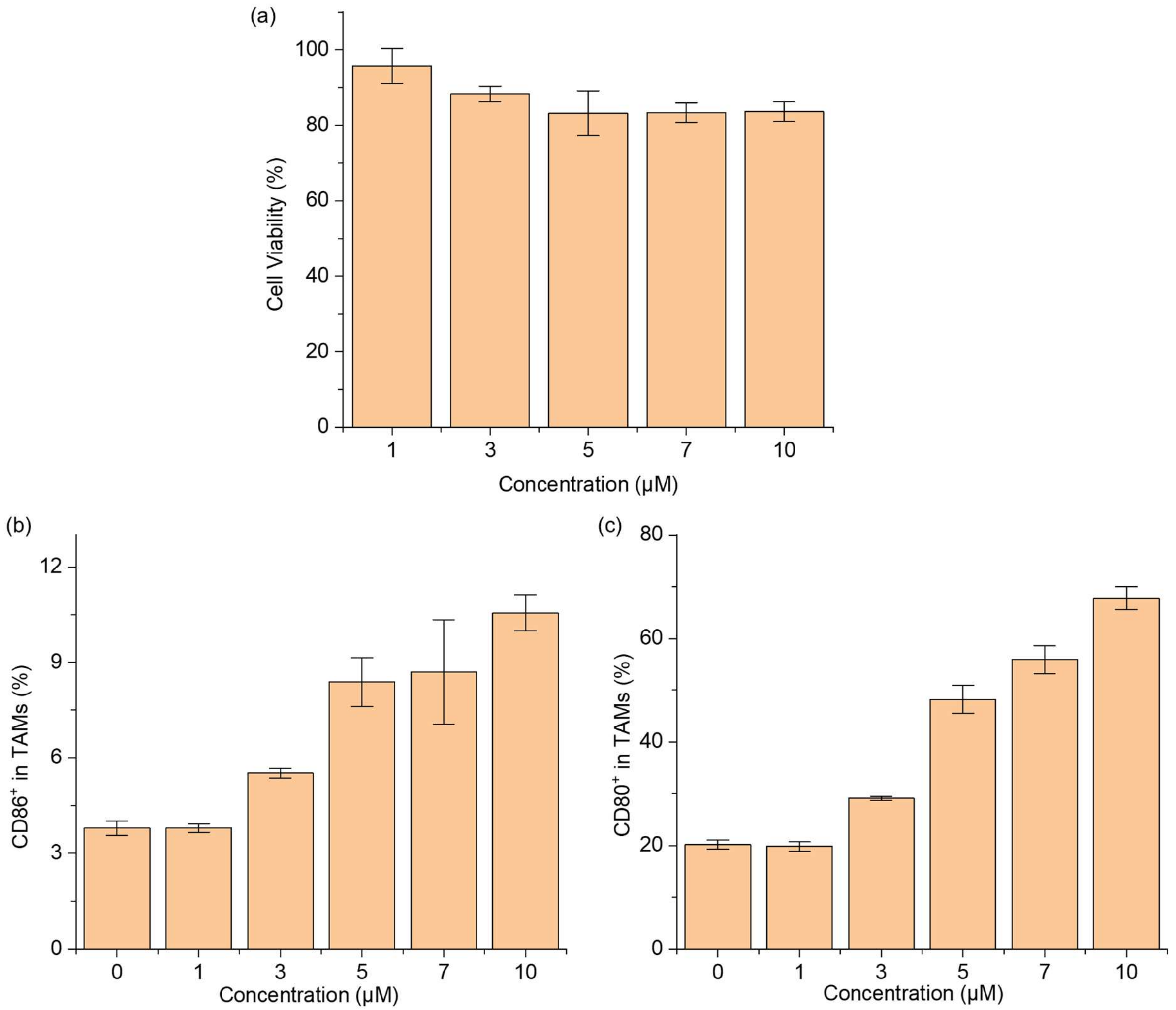

Figure S19. (a) Cytotoxicity of **ProR848** toward tumor associated macrophages (TAMs) are assessed through MTT assays in vitro. (b, c) Flow cytometry analysis to determine the concentration of **ProR848** that does not activate tumor-associated macrophages. Compared to the negative control (0 μM **ProR848**), 1 μM **ProR848** showed no significant activation of TAMs and was therefore selected as the working concentration for subsequent experiments. (b) CD86, (c) CD80.

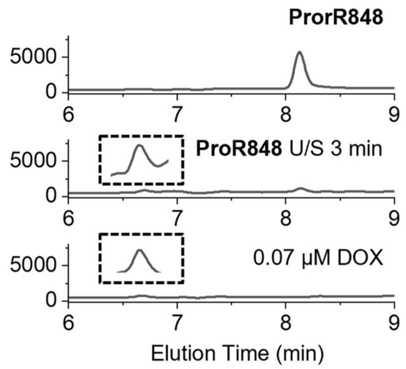

Figure S20. HPLC spectra of 1 μM **ProR848** solution before and after 3 min of LITUS irradiation. As a reference, HPLC spectra of 0.07 μM R848 is shown. Ultrasound condition: 1 MHz, 1.0 W/cm^2, 50% duty cycle.

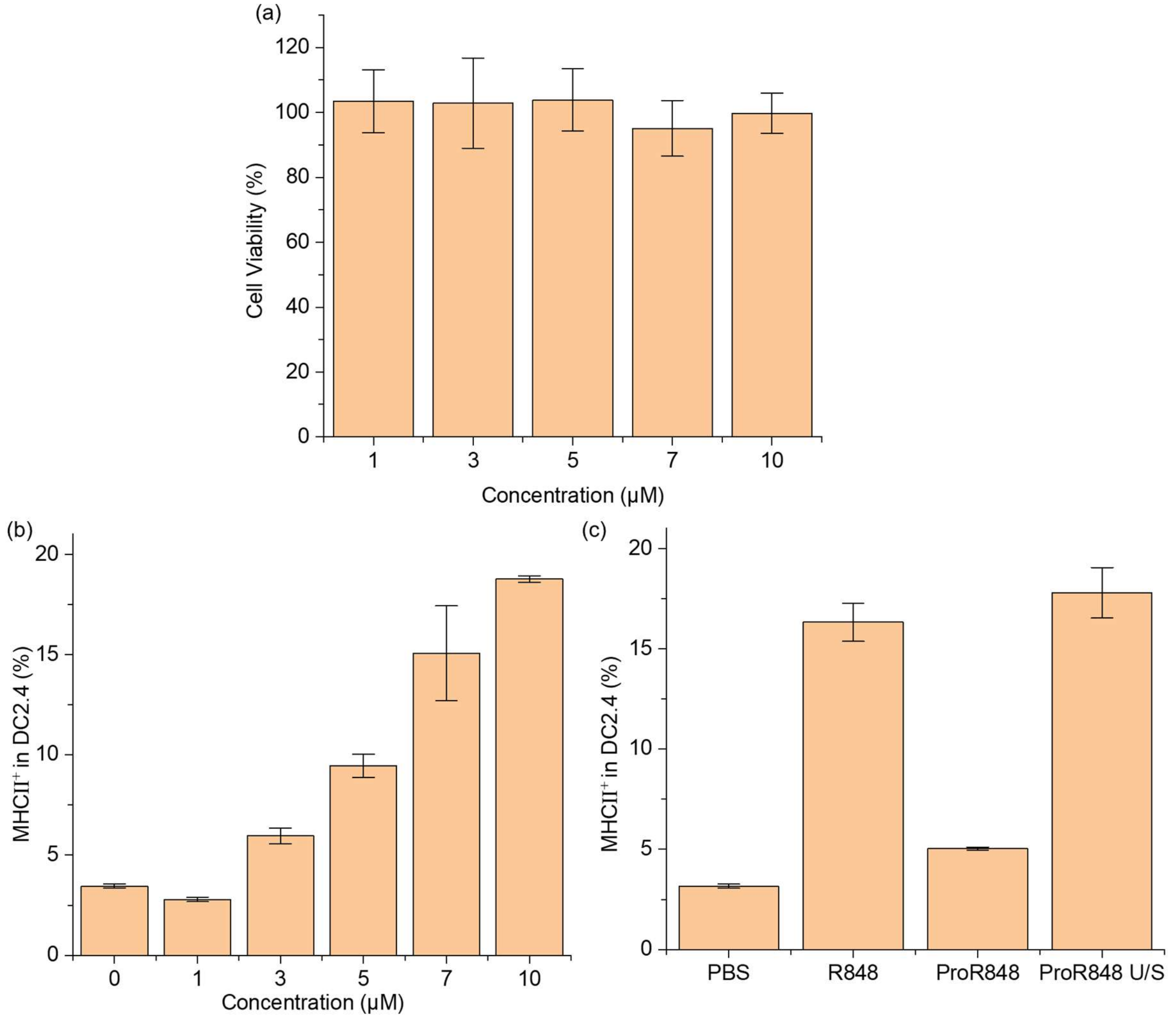

Figure S21. (a) Cytotoxicity of **ProR848** toward DC2.4 cell lines are assessed through MTT assays in vitro. (b) Flow cytometry analysis of MHCII marker to determine the concentration of **ProR848** that does not activate DC2.4 cells. Compared to the negative control (0 μM **ProR848**), 1 μM

ProR848 showed no significant activation of DC2.4 cell lines and was therefore selected as the working concentration for subsequent experiments. (c) Flow cytometry analysis of MHCII expression in dendritic cells before and after LITUS treatment. The significant upregulation of MHCII in the **ProR848** U/S group indicates effective maturation of dendritic cells.

3. MTT Experiments

3.1 Cytotoxicity Assay (Figure S16, Figure S19a, Figure S21a):

Step 1: Cells were seeded in a 96-well plate at 5×10^4 cells/mL in 100 μL of Cell medium (10% FBS, 1% penicillin/streptomycin; RPMI-1640 for Hela cells and DC2.4 cells, DMEM for Raw264.7 cells) and incubated at 37 °C with 5% CO_2 for 24 hours.

Step 2: The culture medium was replaced with fresh medium containing different concentrations of chemicals, and incubated (Figure S16 for 48 hours; Figure S19a and Figure S21a for 24 hours).

Step 3: The supernatant was replaced with 100 μL of MTT solution (0.5 mg/mL in cell medium with 10% FBS) and incubated at 37 °C for 4 hours.

Step 4: The supernatant was removed, 100 μL of DMSO was added to dissolve the formazan crystals, and the plate was shaken for 60 seconds.

Step 5: Absorbance at 490 nm was measured using a microplate reader, and the viability of the blank control group was set as 100% for normalization.

3.2. Cytotoxicity of Ultrasound-Treated **ProDOX**, **Control-4**, **Control-5**, and **Control-6** (Figure 4c, and Figure 5d):

Step 1: HeLa cells were seeded in a 96-well plate at 5×10^4 cells/mL in 100 μL of RPMI-1640 medium (10% FBS, 1% penicillin/streptomycin) and incubated at 37 °C with 5% CO_2 for 24 hours.

Step 2: 100 μL of sonicated 10 μM **ProDOX** solution, **Control-4**, **Control-5**, and **Control-6** were added to each well, including a blank control (100 μL of PBS containing 0.1% DMF, matching the DMF concentration in other test solutions).

Step 3: After 2 hours, 200 μL of fresh RPMI-1640 medium was added, and the cells were further incubated for 46 hours.

Step 4: The MTT assay was performed as described in the dark cytotoxicity assay.

4. Immune Cell Activation Experiments

4.1 Tumor associated macrophages (TAMs) Activation Assay (Figure S19, Figure 6):

Step 1: Raw264.7 cells were seeded in a 6-well plate at 3×10^5 cells/mL in 3 mL of DMEM medium (10% FBS, 1% penicillin/streptomycin) with 20 ng/mL IL-4 and incubated at 37 °C with 5% CO_2 for 24 hours to obtain TAMs.

Step 2: 1 mL of 3 mins sonicated 1 μM **ProR848** solution (G4) were added to each well, including the control (G1: PBS, G2: 0.07 μM R848, G3: 1 μM **ProR848**).

Step 3: After 2 hours, 2 mL of fresh DMEM medium was added, and the cells were further incubated for 22 hours.

Step 4: The cells were harvested by scraping for flow cytometry analysis, and the culture supernatants were collected for ELISA assays

4.2 Dendritic cells Activation Assay (Figure S21):

Step 1: DC2.4 cells were seeded in a 6-well plate at 3 × 10^5 cells/mL in 3 mL of RPMI-1640 medium (10% FBS, 1% penicillin/streptomycin) and incubated at 37 °C with 5% CO_2 for 24 hours.

Step 2: 1 mL of 3 mins sonicated 1 µM **ProR848** solution (G4) were added to each well, including the control (G1: 1 mL PBS, G2: 0.07 µM R848, G3: 1 µM **ProR848**).

Step 3: After 2 hours, 2 mL of fresh RPMI-1640 medium was added, and the cells were further incubated for 22 hours.

Step 4: The cells were harvested by scraping for flow cytometry analysis.

5. Synthetic Details

Scheme S1. Synthesis Route of **Pro1**.

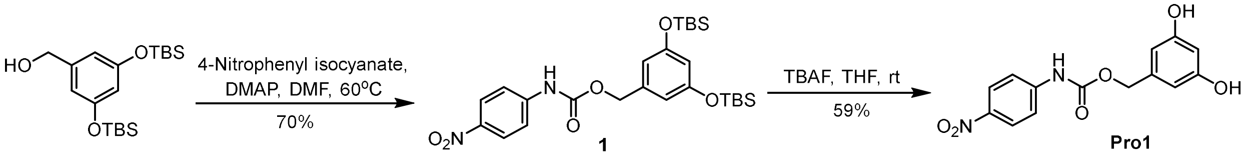

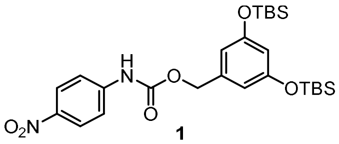

Compound 1. A round bottom flask equipped with a stir bar was charged with 2-Ethyl-4,6-bis(tert-butyldimethylsiloxy)phenol (Ref : *Angew. Chem. Int. Ed.* **2020**, 59, 21546.) (0.3 g, 0.776 mmol), 4-nitrophenyl isocyanate (0.153 g, 0.931mmol), DMAP (0.189 g, 1.55 mmol), and anhydrous DMF (2 mL). After reacting at 60 °C overnight, the mixture was extracted with ether (150 mL). The organic fraction was washed with 10% NH_4Cl (50 mL), water (50 mL), and brine (50 mL). The organic fraction was dried over Na_2SO_4, filtered, and concentrated under reduced pressure to yield a crude mixture. The crude product was purified by column chromatography (5–25% EtOAC/hexanes) to provide the title compound as a white solid (0.304 g, 70%). R_f = 0.25 (hexanes : EtOAc 10 : 1).).^{1}H NMR (400 MHz, $CDCl_3$) δ 8.21 (d, J = 9.1 Hz, 2H), 7.56 (d, J = 9.2 Hz, 2H), 6.98 (s, 1H), 6.49 (d, J = 2.2 Hz, 2H), 6.31 (t, J = 2.2 Hz, 1H), 5.10 (s, 2H), 0.98 (s, 18H), 0.20 (s, 12H). ^{13}C NMR (100 MHz, $CDCl_3$) δ 156.95, 152.65, 143.90, 137.30, 125.39, 117.91, 113.43, 112.23, 67.62, 25.80, 18.35, -4.24. HRMS (DART/Orbitrap) m/z: Calcd for $C_{26}H_{40}N_2O_6Si_2$ $[M+H]^+$, 533.2497; Found 533.2484.

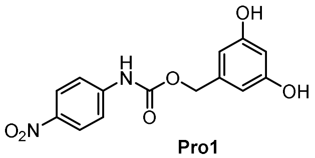

Pro1

Pro1. A flame-dried round bottom flask equipped with a stir bar was charged with **1** (0.304 g, 0.572 mmol) and 4 mL anhydrous THF. The mixture was cooled to 0 °C in an ice bath, followed by the dropwise addition of TBAF (1 M in THF, 1.26 mL, 1.26 mmol). The stirred reaction was allowed to warm up to room temperature. After one hour, the reaction was quenched with NH_4Cl (10 mL) and extracted with EtOAc (100 mL). The organic fraction was washed with water (50 mL) and brine (50 mL). The organic fraction was dried over Na_2SO_4, filtered, and concentrated under reduced pressure. The crude product was purified by column chromatography (5–15% MeOH/DCM) to yield the title compound as a white solid (102 mg, 59%). R_f = 0.15 (DCM : MeOH 10 : 1). ^{1}H NMR (400 MHz, DMSO-d_6) δ 10.49 (s, 1H), 9.29 (s, 2H), 8.21 (d, J = 9.3 Hz, 2H), 7.74 – 7.67 (m, 2H), 6.26 (d, J = 2.2 Hz, 2H), 6.16 (t, J = 2.2 Hz, 1H), 5.02 (s, 2H). ^{13}C NMR (100 MHz, DMSO-d_6) δ 158.49, 153.13, 145.65, 141.71, 139.01, 125.12, 117.65, 105.85, 102.15, 66.32. HRMS (DART/Orbitrap) m/z: Calcd for $C_{14}H_{12}N_2O_6$ $[M+H]^+$, 305.0761; Found 305.0768.

Scheme S2. Synthesis Route of **Control-1**.

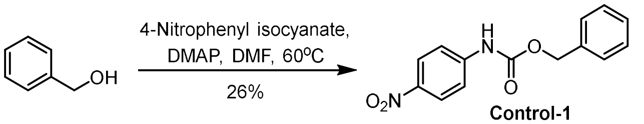

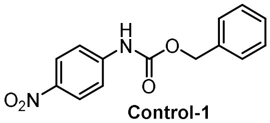

Control-1

Control-1. A round bottom flask equipped with a stir bar was charged with benzyl alcohol (0.2 g, 1.85 mmol), 4-nitrophenyl isocyanate (0.728 g, 4.44mmol), DMAP (0.542g, 4.44 mmol), and anhydrous DMF (2 mL). After reacting at 60 °C overnight, the mixture was extracted with ether (150 mL). The organic fraction was washed with 10% NH_4Cl (50 mL), water (50 mL), and brine (50 mL). The organic fraction was dried over Na_2SO_4, filtered, and concentrated under reduced pressure to yield a crude mixture. The crude product was purified by column chromatography (10–30% EtOAC/hexanes) to provide the title compound as a white solid (0.132 g, 26%). R_f = 0.60 (hexanes : EtOAc 2 : 1). ^{1}H NMR (400 MHz, $CDCl_3$) δ 8.20 (d, J = 9.2 Hz, 2H), 7.55 (d, J = 9.3 Hz, 2H), 7.46 – 7.32 (m, 5H), 7.01 (s, 1H), 5.24 (s, 2H). ^{13}C NMR (100 MHz, $CDCl_3$) δ 152.72, 143.88, 143.26, 135.45, 128.89, 128.86, 128.64, 125.39, 117.90, 67.90.

Scheme S3. Synthesis Route of **Control-2**.

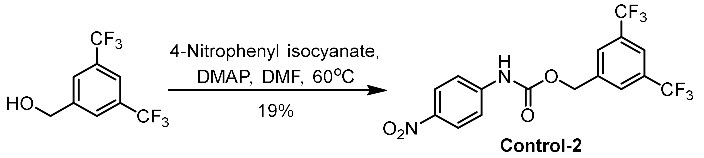

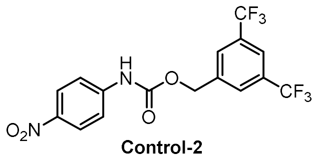

Control-2

Compound 4. A round bottom flask equipped with a stir bar was charged with 2-(3,5-Bis(trifluoromethyl)phenyl)ethanol (0.3 g, 1.23 mmol), 4-nitrophenyl isocyanate (0.484 g, 2.95 mmol), DMAP (0.36g, 2.95 mmol), and anhydrous DMF (2 mL). After reacting at 60 °C overnight, the mixture was extracted with ether (150 mL). The organic fraction was washed with 10% NH_4Cl (50 mL), water (50 mL), and brine (50 mL). The organic fraction was dried over Na_2SO_4, filtered, and concentrated under reduced pressure to yield a crude mixture. The crude product was purified by column chromatography (10–30% EtOAC/hexanes) to provide the title compound as a white solid (0.097 g, 19%). R_f = 0.60 (hexanes : EtOAc 2 : 1). ^{1}H NMR (400 MHz, DMSO-d_6) δ 10.57 (s, 1H), 8.26 – 8.17 (m, 4H), 8.11 (s, 1H), 7.72 (d, J = 9.8 Hz, 2H), 5.39 (s, 2H). ^{13}C NMR (100 MHz, DMSO-d_6) δ 152.77, 145.38, 141.87, 139.62, 130.53, 128.87, 125.13, 124.63, 121.92, 117.79, 64.87. HRMS (DART/Orbitrap) m/z: Calcd for $C_{16}H_{10}F_6N_2O_4$ $[M+H]^+$, 409.0609; Found 409.0617.

Scheme S4. Synthesis Route of **Control-3**.

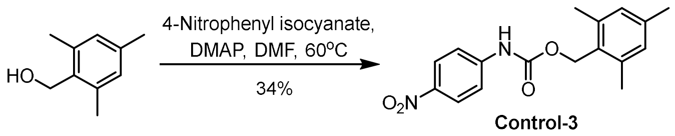

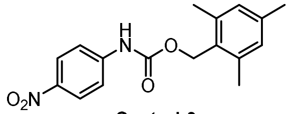

Control-3

Control-3. A round bottom flask equipped with a stir bar was charged with 2,4,6-trimethylbenzyl alcohol (0.2 g, 1.33 mmol), 4-nitrophenyl isocyanate (0.524 g, 3.2 mmol), DMAP (0.39g, 3.2 mmol), and anhydrous DMF (2 mL). After reacting at 60 °C overnight, the mixture was extracted with ether (150 mL). The organic fraction was washed with 10% NH_4Cl (50 mL), water (50 mL), and brine (50 mL). The organic fraction was dried over Na_2SO_4, filtered, and concentrated under reduced pressure to yield a crude mixture. The crude product was purified by column chromatography (10–30% EtOAC/hexanes) to provide the title compound as a white solid (0.141 g, 34%). R_f = 0.40 (hexanes : EtOAc 4 : 1). ^{1}H NMR (400 MHz, $CDCl_3$) δ 8.19 (d, J = 9.5 Hz, 2H), 7.53 (d, J = 9.3 Hz, 2H), 6.92 (d, J = 10.1 Hz, 3H), 5.32 (s, 2H), 2.40 (s, 6H), 2.29 (s, 3H). ^{13}C NMR (100 MHz, $CDCl_3$) δ 152.97, 144.00, 143.17, 139.16, 138.42, 129.38, 128.51, 125.38,

117.79, 62.57, 21.17, 19.67. HRMS (DART/Orbitrap) m/z: Calcd for $C_{17}H_{22}N_3O_4$ $[M+NH_4]^+$, 332.1593; Found 332.1604.

Scheme S5. Synthesis Route of **ProDOX**.

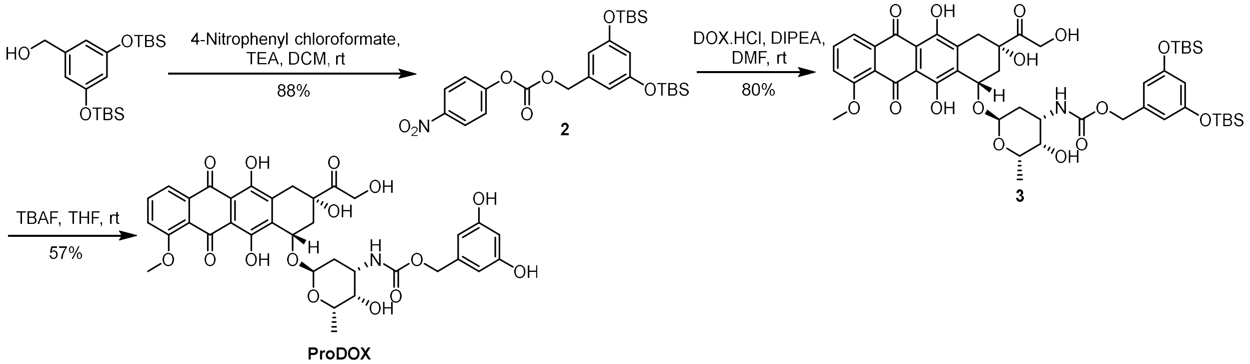

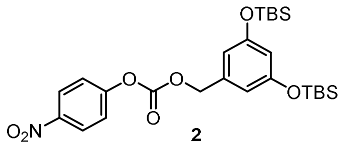

Compound 2. A round bottom flask equipped with a stir bar was charged with 2-Ethyl-4,6-bis(tert-butyldimethylsiloxy)phenol (0.3 g, 0.81 mmol), 4-nitrophenyl chloroformate (0.18 g, 0.89 mmol), and anhydrous THF (5 mL). The mixture was cooled to 0 °C in an ice bath before adding triethylamine (TEA, 0.091 g, 0.90 mmol) dropwise. After overnight reaction at room temperature, the mixture was extracted with EtOAc (150 mL). The organic fraction was washed with 10% NH_4Cl (150 mL), water (150 mL), and brine (150 mL). The organic phase was dried over Na_2SO_4, filtered, and concentrated under reduced pressure. The product was separated by column chromatography (5-15 % EtOAc/hexanes) to provide the title compound as a white solid (0.38 g, 88%). $R_f = 0.25$ (hexanes : EtOAc 10 : 1). ^1H NMR (400 MHz, $CDCl_3$) δ 8.28 (d, J = 9.3 Hz, 2H), 7.38 (d, J = 9.3 Hz, 2H), 6.52 (d, J = 2.2 Hz, 2H), 6.34 (t, J = 2.2 Hz, 1H), 5.17 (s, 2H), 0.98 (s, 19H), 0.20 (s, 12H). ^{13}C NMR (100 MHz, $CDCl_3$) δ 157.01, 155.72, 152.55, 145.56, 136.18, 125.46, 121.94, 113.54, 112.63, 70.81, 25.80, 18.36, -4.24.

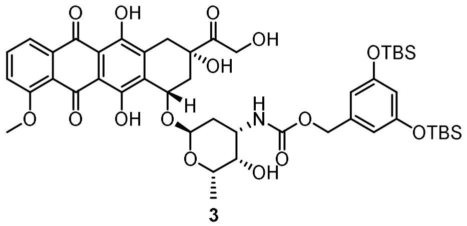

Compound 3. A round bottom flask equipped with a stir bar was charged with compound **2** (0.122 g, 0.228 mmol), DOX.HCl (0.120 g, 0.206 mmol), and anhydrous DMF (5 mL). The mixture was cooled to 0 °C in an ice bath before adding N,N-Diisopropylethylamine (DIPEA, 0.058 g, 0.449 mmol) dropwise. After overnight reaction at room temperature, the mixture was extracted with ether (150 mL). The organic fraction was washed with 10% NH_4Cl (150 mL), water (150 mL),

and brine (150 mL). The organic phase was dried over Na_2SO_4, filtered, and concentrated under reduced pressure. The product was separated by column chromatography (15-55 % acetone/hexanes) to provide the title compound as a red solid (0.13 g, 80%). R_f = 0.3 (hexanes : acetone 2 : 1). ^{1}H NMR (400 MHz, $CDCl_3$) δ 8.06 (dd, J = 7.7, 1.0 Hz, 1H), 7.79 (t, J = 8.1 Hz, 1H), 7.40 (d, J = 8.5 Hz, 1H), 6.39 (d, J = 2.2 Hz, 2H), 6.24 (s, 1H), 5.51 (d, J = 3.9 Hz, 1H), 5.31 (d, J = 7.6 Hz, 1H), 5.18 – 5.03 (m, 1H), 4.90 (s, 2H), 4.77 (d, J = 4.5 Hz, 2H), 4.54 (s, 1H), 4.09 (s, 4H), 3.87 (s, 1H), 3.68 (d, J = 7.6 Hz, 1H), 3.45 – 3.24 (m, 1H), 3.15 – 2.79 (m, 2H), 2.47 – 1.65 (m, 5H), 1.31 – 1.21 (m, 5H), 0.94 (s, 20H), 0.16 (s, 13H). ^{13}C NMR (100 MHz, $CDCl_3$) δ 214.02, 186.83, 161.19, 156.72, 156.33, 155.80, 138.32, 135.89, 135.64, 133.71, 121.01, 119.98, 118.56, 113.02, 111.74, 111.56, 100.84, 69.79, 69.71, 67.39, 66.65, 65.70, 65.65, 56.80, 49.61, 34.16, 29.84, 25.81, 18.30, 16.99, -4.28. HRMS (DART/Orbitrap) m/z: Calcd for $C_{47}H_{62}NO_{15}Si_2$[M-H]$^-$, 936.3663; Found 936.3669.

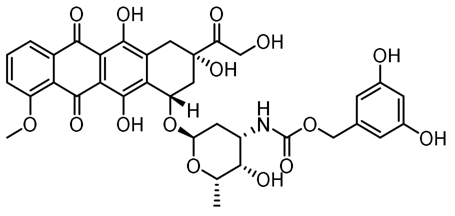

ProDOX

ProDOX. A flame-dried round bottom flask equipped with a stir bar was charged with **3** (0.13 g, 0.139 mmol) and 4 mL anhydrous THF. The mixture was cooled to 0 °C in an ice bath, followed by the dropwise addition of TBAF (1 M in THF, 0.305 mL, 0.305 mmol). The stirred reaction was allowed to warm up to room temperature. After one hour, the reaction was quenched with NH_4Cl (10 mL) and extracted with DCM (100 mL). The organic fraction was washed with water (50 mL) and brine (50 mL). The organic fraction was dried over Na_2SO_4, filtered, and concentrated under reduced pressure. The crude product was purified by column chromatography (5–15% MeOH/DCM) to yield the title compound as a white solid (56 mg, 57%). R_f = 0.3 (DCM : MeOH 10 : 1). ^{1}H NMR (400 MHz, DMSO-d_6) δ 9.17 (s, 2H), 8.31 (s, 1H), 7.88 (q, J = 4.1, 3.3 Hz, 2H), 7.62 (dd, J = 6.1, 3.8 Hz, 1H), 6.82 (d, J = 8.0 Hz, 1H), 6.13 (d, J = 2.2 Hz, 2H), 6.08 (t, J = 2.2 Hz, 1H), 5.43 (s, 1H), 5.22 (d, J = 3.6 Hz, 1H), 4.93 (t, J = 4.4 Hz, 1H), 4.87 – 4.64 (m, 4H), 4.57 (d, J = 5.8 Hz, 2H), 4.15 (t, J = 6.9 Hz, 1H), 3.98 (s, 4H), 3.72 (s, 1H), 3.45 (dd, J = 5.8, 2.6 Hz, 1H), 3.05 – 2.81 (m, 2H), 2.31 – 2.03 (m, 3H), 1.85 (td, J = 12.9, 3.8 Hz, 1H), 1.48 (dd, J = 12.4, 4.5 Hz, 1H), 1.23 (s, 1H), 1.13 (d, J = 6.5 Hz, 3H). ^{13}C NMR (100 MHz, DMSO-d_6) δ 213.81, 186.53, 160.78, 158.30, 156.09, 155.32, 139.05, 136.20, 135.51, 134.07, 120.00, 119.72, 118.99, 110.77, 110.64, 101.73, 100.31, 79.18, 74.95, 69.86, 68.04, 66.68, 65.08, 56.58, 47.15, 36.59, 32.07, 30.70, 17.03. HRMS (DART/Orbitrap) m/z: Calcd for $C_{35}H_{34}NO_{15}$[M-H]$^-$, 708.1934; Found 708.1942.

Scheme S6. Synthesis Route of **Control-4**.

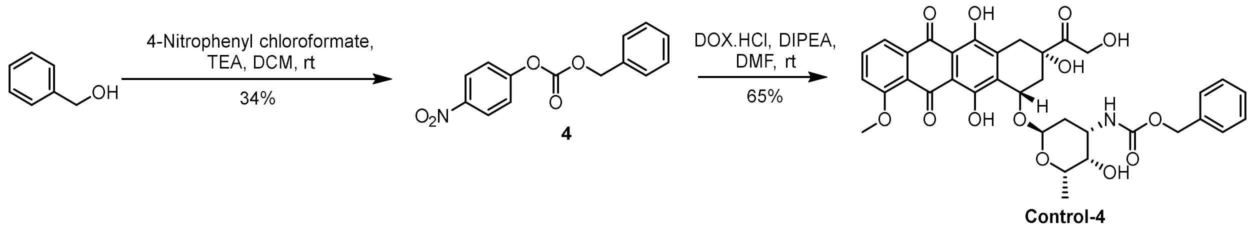

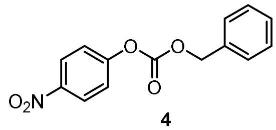

4

Compound 4. A round bottom flask equipped with a stir bar was charged with benzyl alcohol (0.268 g, 2.48 mmol), 4-nitrophenyl chloroformate (0.5 g, 2.48 mmol), and anhydrous THF (5 mL). The mixture was cooled to 0 °C in an ice bath before adding N,N-diisopropylethylamine (DIPEA, 0.336 g, 0.60 mmol) dropwise. After overnight reaction at room temperature, the mixture was extracted with EtOAc (150 mL). The organic fraction was washed with 10% NH_4Cl (150 mL), water (150 mL), and brine (150 mL). The organic phase was dried over Na_2SO_4, filtered, and concentrated under reduced pressure. The product was separated by column chromatography (5-25 % EtOAc/hexanes) to provide the title compound as a white solid (0.23 g, 34%). R_f = 0.4 (hexanes : EtOAc 4 : 1). ^{1}H NMR (400 MHz, $CDCl_3$) δ 8.27 (d, J = 9.3 Hz, 2H), 7.50 – 7.34 (m, 7H), 5.31 (s, 2H). ^{13}C NMR (100 MHz, $CDCl_3$) δ 155.68, 152.60, 145.56, 134.33, 129.23, 128.96, 128.82, 125.45, 121.92, 71.15.

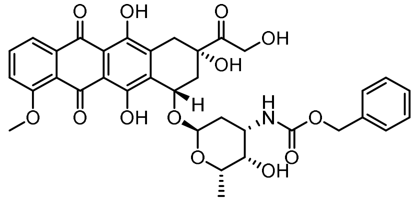

Control-4

Control-4. A round bottom flask equipped with a stir bar was charged with compound **4** (0.023 g, 0.083 mmol), DOX.HCl (0.04 g, 0.069 mmol), and anhydrous DMF (5 mL). The mixture was cooled to 0 °C in an ice bath before adding N,N-diisopropylethylamine (DIPEA, 0.021 g, 0.166 mmol) dropwise. After overnight reaction at room temperature, the mixture was extracted with ether (100 mL). The organic fraction was washed with 10% NH_4Cl (50 mL), water (50 mL), and brine (50 mL). The organic phase was dried over Na_2SO_4, filtered, and concentrated under reduced pressure. The product was separated by column chromatography (2-10 % MeOH/DCM) to provide the title compound as a red solid (0.03 g, 65%). R_f = 0.55 (MeOH/DCM 10 : 1). ^{1}H NMR (400 MHz, $CDCl_3$) δ 8.03 (d, J = 7.7 Hz, 1H), 7.78 (t, J = 8.1 Hz, 1H), 7.39 (d, J = 8.5 Hz, 1H), 7.30 (d, J = 5.8 Hz, 6H), 5.50 (d, J = 3.9 Hz, 1H), 5.29 (s, 1H), 5.13 (d, J = 8.6 Hz, 1H), 5.03 (s, 2H), 4.82 – 4.70 (m, 2H), 4.54 (s, 1H), 4.08 (s, 4H), 3.88 (d, J = 8.6 Hz, 1H), 3.67 (s, 1H), 3.27 (dd, J = 18.9, 1.9 Hz, 1H), 3.10 – 2.92 (m, 2H), 2.43 – 2.10 (m, 3H), 2.06 – 1.82 (m, 3H), 1.77 (td, J = 13.1, 4.1 Hz, 5H), 1.38 (s, 2H), 1.35 – 1.15 (m, 10H), 0.99 – 0.74 (m, 4H). ^{13}C NMR (100 MHz, $CDCl_3$) δ

213.99, 187.24, 161.20, 156.32, 155.79, 136.46, 135.92, 133.71, 128.65, 128.27, 128.19, 121.02, 120.00, 119.76, 118.59, 111.75, 111.57, 100.87, 69.84, 69.73, 67.40, 66.93, 65.69, 65.46, 56.82, 47.12, 34.15, 30.32, 30.10, 16.98. HRMS (DART/Orbitrap) m/z: Calcd for $C_{35}H_{34}NO_{13}$[M-H]$^-$, 676.2036; Found 676.2042.

Scheme S7. Synthesis Route of **Control-5**.

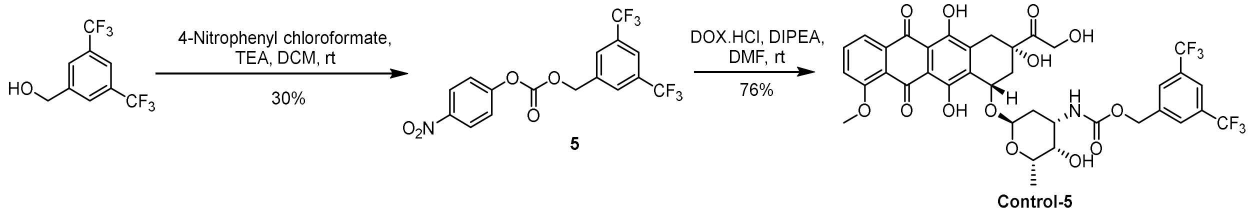

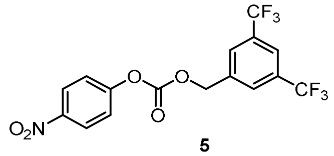

5

Compound 5. A round bottom flask equipped with a stir bar was charged with 2-(3,5-Bis(trifluoromethyl)phenyl)ethanol (2g, 8.19 mmol), 4-nitrophenyl chloroformate (1.98 g, 9.83 mmol), and anhydrous THF (30 mL). The mixture was cooled to 0 °C in an ice bath before adding N,N-diisopropylethylamine (DIPEA, 1.376 g, 10.65 mmol) dropwise. After overnight reaction at room temperature, the mixture was extracted with EtOAc (150 mL). The organic fraction was washed with 10% NH_4Cl (150 mL), water (150 mL), and brine (150 mL). The organic phase was dried over Na_2SO_4, filtered, and concentrated under reduced pressure. The product was separated by column chromatography (5-25 % EtOAc/hexanes) to provide the title compound as a white solid (1g, 30%). R_f = 0.4 (hexanes : EtOAc 4 : 1). ^{1}H NMR (400 MHz, $CDCl_3$) δ 8.30 (d, J = 9.1 Hz, 2H), 7.91 (d, J = 3.2 Hz, 3H), 7.41 (d, J = 9.3 Hz, 2H), 5.41 (s, 2H). ^{13}C NMR (100 MHz, $CDCl_3$) δ 155.35, 152.37, 145.79, 136.87, 132.99, 125.56, 124.51, 123.13, 123.05, 121.84, 121.80, 69.00.

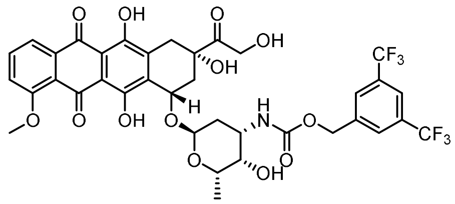

Control-5

Control-5. A round bottom flask equipped with a stir bar was charged with compound **5** (0.127 g, 0.31 mmol), DOX.HCl (0.15 g, 0.26 mmol), and anhydrous DMF (3 mL). The mixture was cooled to 0 °C in an ice bath before adding N,N-diisopropylethylamine (DIPEA, 0.08 g, 0.62 mmol) dropwise. After overnight reaction at room temperature, the mixture was extracted with ether (100 mL). The organic fraction was washed with 10% NH_4Cl (50 mL), water (50 mL), and brine (50 mL). The organic phase was dried over Na_2SO_4, filtered, and concentrated under reduced pressure. The product was separated by column chromatography (2-10 % MeOH/DCM) to provide the title

compound as a red solid (0.16 g, 76%). R_f = 0.5 (MeOH/DCM 10 : 1). ^{1}H NMR (400 MHz, $CDCl_3$) δ 8.04 (dd, J = 7.7, 1.1 Hz, 1H), 7.83 – 7.68 (m, 4H), 7.40 (dd, J = 8.5, 1.1 Hz, 1H), 5.51 (d, J = 4.0 Hz, 1H), 5.30 (dd, J = 4.1, 2.1 Hz, 1H), 5.23 (d, J = 8.6 Hz, 1H), 5.14 (s, 2H), 4.75 (s, 2H), 4.50 (s, 1H), 4.16 (q, J = 6.6 Hz, 1H), 4.08 (s, 3H), 3.88 (s, 1H), 3.68 (s, 1H), 3.28 (dd, J = 18.9, 1.9 Hz, 1H), 3.01 (d, J = 18.9 Hz, 2H), 2.34 (dt, J = 14.5, 2.2 Hz, 1H), 2.18 (dd, J = 14.6, 4.2 Hz, 1H), 1.97 – 1.86 (m, 2H), 1.79 (td, J = 13.2, 4.1 Hz, 1H), 1.30 (d, J = 6.6 Hz, 3H). ^{13}C NMR (100 MHz, $CDCl_3$) δ 213.90, 187.30, 186.87, 161.22, 156.30, 155.79, 154.99, 139.19, 135.97, 135.65, 133.70, 133.57, 132.18, 131.84, 127.89, 124.63, 122.14, 121.01, 120.03, 118.61, 111.79, 111.62, 100.80, 69.95, 69.69, 67.32, 65.68, 65.14, 56.83, 47.29, 35.79, 34.16, 30.33, 16.95. HRMS (DART/Orbitrap) m/z: Calcd for $C_{37}H_{32}F_6NO_{13}[M-H]^-$, 812.1783; Found 812.1788.

Scheme S8. Synthesis Route of **Control-6**.

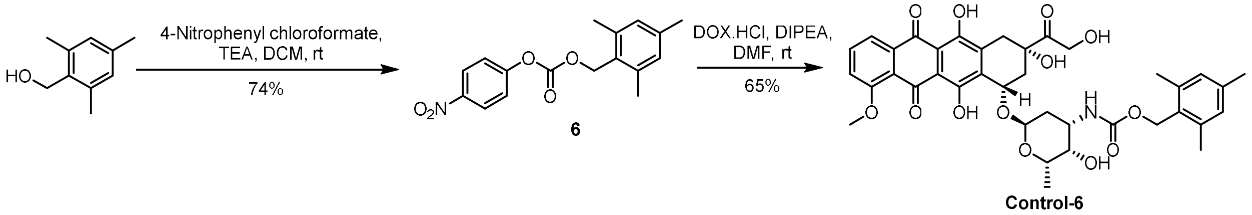

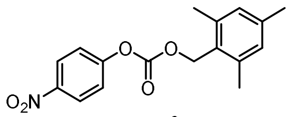

6

Compound 6. A round bottom flask equipped with a stir bar was charged with 2,4,6-trimethylbenzyl alcohol (0.5g, 3.32 mmol), 4-nitrophenyl chloroformate (0.806 g, 4 mmol), and anhydrous THF (10 mL). The mixture was cooled to 0 °C in an ice bath before adding triethylamine (TEA, 0.404 g, 4 mmol) dropwise. After overnight reaction at room temperature, the mixture was extracted with EtOAc (150 mL). The organic fraction was washed with 10% NH_4Cl (150 mL), water (150 mL), and brine (150 mL). The organic phase was dried over Na_2SO_4, filtered, and concentrated under reduced pressure. The product was separated by column chromatography (5-25 % EtOAc/hexanes) to provide the title compound as a white solid (0.78g, 74%). R_f = 0.4 (hexanes : EtOAc 4 : 1). ^{1}H NMR (400 MHz, $CDCl_3$) δ 8.29 (d, J = 9.3 Hz, 2H), 7.40 (d, J = 9.2 Hz, 2H), 6.94 (s, 2H), 5.43 (s, 2H), 2.44 (s, 6H), 2.32 (s, 3H). ^{13}C NMR (100 MHz, $CDCl_3$) δ 155.80, 152.78, 145.47, 139.45, 138.61, 129.42, 127.73, 125.42, 121.92, 66.04, 21.20, 19.72. HRMS (DART/Orbitrap) m/z: Calcd for $C_{17}H_{16}NO_6$ $[M-H+O]^-$, 330.0983; Found 330.0959.

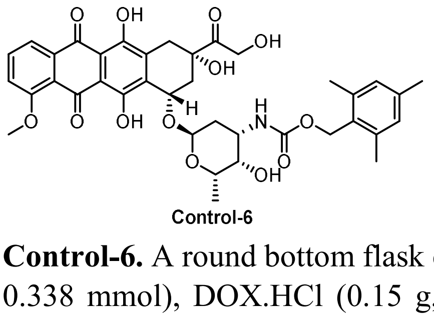

Control-6

Control-6. A round bottom flask equipped with a stir bar was charged with compound **6** (0.106 g, 0.338 mmol), DOX.HCl (0.15 g, 0.26 mmol), and anhydrous DMF (3 mL). The mixture was

cooled to 0 °C in an ice bath before adding N,N-diisopropylethylamine (DIPEA, 0.087 g, 0.676 mmol) dropwise. After overnight reaction at room temperature, the mixture was extracted with ether (100 mL). The organic fraction was washed with 10% NH_4Cl (50 mL), water (50 mL), and brine (50 mL). The organic phase was dried over Na_2SO_4, filtered, and concentrated under reduced pressure. The product was separated by column chromatography (2-10 % MeOH/DCM) to provide the title compound as a red solid (0.12 g, 65%). R_f = 0.5 (MeOH/DCM 10 : 1). ^{1}H NMR (400 MHz, $CDCl_3$) δ 8.03 (dd, J = 7.7, 1.0 Hz, 1H), 7.78 (t, J = 8.1 Hz, 1H), 7.39 (d, J = 8.4 Hz, 1H), 6.82 (s, 2H), 5.49 (d, J = 3.9 Hz, 1H), 5.28 (dd, J = 4.1, 2.1 Hz, 1H), 5.06 (d, J = 20.9 Hz, 3H), 4.76 (s, 2H), 4.53 (s, 1H), 4.08 (s, 4H), 3.88 (d, J = 14.2 Hz, 1H), 3.66 (s, 1H), 3.27 (d, J = 18.9 Hz, 1H), 3.06 – 2.83 (m, 2H), 2.43 – 2.08 (m, 11H), 1.80 – 1.57 (m, 2H), 1.28 (d, J = 6.5 Hz, 3H). ^{13}C NMR (100 MHz, $CDCl_3$) δ 213.99, 187.23, 186.82, 161.20, 156.34, 155.94, 155.79, 138.51, 138.24, 135.92, 135.64, 133.71, 129.40, 129.17, 121.00, 120.00, 118.59, 111.72, 111.55, 100.96, 69.91, 69.73, 67.41, 65.69, 61.51, 56.82, 47.06, 35.78, 34.13, 30.36, 21.10, 19.61, 16.98. HRMS (DART/Orbitrap) m/z: Calcd for $C_{38}H_{40}NO_{13}$[M-H]$^-$, 718.2505; Found 718.2510.

Scheme S9. Synthesis Route of **ProR848**.

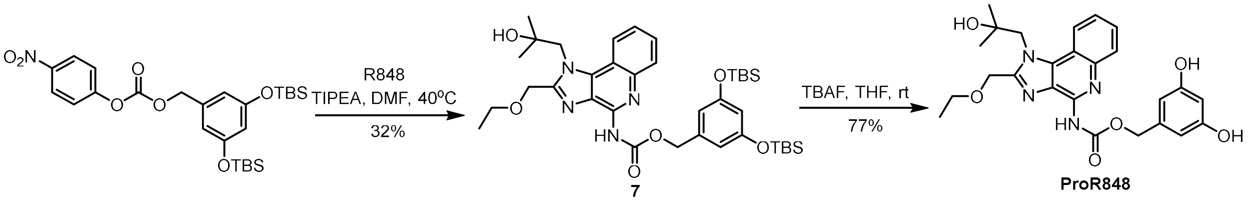

Compound 7. A round bottom flask equipped with a stir bar was charged with compound **2** (0.22 g, 0.412 mmol), R848 (0.1 g, 0.318 mmol), and anhydrous DMF (2 mL). The mixture was cooled to 0 °C in an ice bath before adding N,N-diisopropylethylamine (DIPEA, 0.089 g, 0.689 mmol) dropwise. After overnight reaction at 40 °C, the mixture was extracted with ether (200 mL). The organic fraction was washed with 10% NH_4Cl (50 mL), water (50 mL), and brine (50 mL). The organic phase was dried over Na_2SO_4, filtered, and concentrated under reduced pressure. The product was separated by column chromatography (1-5 % MeOH/$CHCl_3$) to provide the title compound as a red solid (0.072 g, 32%). R_f = 0.5 (MeOH/DCM 20 : 1). ^{1}H NMR (400 MHz, $CDCl_3$) δ 8.25 – 8.04 (m, 2H), 7.59 (ddd, J = 8.4, 6.9, 1.3 Hz, 1H), 7.46 (ddd, J = 8.4, 7.0, 1.4 Hz, 1H), 6.54 (dd, J = 18.1, 2.2 Hz, 2H), 6.31 (dt, J = 6.8, 2.1 Hz, 1H), 5.33 – 5.09 (m, 3H), 4.83 (d, J = 44.9 Hz, 4H), 3.64 (q, J = 7.0 Hz, 2H), 3.20 (s, 1H), 1.44 – 1.13 (m, 12H), 0.97 (d, J = 2.7 Hz, 21H), 0.20 (d, J = 2.4 Hz, 13H). ^{13}C NMR (100 MHz, $CDCl_3$) δ 156.88, 156.77, 154.41, 150.74, 137.96, 136.74, 135.55, 127.68, 126.39, 124.60, 122.60, 119.97, 116.76, 113.75, 112.37, 111.84, 71.60, 67.16, 66.76, 65.04, 56.57, 27.42, 25.80, 18.34, 15.03, -4.22. HRMS (DART/Orbitrap) m/z: Calcd for $C_{37}H_{55}N_4O_6Si_2$[M-H]$^-$, 707.3666; Found 707.3671.

ProR848. A flame-dried round bottom flask equipped with a stir bar was charged with **7** (10.5 mg, 0.0148 mmol) and 0.5 mL anhydrous THF. The mixture was cooled to 0 °C in an ice bath, followed by the dropwise addition of TBAF (1 M in THF, 0.033 mL, 0.033 mmol). The stirred reaction was allowed to warm up to room temperature. After one hour, the reaction was quenched with NH_4Cl

(5 mL) and extracted with $CHCl_3$ (10 mL). The organic fraction was washed with water (5 mL) and brine (5 mL). The organic fraction was dried over Na_2SO_4, filtered, and concentrated under reduced pressure. The crude product was purified by column chromatography (1–15% MeOH/DCM) to yield the title compound as a white solid (5.5 mg, 77%). R_f = 0.3 ($CHCl_3$: MeOH 10 : 1). ^{1}H NMR (400 MHz, MeOD) δ 8.38 (d, J = 8.3 Hz, 1H), 8.08 (d, J = 8.4 Hz, 1H), 7.60 (t, J = 7.7 Hz, 1H), 7.51 (t, J = 7.7 Hz, 1H), 6.43 (d, J = 2.2 Hz, 2H), 6.24 (t, J = 2.3 Hz, 1H), 5.16 (s, 2H), 3.65 – 3.50 (m, 2H), 3.35 (s, 2H), 1.34 – 0.93 (m, 11H). ^{13}C NMR (100 MHz, MeOD) ^{13}C NMR (100 MHz, Methanol-d4) δ 159.32, 154.33, 152.76, 145.32, 143.64, 141.27, 138.48, 136.17, 128.55, 127.39, 124.61, 121.02, 116.86, 105.93, 101.93, 71.13, 66.69, 66.29, 65.05, 55.28, 26.51, 14.01. HRMS (DART/Orbitrap) m/z: Calcd for $C_{25}H_{27}N_4O_6[M-H]^-$, 479.1936; Found 479.1939.

5. NMR Spectra

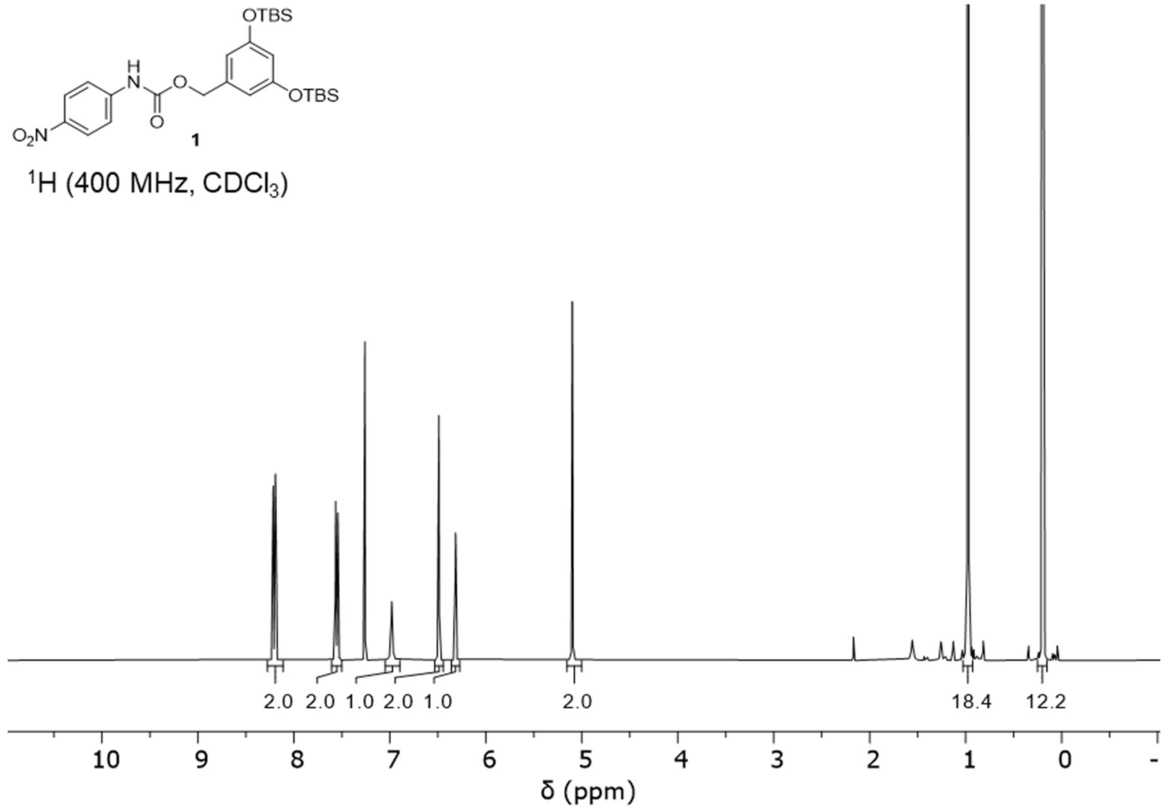

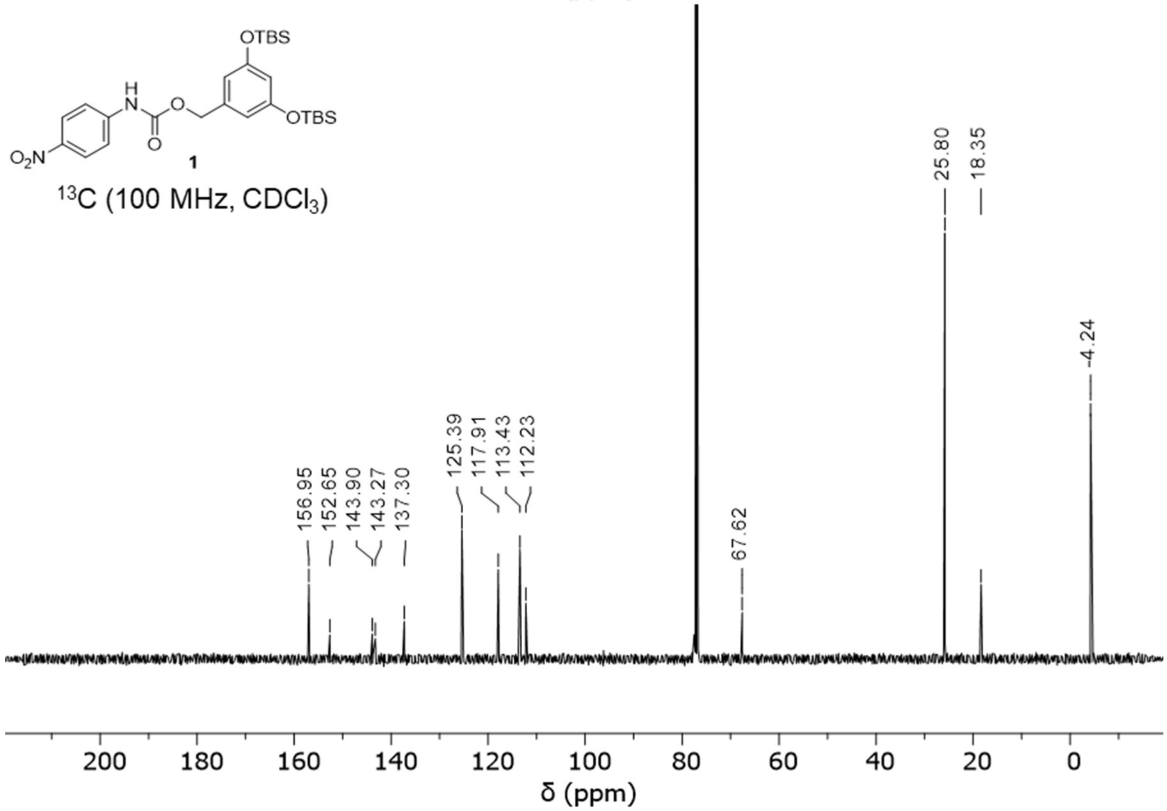

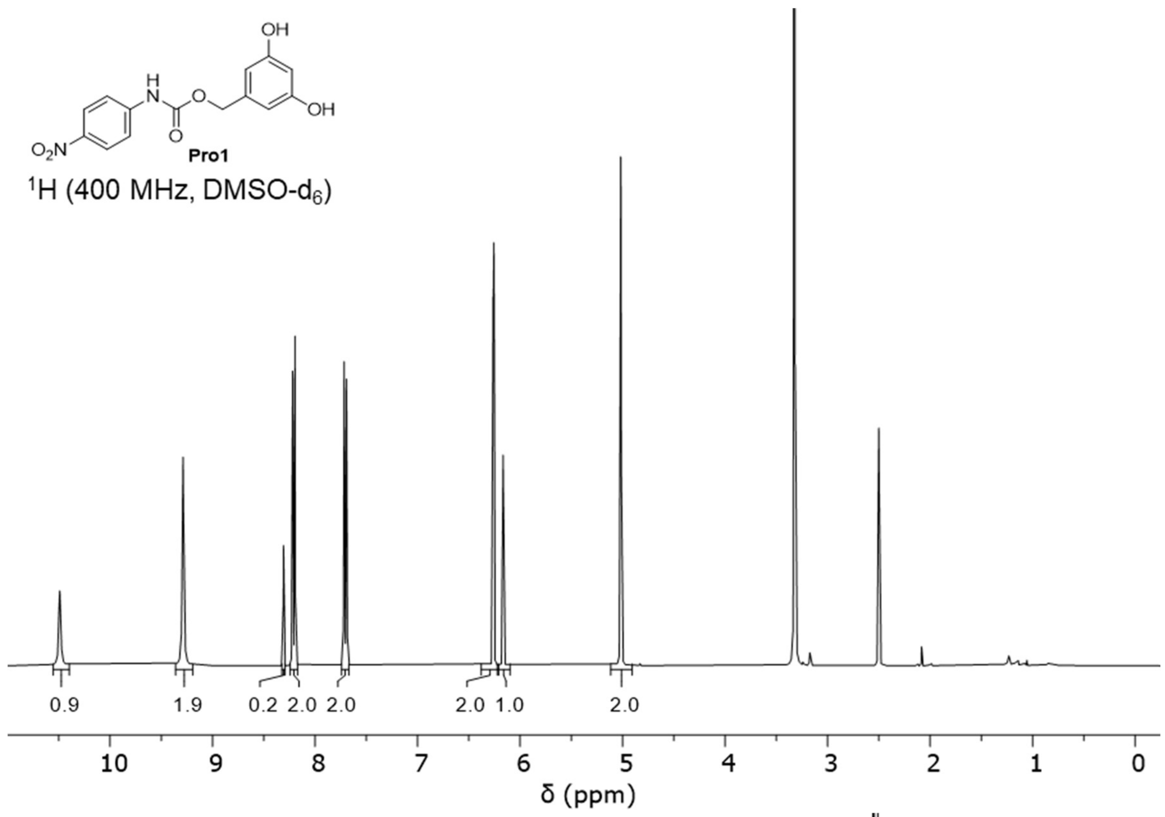
OH
H
N
O
O
OH
O₂N
Pro1
¹H (400 MHz, DMSO-d₆)
0.9
1.9
0.2 2.0 2.0
2.0 1.0
2.0
10 9 8 7 6 5 4 3 2 1 0
δ (ppm)

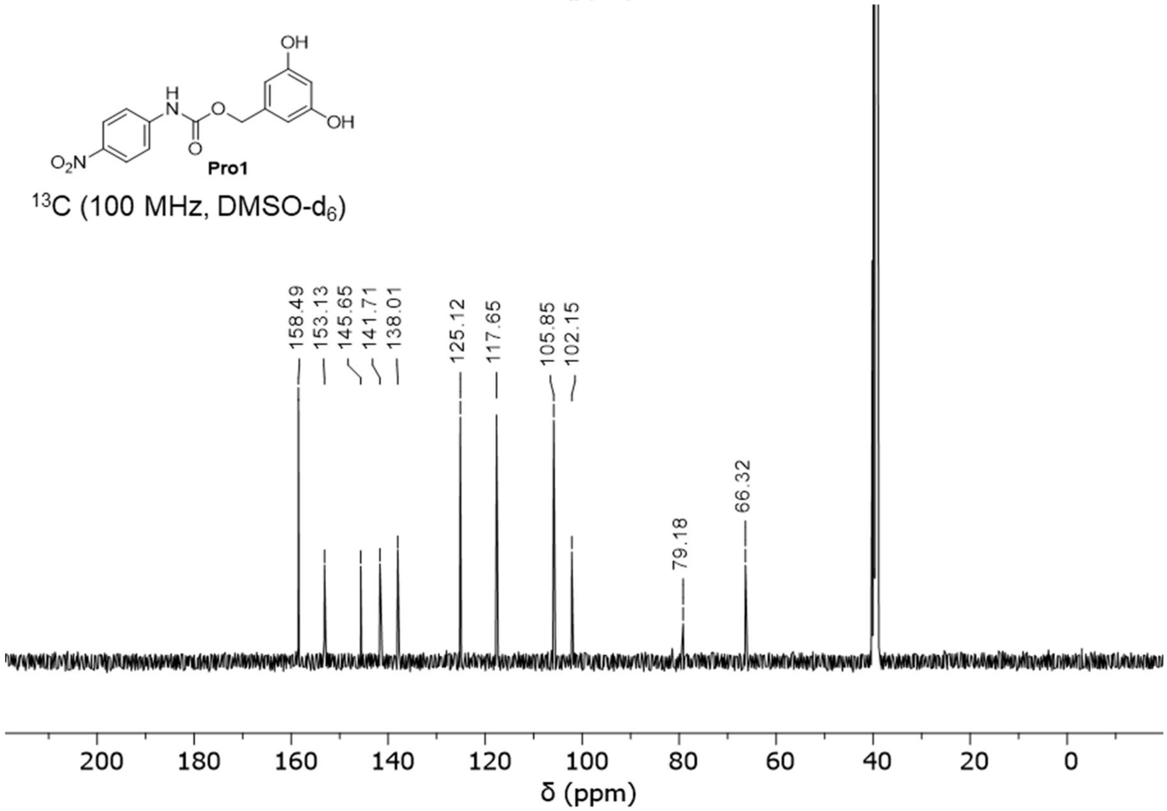
OH
H
N
O
O
OH
O₂N
Pro1
¹³C (100 MHz, DMSO-d₆)
158.49
153.13
145.65
141.71
138.01
125.12
117.65
105.85
102.15
79.18
66.32
200 180 160 140 120 100 80 60 40 20 0
δ (ppm)

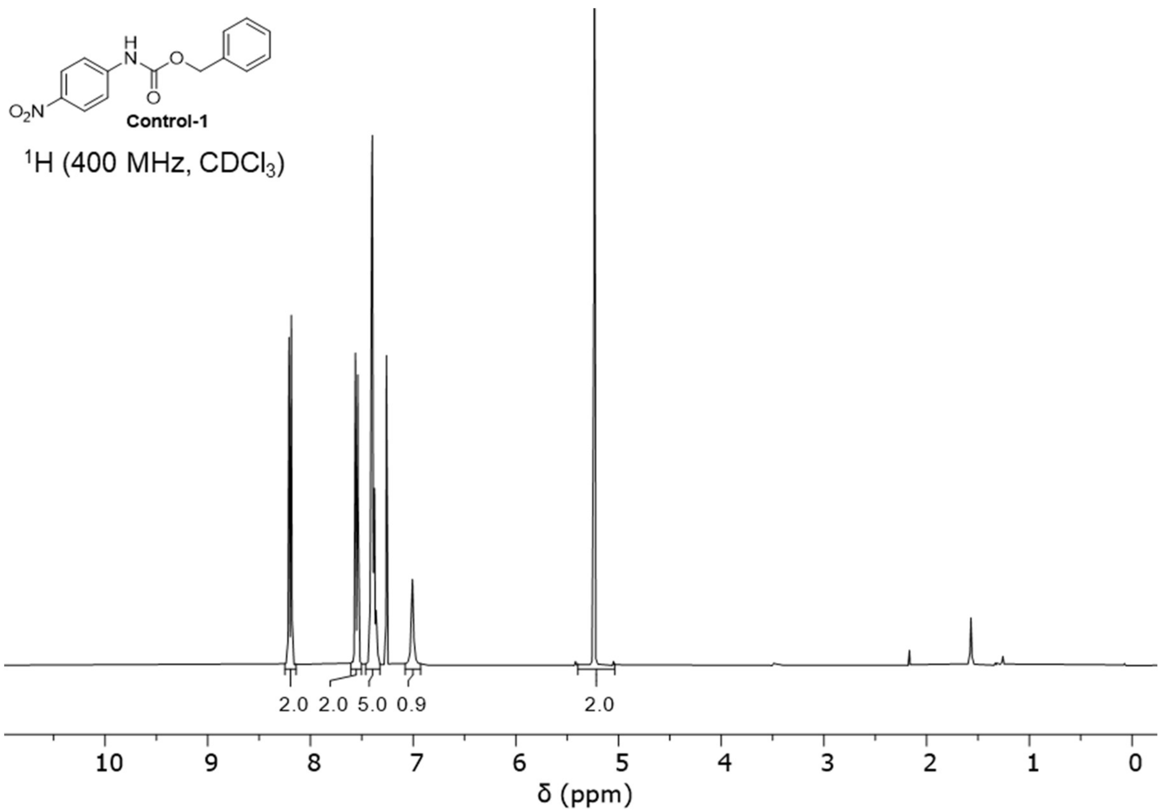
Control-1
^{1}H (400 MHz, CDCl$_3$)
2.0 2.0 5.0 0.9 2.0
δ (ppm)

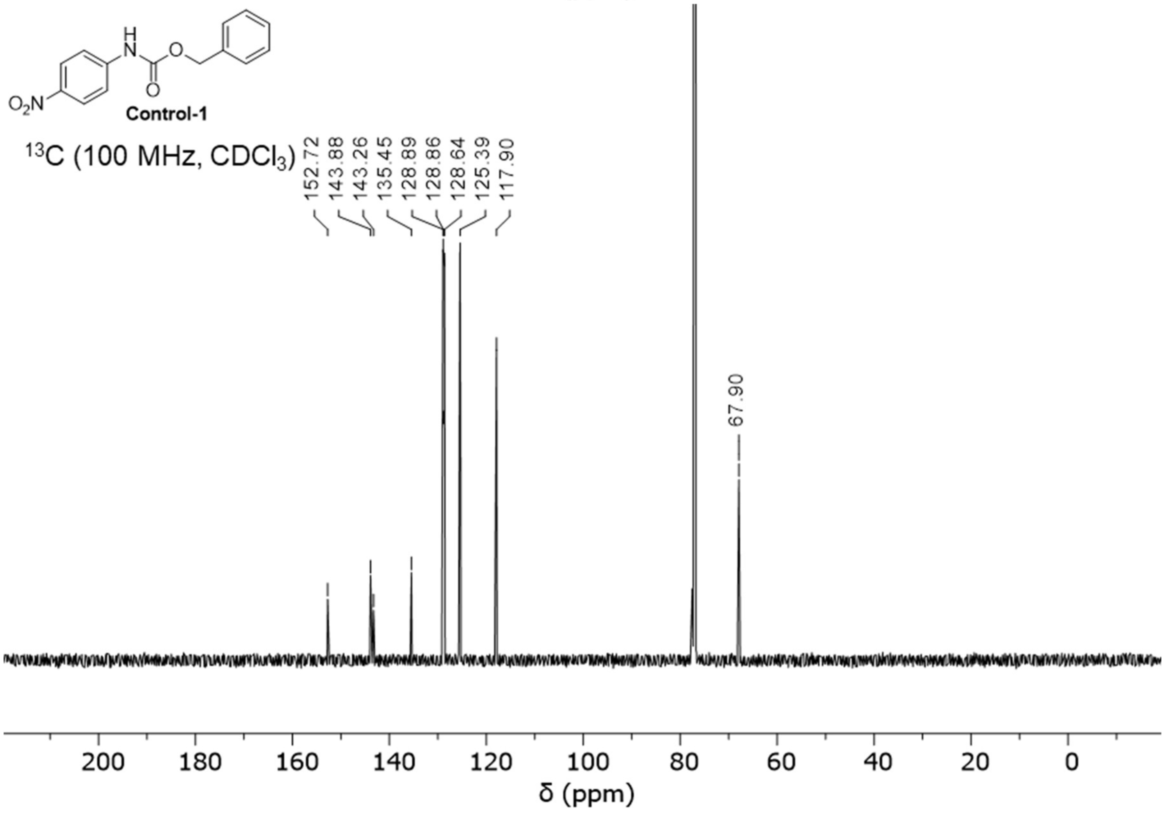
Control-1
^{13}C (100 MHz, CDCl$_3$)
152.72 143.88 143.26 135.45 128.89 128.86 128.64 125.39 117.90
67.90
δ (ppm)

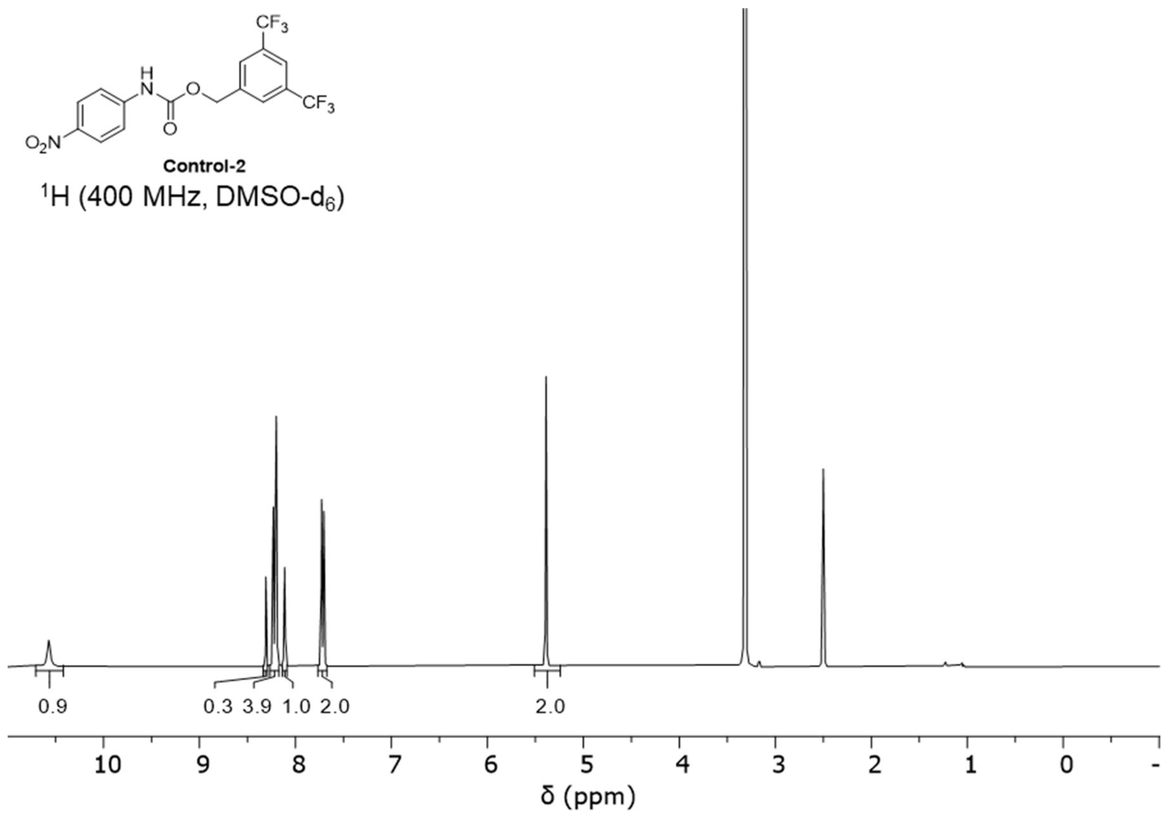
CF3
H
N
O
O
CF3
O2N
Control-2
1H (400 MHz, DMSO-d6)
0.9
0.3 3.9 1.0 2.0
2.0
10
9
8
7
6
5
4
3
2
1
0
δ (ppm)

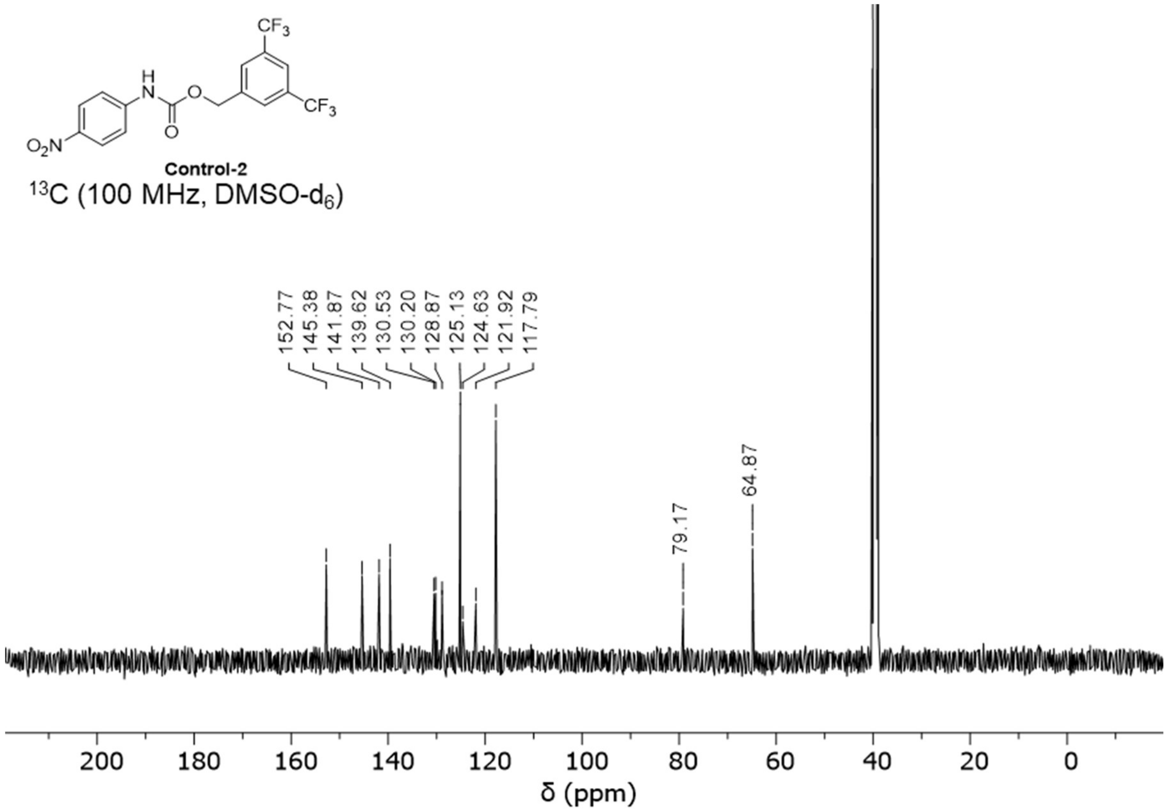
CF3
H
N
O
O
CF3
O2N
Control-2
13C (100 MHz, DMSO-d6)
152.77
145.38
141.87
139.62
130.53
130.20
128.87
125.13
124.63
121.92
117.79
79.17
64.87
200
180
160
140
120
100
80
60
40
20
0
δ (ppm)

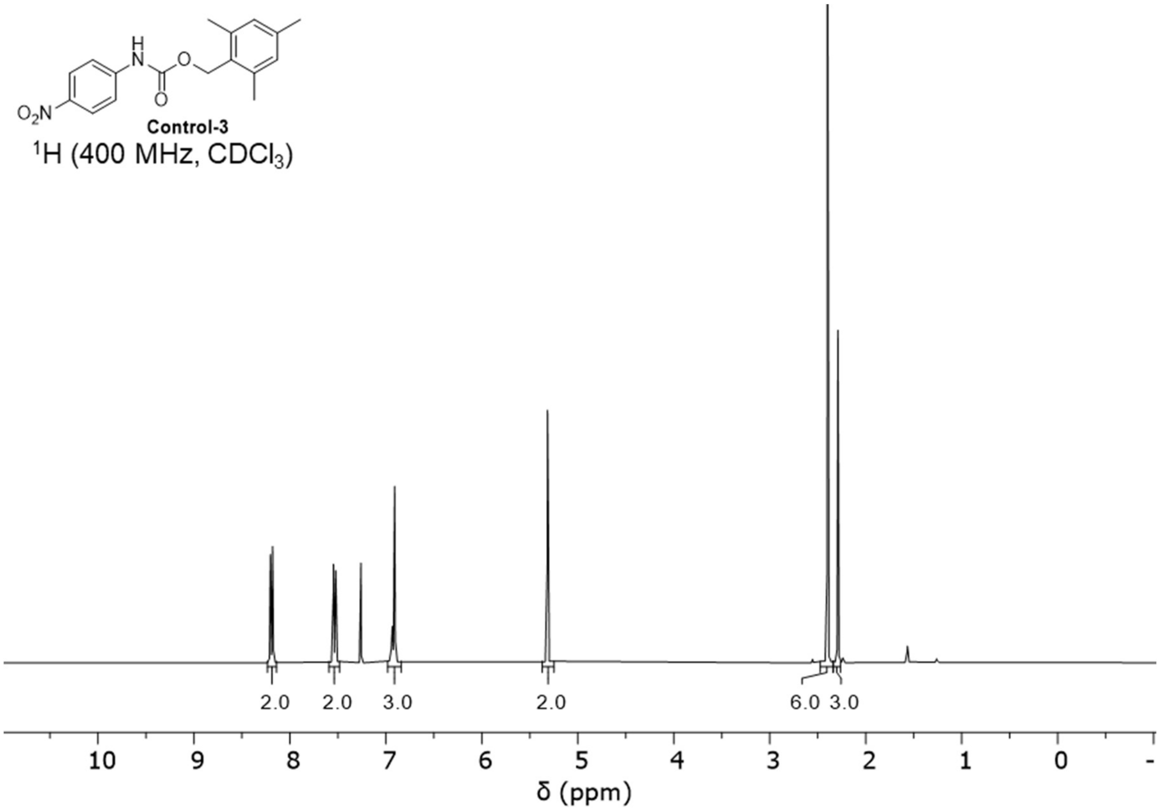
Control-3
^{1}H (400 MHz, CDCl$_3$)
2.0
2.0
3.0
2.0
6.0
3.0
10 9 8 7 6 5 4 3 2 1 0
δ (ppm)

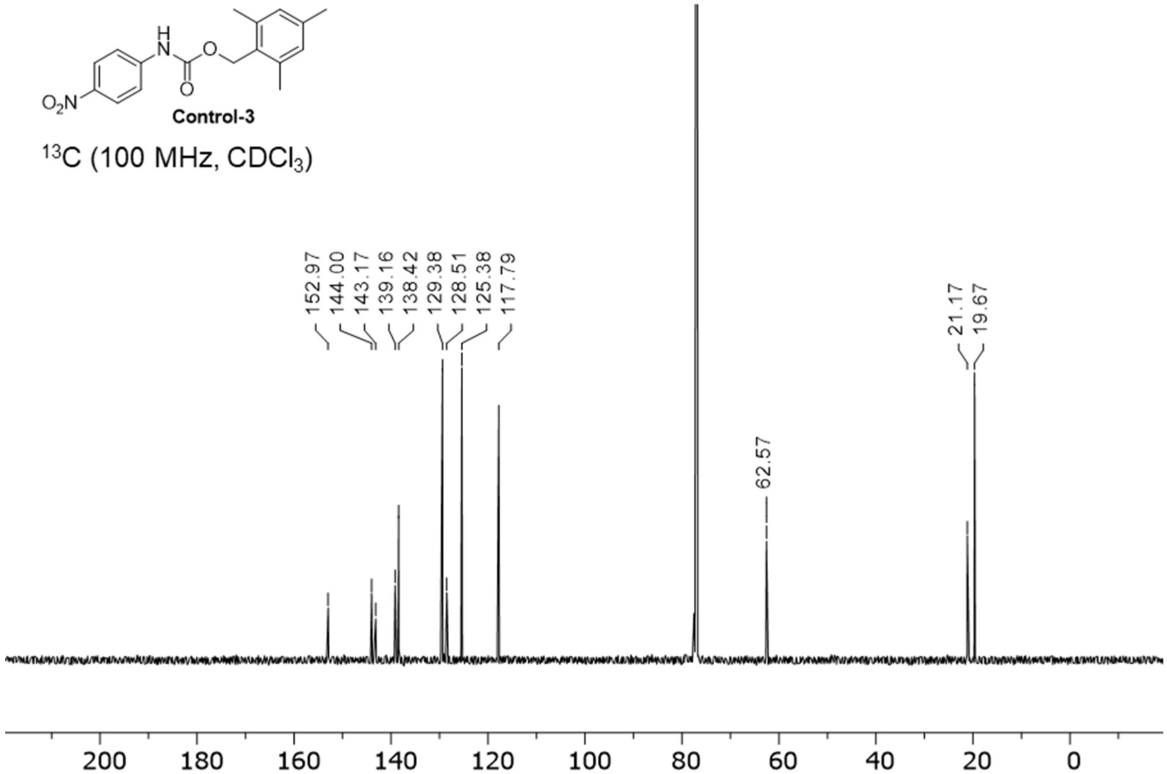
Control-3
^{13}C (100 MHz, CDCl$_3$)
152.97
144.00
143.17
139.16
138.42
129.38
128.51
125.38
117.79
62.57
21.17
19.67
200 180 160 140 120 100 80 60 40 20 0
δ (ppm)

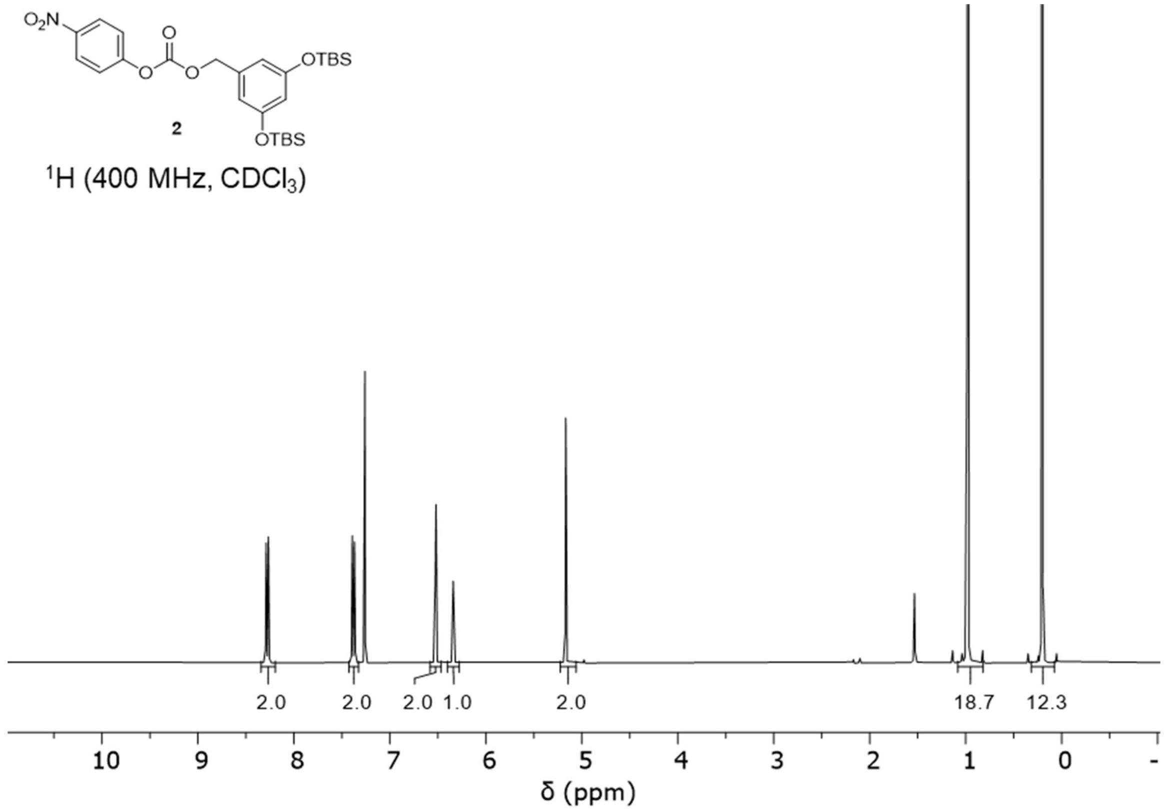

2
OTBS
OTBS
^{1}H (400 MHz, $CDCl_3$)
2.0
2.0
2.0
1.0
2.0
18.7
12.3
10
9
8
7
6
5
4
3
2
1
0
δ (ppm)

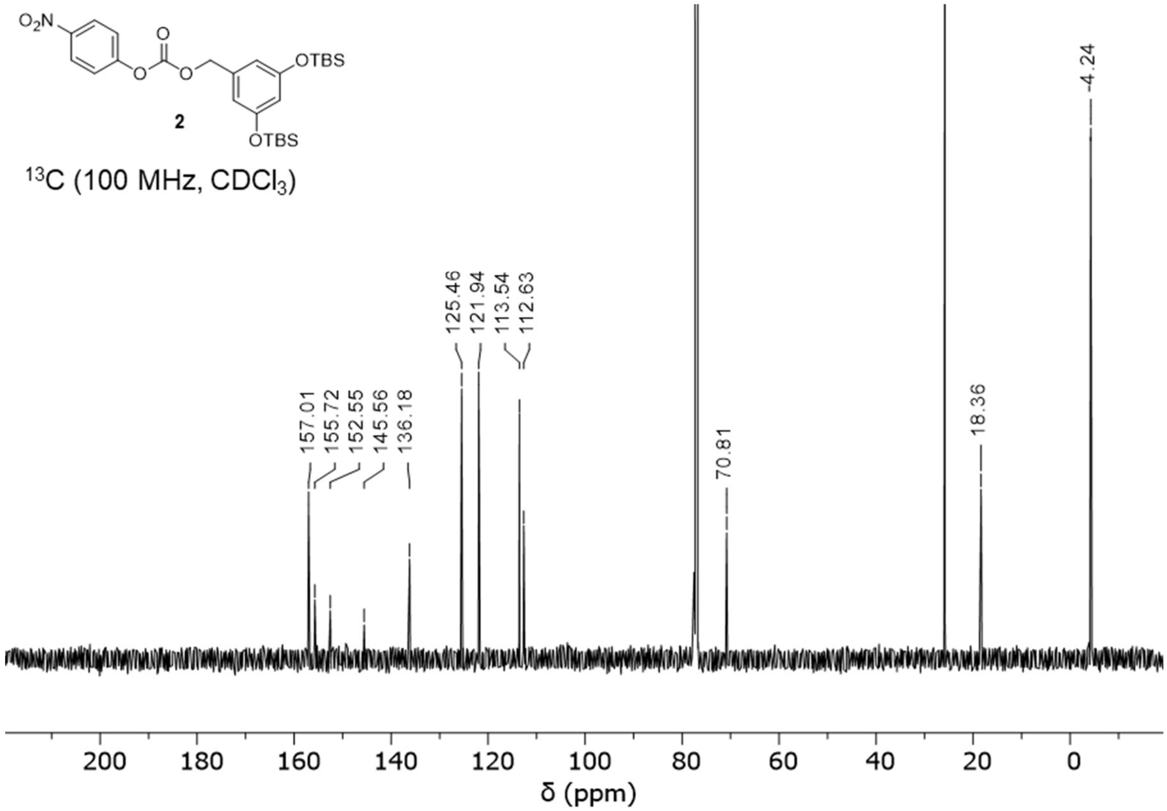

2
OTBS
OTBS
^{13}C (100 MHz, $CDCl_3$)
157.01
155.72
152.55
145.56
136.18
125.46
121.94
113.54
112.63
70.81
18.36
-4.24
200
180
160
140
120
100
80
60
40
20
0
δ (ppm)

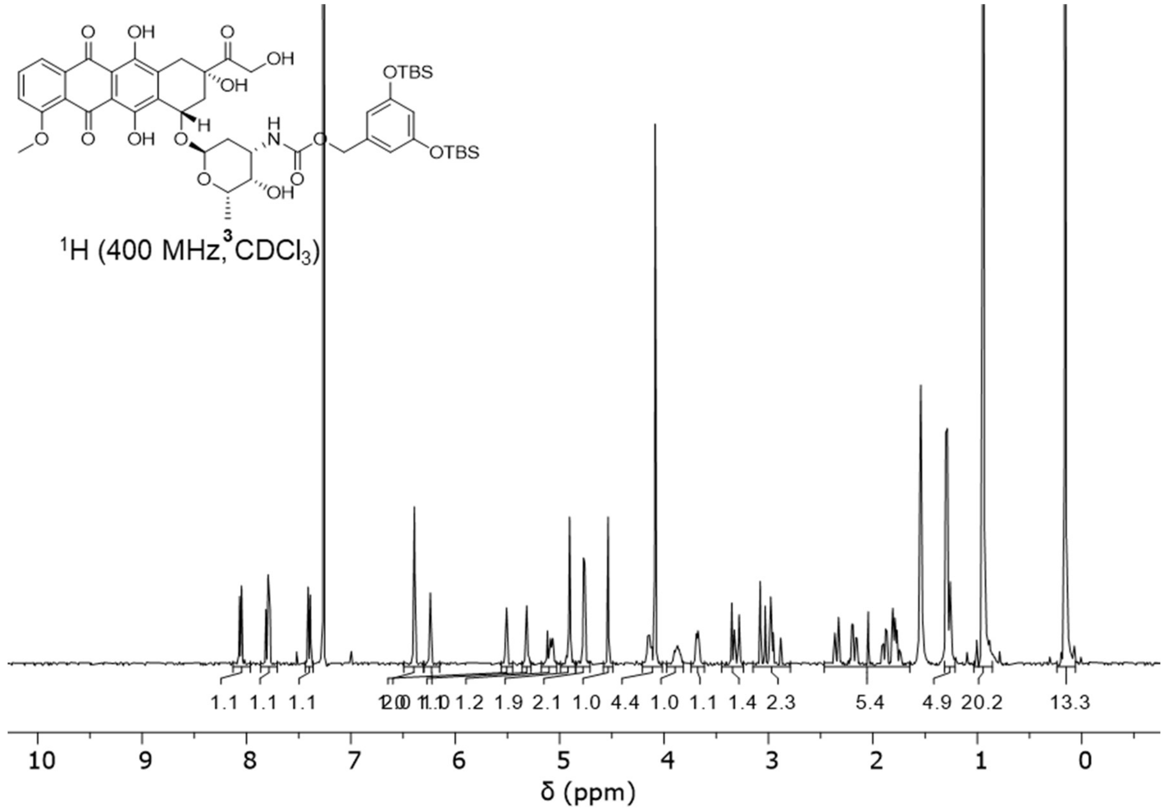
OH
O
OH
OTBS
OH
H
N
O
O
OTBS
OH
O
OH
O
OH
3
^{1}H (400 MHz, CDCl$_3$)
1.1
1.1
1.1
1.0
1.1
1.0
1.1
1.2
1.9
2.1
1.0
4.4
1.0
1.1
1.4
2.3
5.4
4.9
20.2
13.3
10
9
8
7
6
5
4
3
2
1
0
δ (ppm)

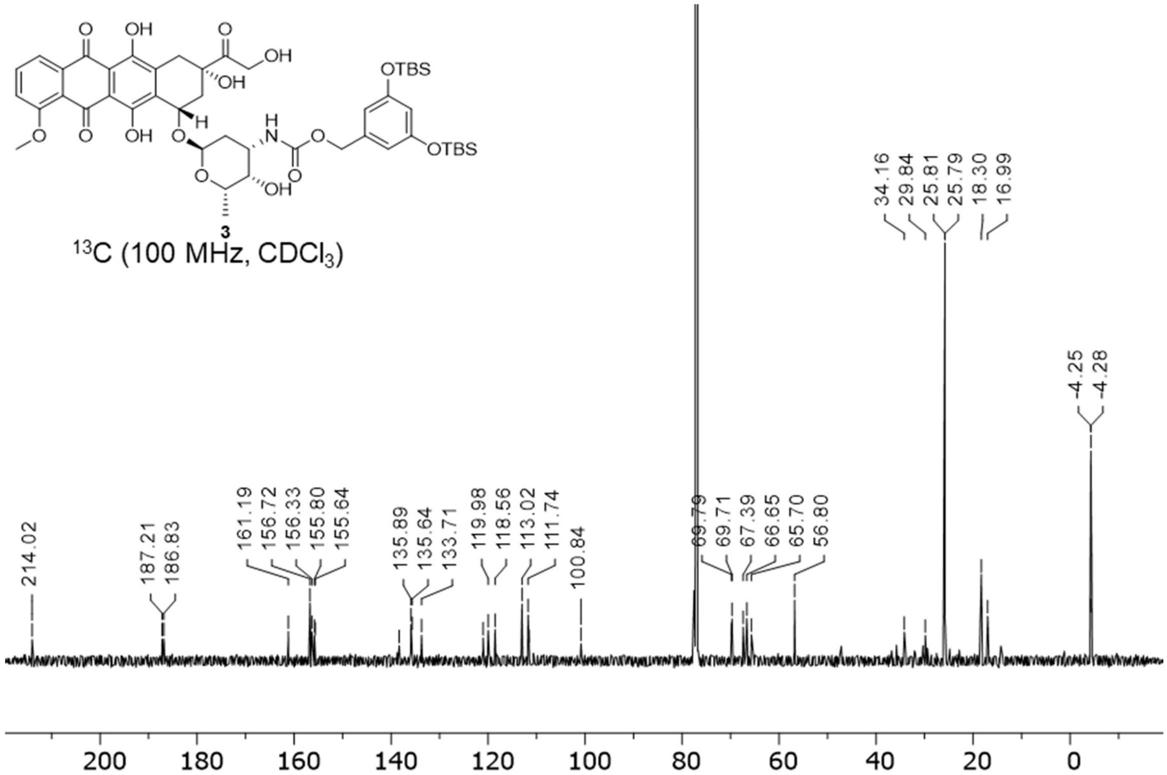
OH
OTBS
H
N
OTBS
3
^{13}C (100 MHz, CDCl$_3$)
34.16
29.84
25.81
25.79
18.30
16.99
-4.25
-4.28
214.02
187.21
186.83
161.19
156.72
156.33
155.80
155.64
135.89
135.64
133.71
119.98
118.56
113.02
111.74
100.84
69.79
69.71
67.39
66.65
65.70
56.80
200
180
160
140
120
100
80
60
40
20
0
δ (ppm)

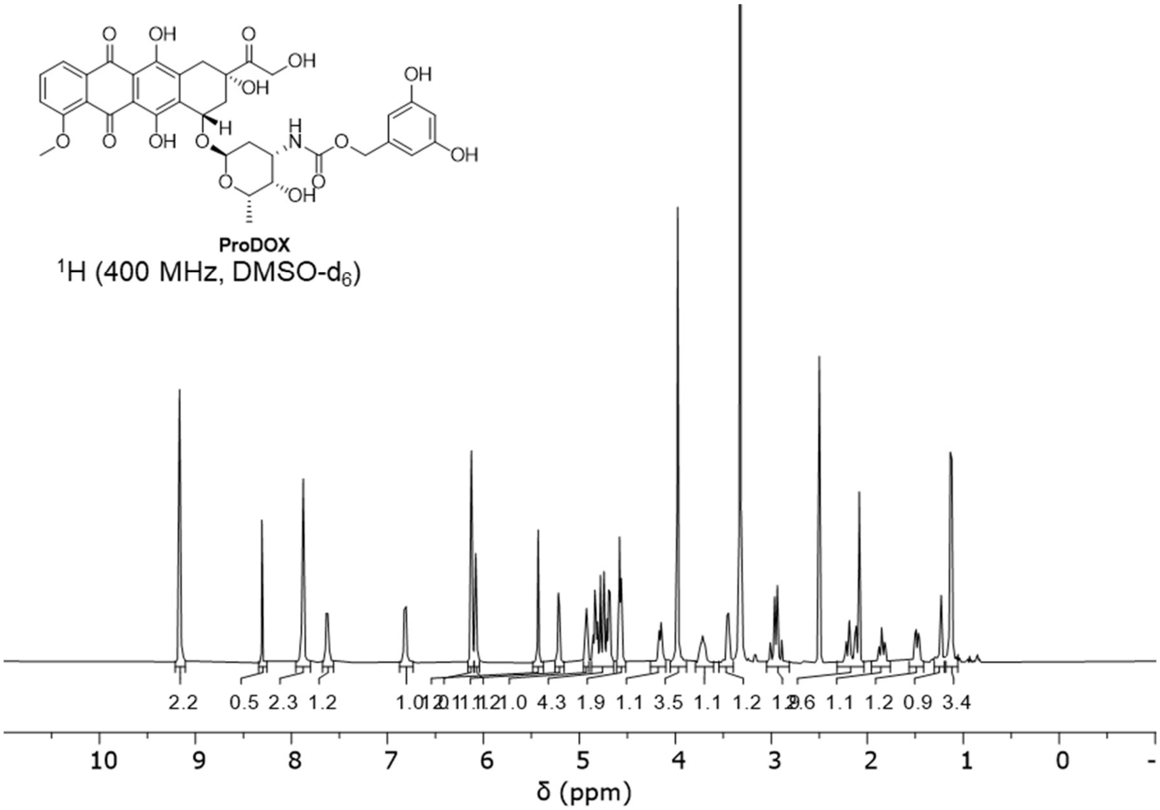
ProDOX
^{1}H (400 MHz, DMSO-d$_6$)
δ (ppm)

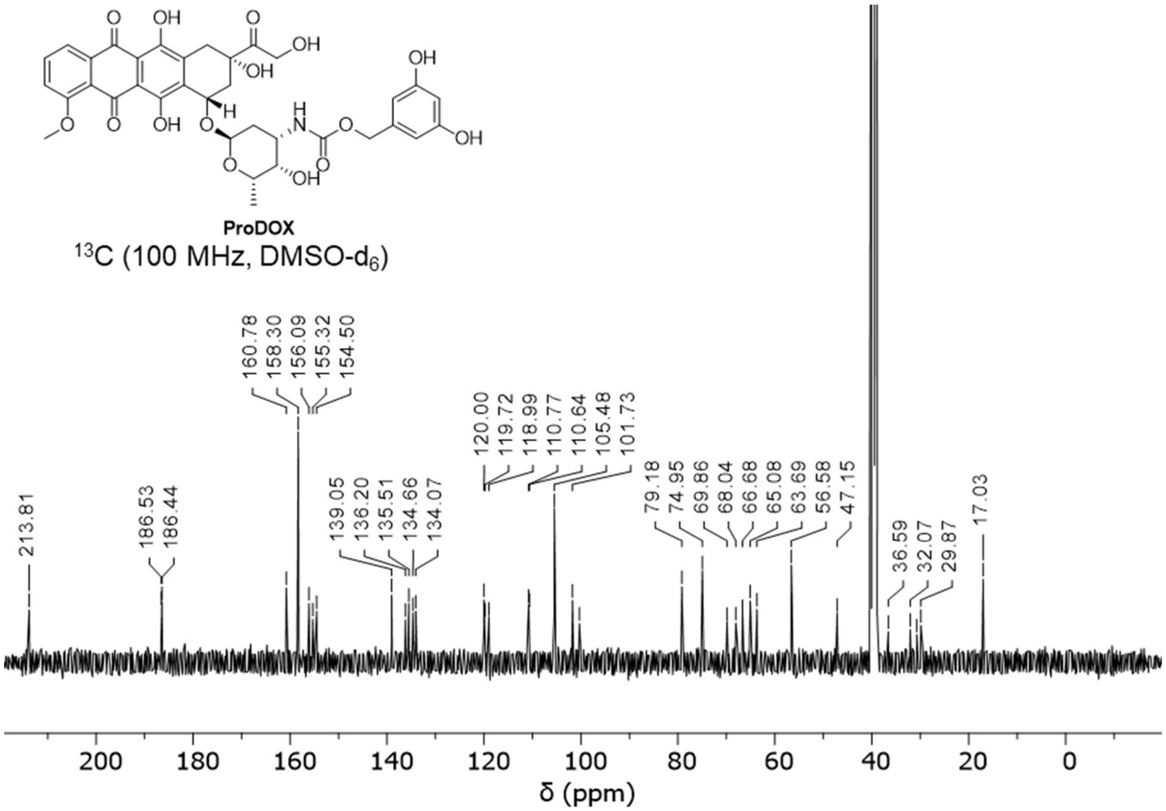
ProDOX
^{13}C (100 MHz, DMSO-d$_6$)
213.81
186.53
186.44
160.78
158.30
156.09
155.32
154.50
139.05
136.20
135.51
134.66
134.07
120.00
119.72
118.99
110.77
110.64
105.48
101.73
79.18
74.95
69.86
68.04
66.68
65.08
63.69
56.58
47.15
36.59
32.07
29.87
17.03
δ (ppm)

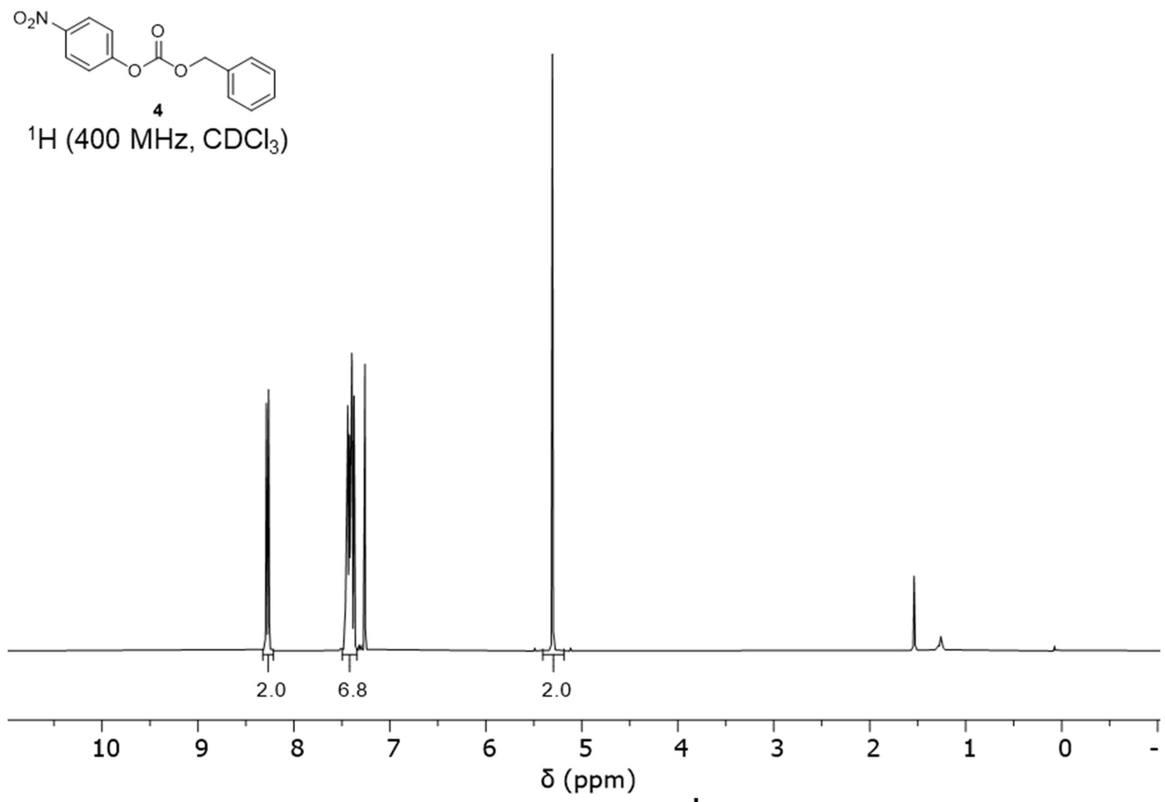

O_2N
O
O
O
4
^1H (400 MHz, $CDCl_3$)
2.0
6.8
2.0
10
9
8
7
6
5
4
3
2
1
0
δ (ppm)

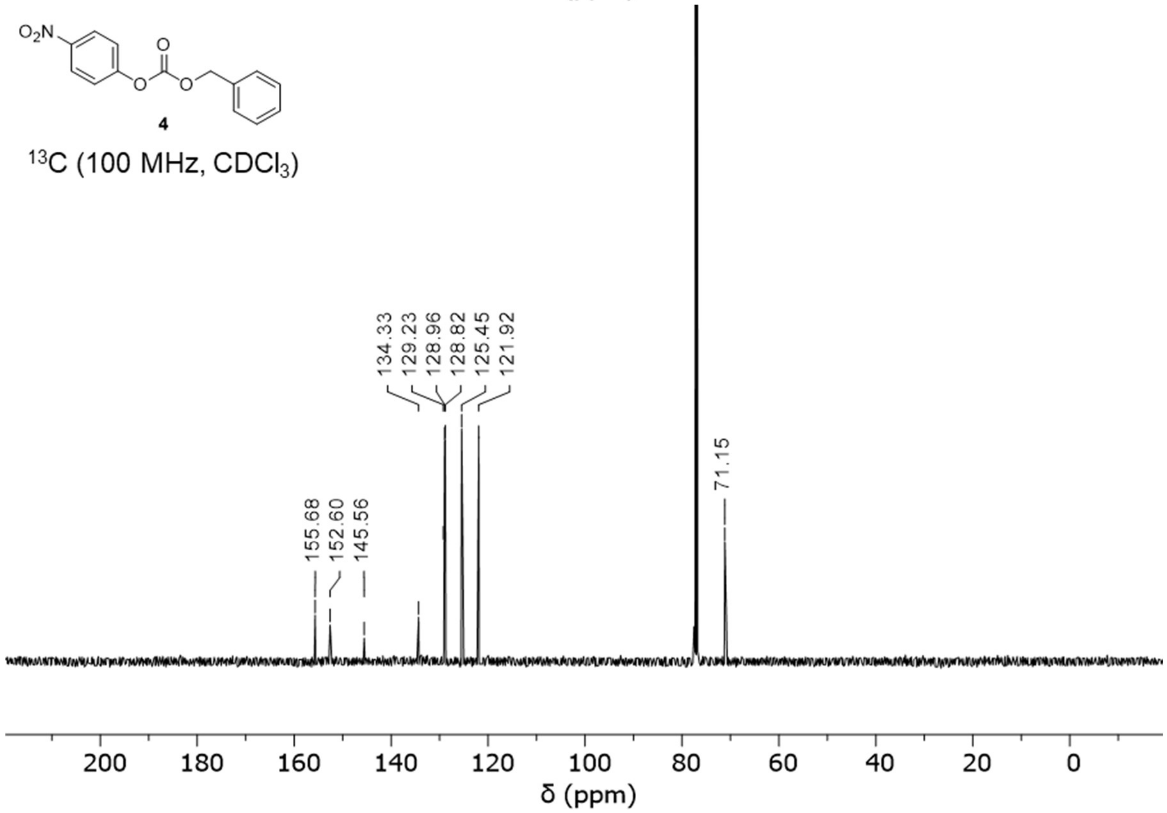

O_2N
O
O
O
4
^{13}C (100 MHz, $CDCl_3$)
134.33
129.23
128.96
128.82
125.45
121.92
71.15
155.68
152.60
145.56
200
180
160
140
120
100
80
60
40
20
0
δ (ppm)

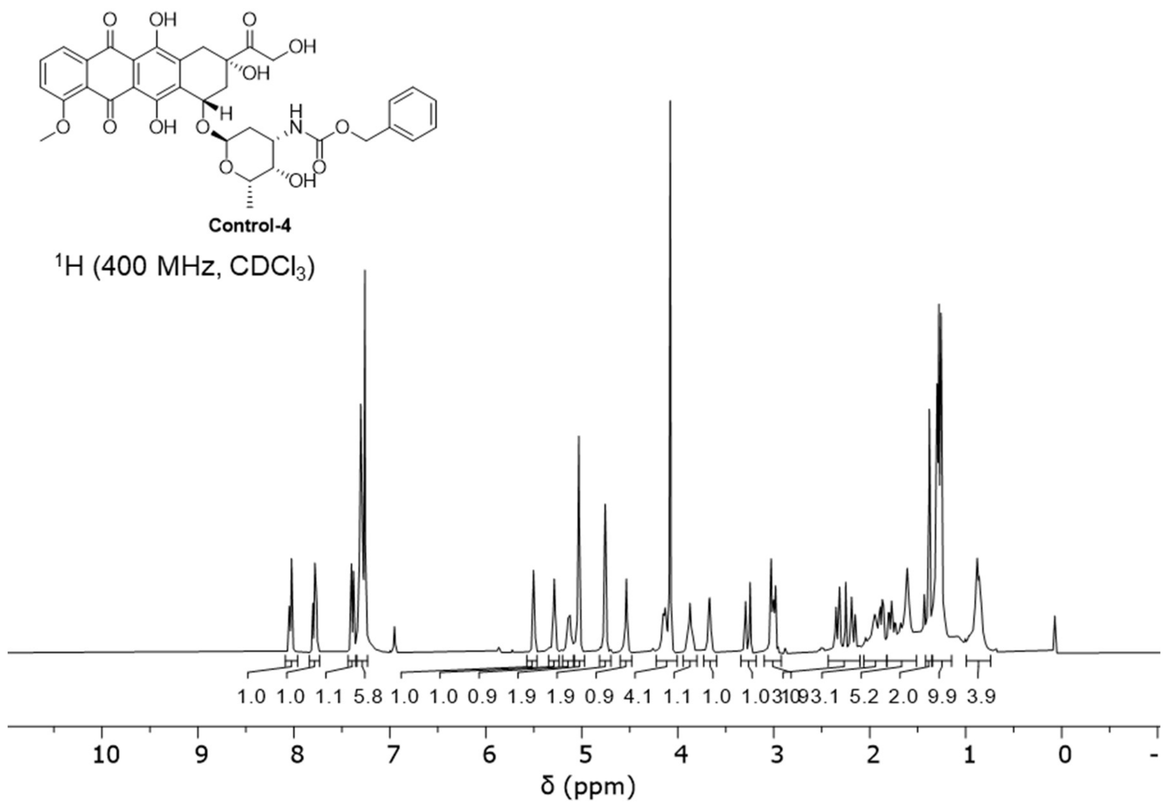

Control-4
^{1}H (400 MHz, CDCl$_3$)
1.0 1.0 1.1 5.8 1.0 1.0 0.9 1.9 1.9 0.9 4.1 1.1 1.0 1.03.10 93.1 5.2 2.0 9.9 3.9
δ (ppm)

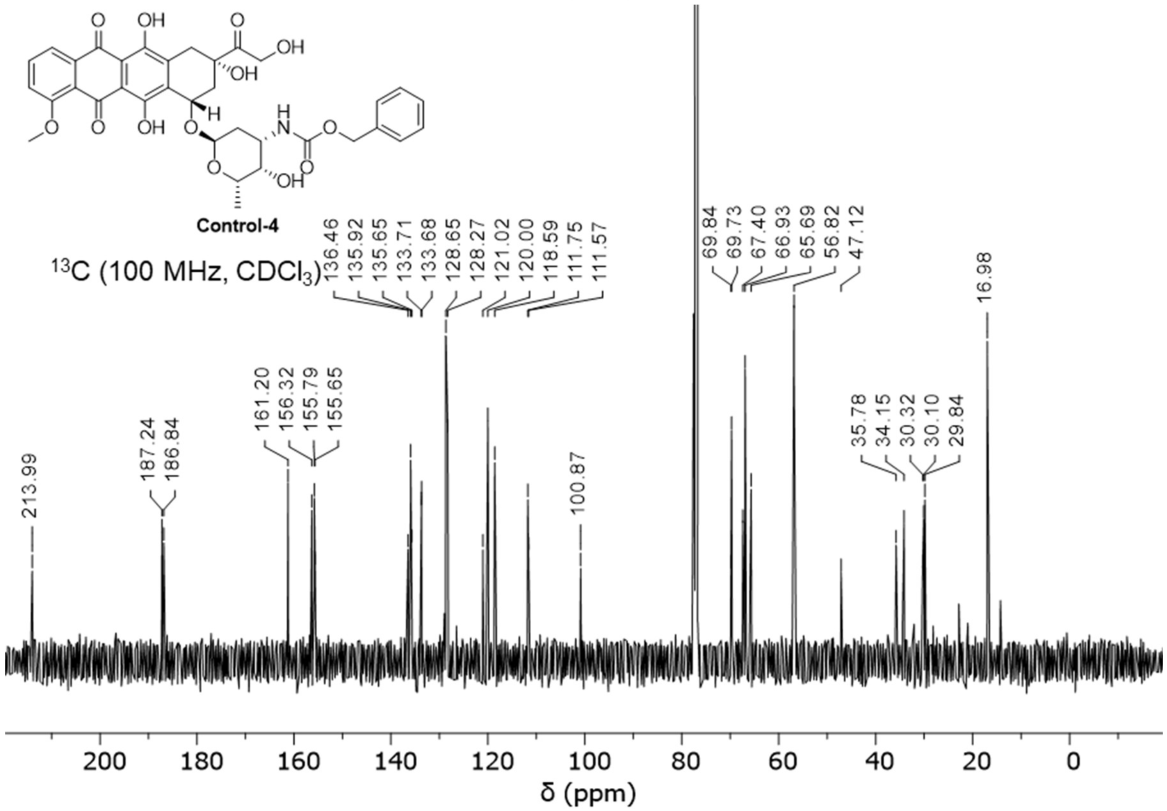

Control-4
^{13}C (100 MHz, CDCl$_3$)
136.46 135.92 135.65 133.71 133.68 128.65 128.27 121.02 120.00 118.59 111.75 111.57
69.84 69.73 67.40 66.93 65.69 56.82 47.12
16.98
161.20 156.32 155.79 155.65
187.24 186.84
213.99
100.87
35.78 34.15 30.32 30.10 29.84
δ (ppm)

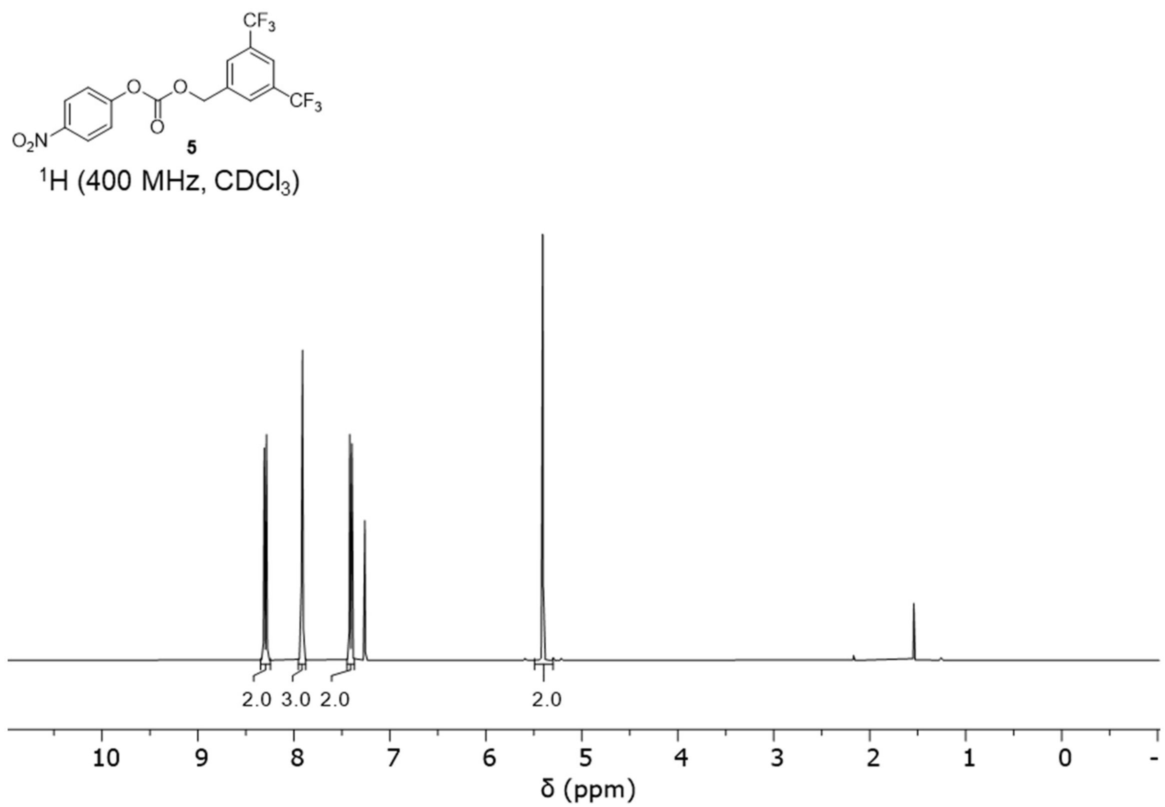
CF3
CF3
O2N
5
1H (400 MHz, CDCl3)
2.0 3.0 2.0
2.0
10 9 8 7 6 5 4 3 2 1 0
δ (ppm)

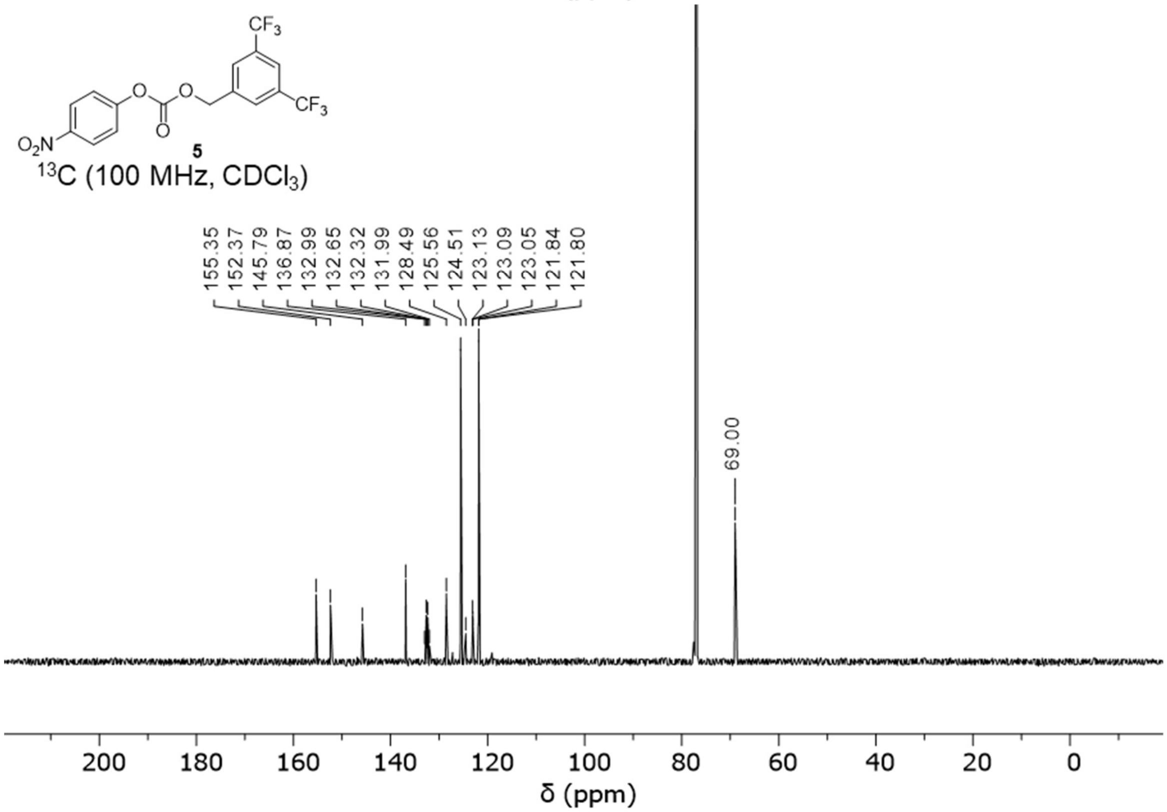
CF3
CF3
O2N
5
13C (100 MHz, CDCl3)
155.35
152.37
145.79
136.87
132.99
132.65
132.32
131.99
128.49
125.56
124.51
123.13
123.09
123.05
121.84
121.80
69.00
200 180 160 140 120 100 80 60 40 20 0
δ (ppm)

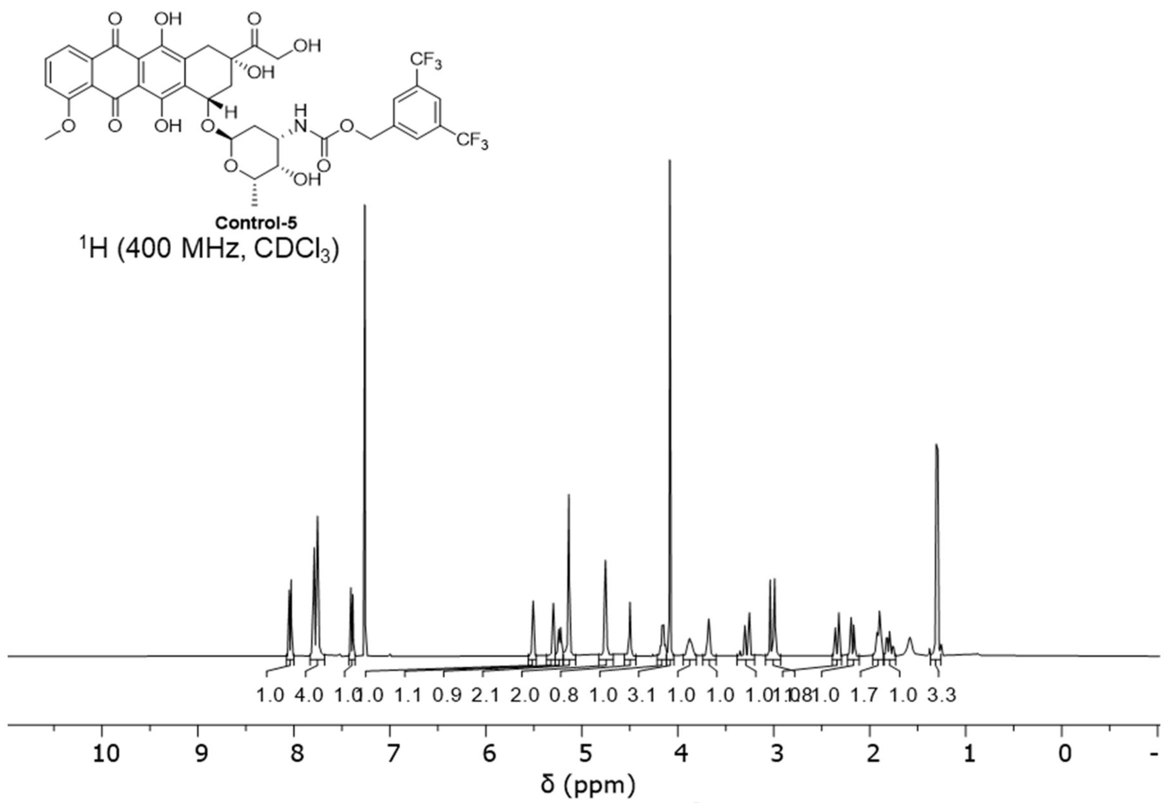
Control-5
^{1}H (400 MHz, CDCl$_3$)
1.0 4.0 1.0 1.0 1.1 0.9 2.1 2.0 0.8 1.0 3.1 1.0 1.0 1.0 1.0 0.8 1.0 1.7 1.0 3.3
10 9 8 7 6 5 4 3 2 1 0
δ (ppm)

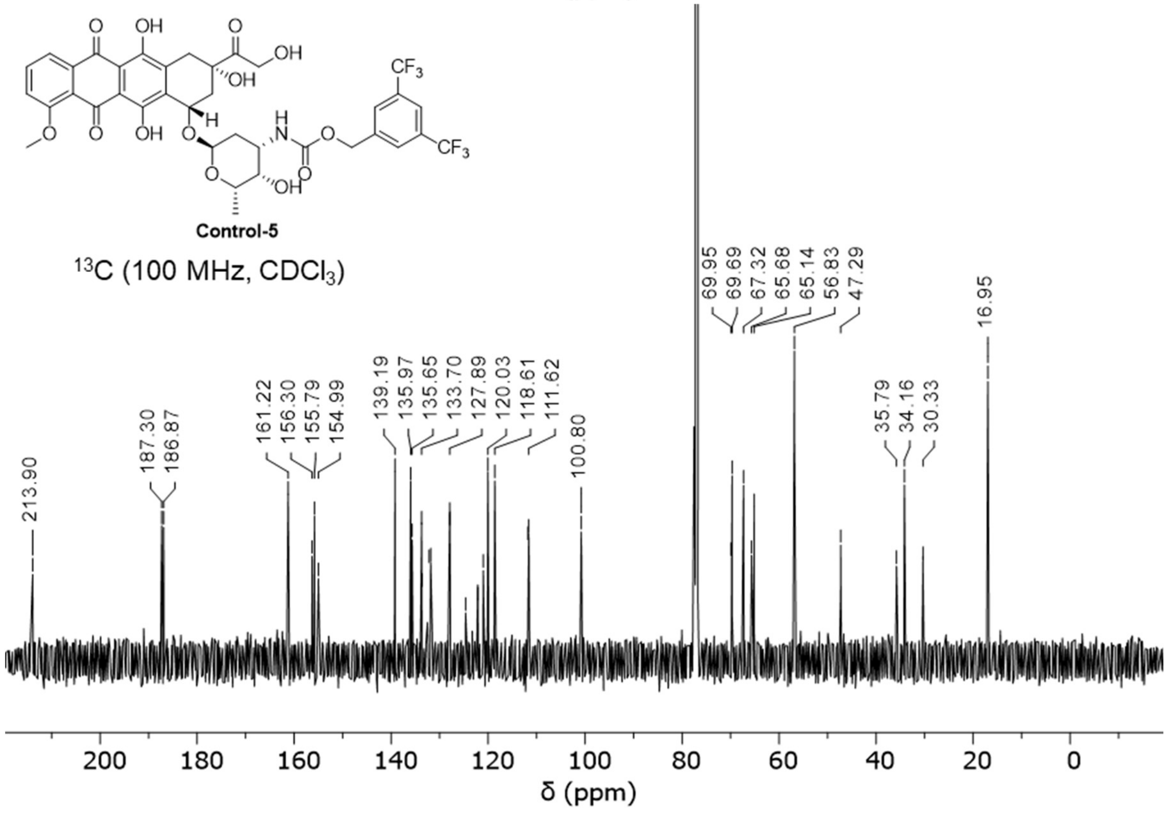
Control-5
^{13}C (100 MHz, CDCl$_3$)
213.90
187.30
186.87
161.22
156.30
155.79
154.99
139.19
135.97
135.65
133.70
127.89
120.03
118.61
111.62
100.80
69.95
69.69
67.32
65.68
65.14
56.83
47.29
35.79
34.16
30.33
16.95
200 180 160 140 120 100 80 60 40 20 0
δ (ppm)

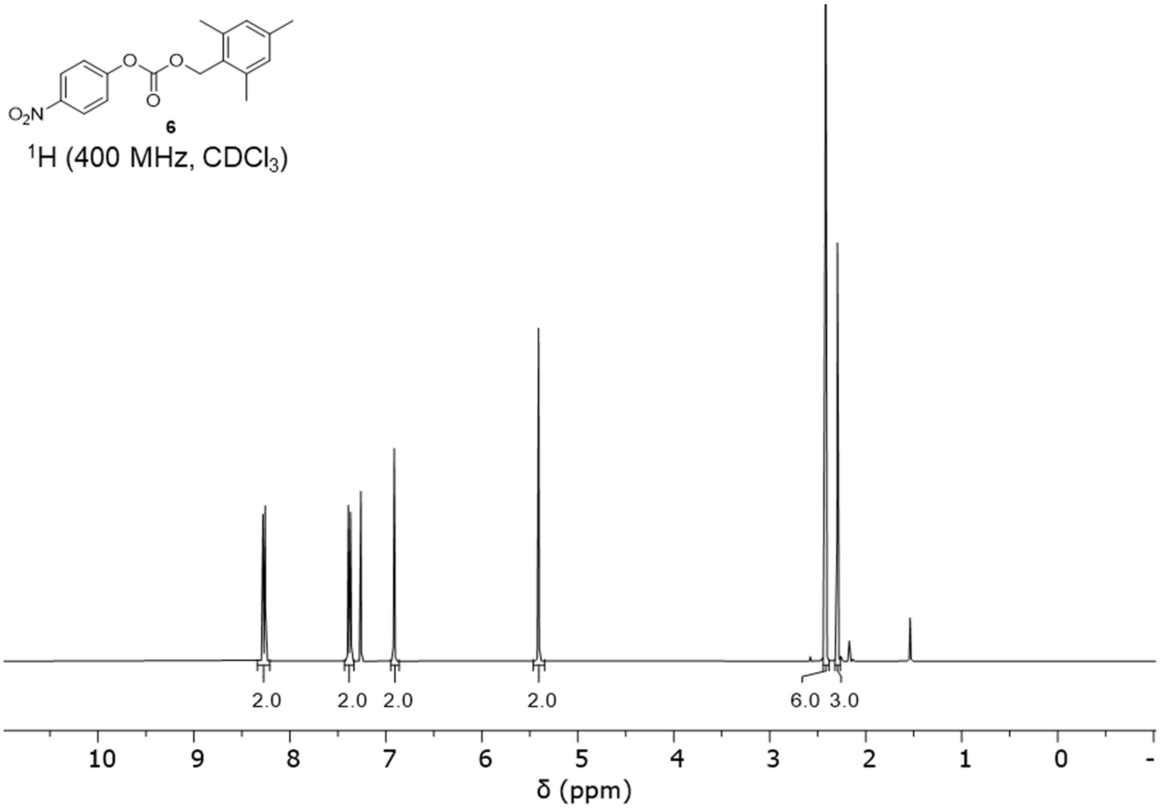

^1H (400 MHz, $CDCl_3$)

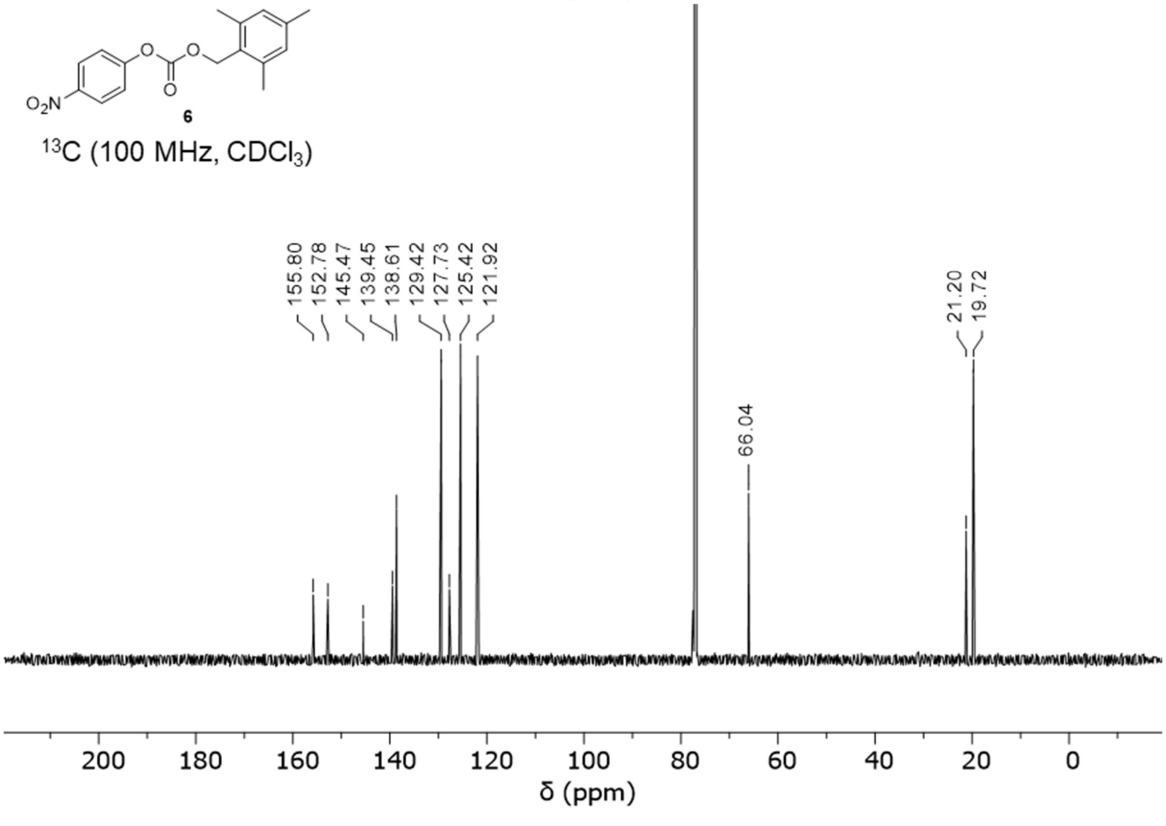

^{13}C (100 MHz, $CDCl_3$)

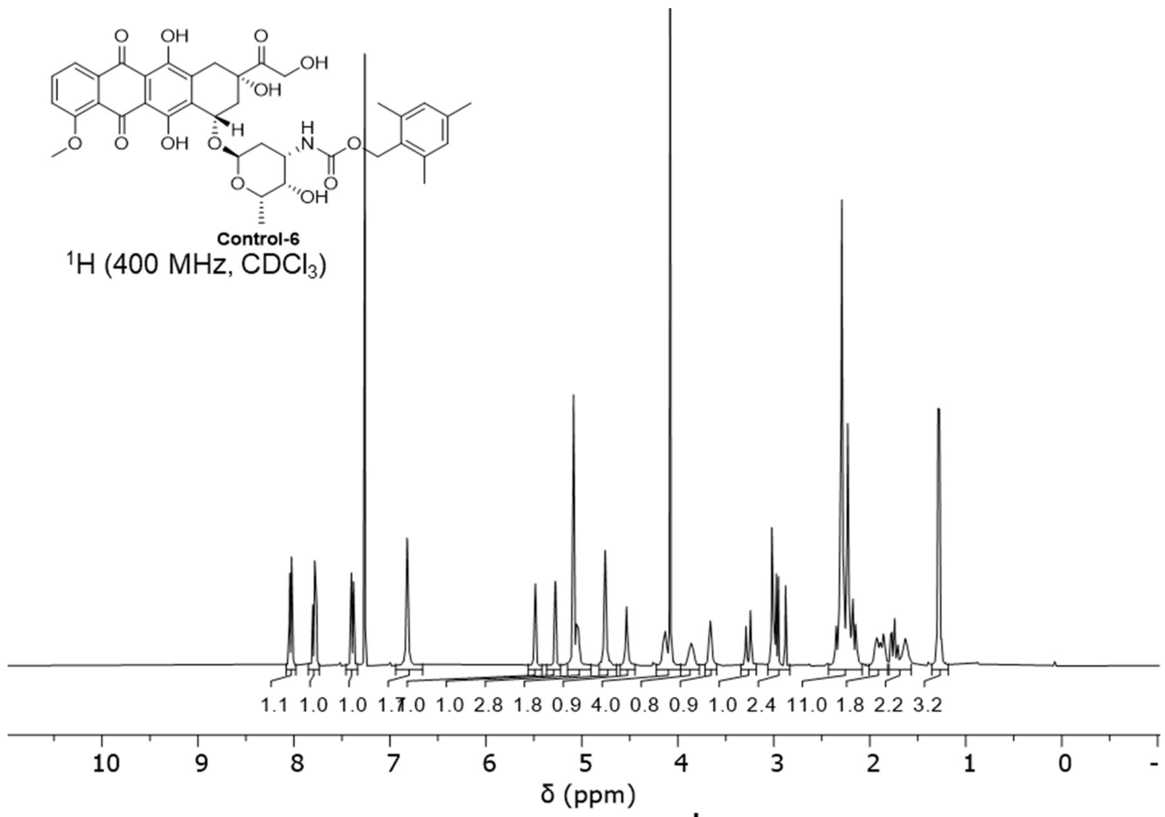
Control-6
1H (400 MHz, CDCl3)
1.1 1.0 1.0 1.7 1.0 1.0 2.8 1.8 0.9 4.0 0.8 0.9 1.0 2.4 11.0 1.8 2.2 3.2
10 9 8 7 6 5 4 3 2 1 0
δ (ppm)

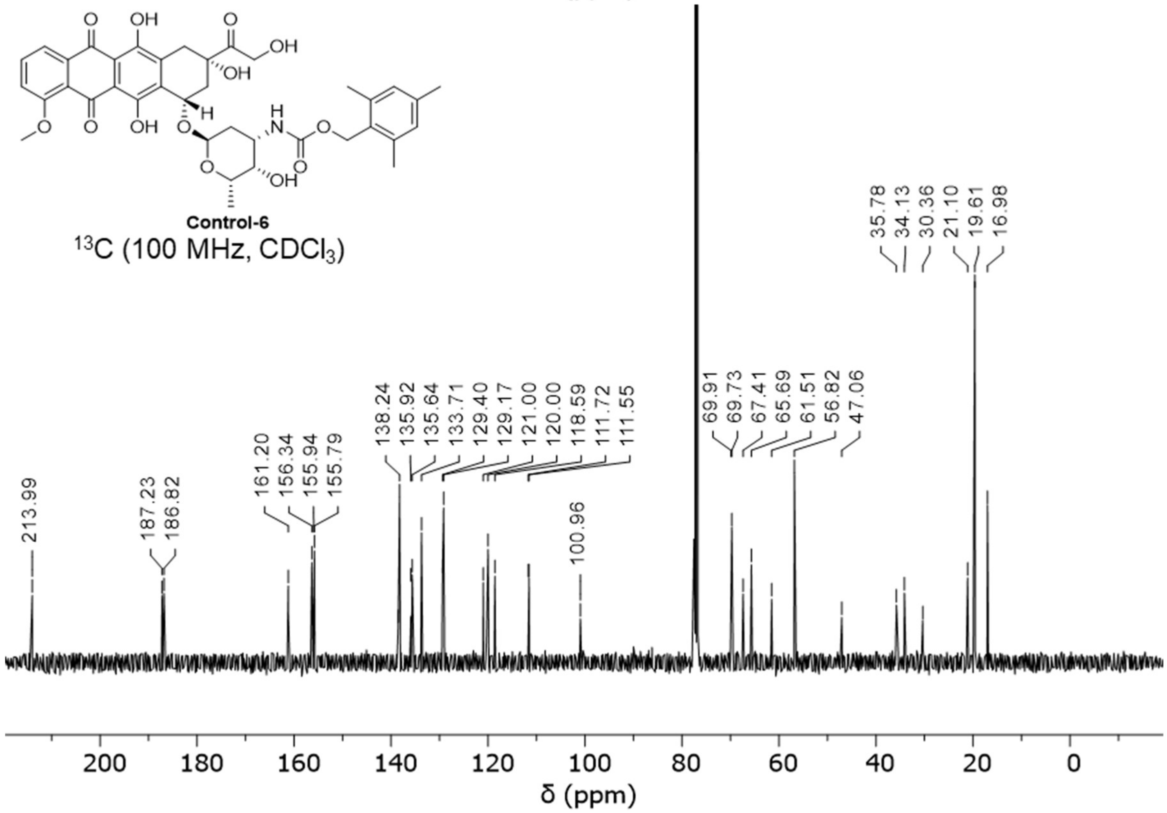
Control-6
13C (100 MHz, CDCl3)
213.99
187.23
186.82
161.20
156.34
155.94
155.79
138.24
135.92
135.64
133.71
129.40
129.17
121.00
120.00
118.59
111.72
111.55
100.96
69.91
69.73
67.41
65.69
61.51
56.82
47.06
35.78
34.13
30.36
21.10
19.61
16.98
200 180 160 140 120 100 80 60 40 20 0
δ (ppm)

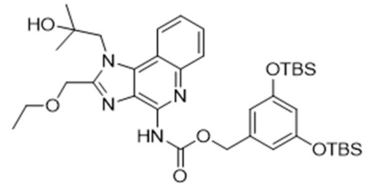

7

^{1}H (400 MHz, $CDCl_3$)

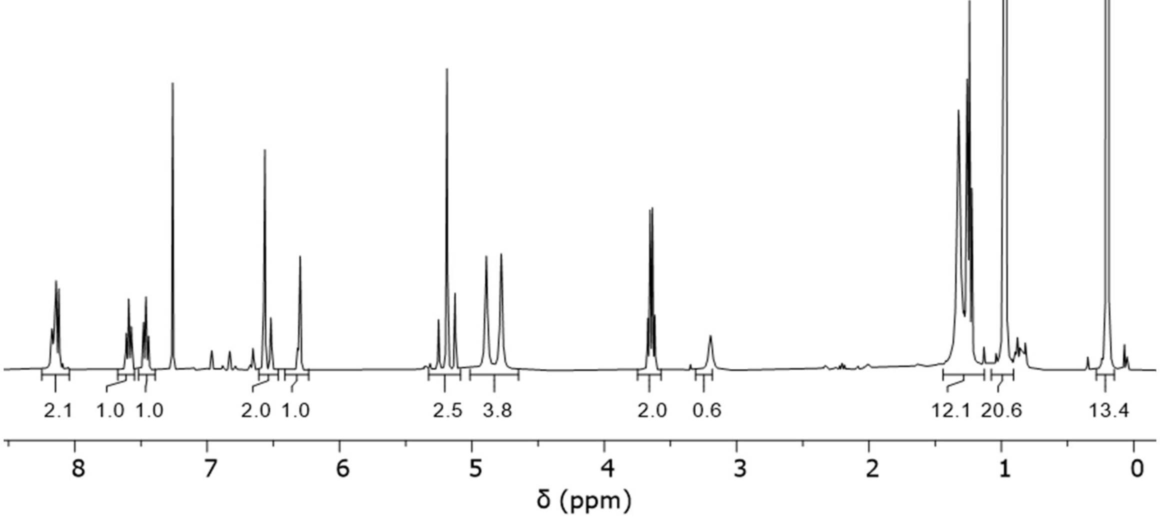

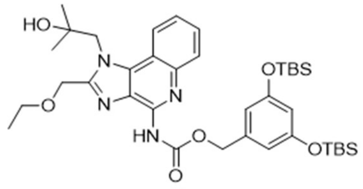

7

^{13}C (100 MHz, $CDCl_3$)

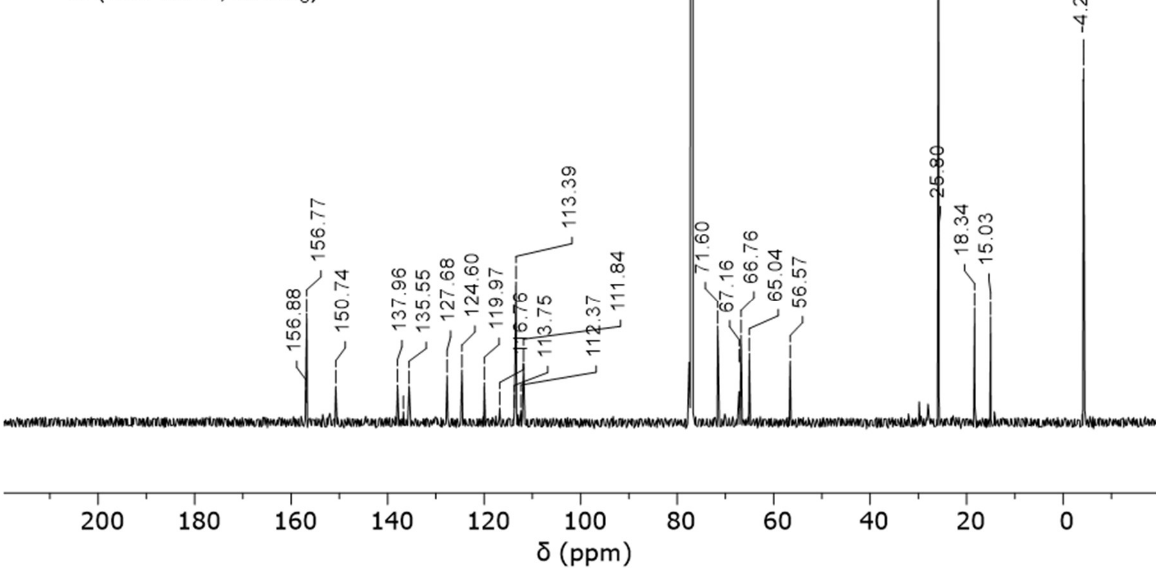

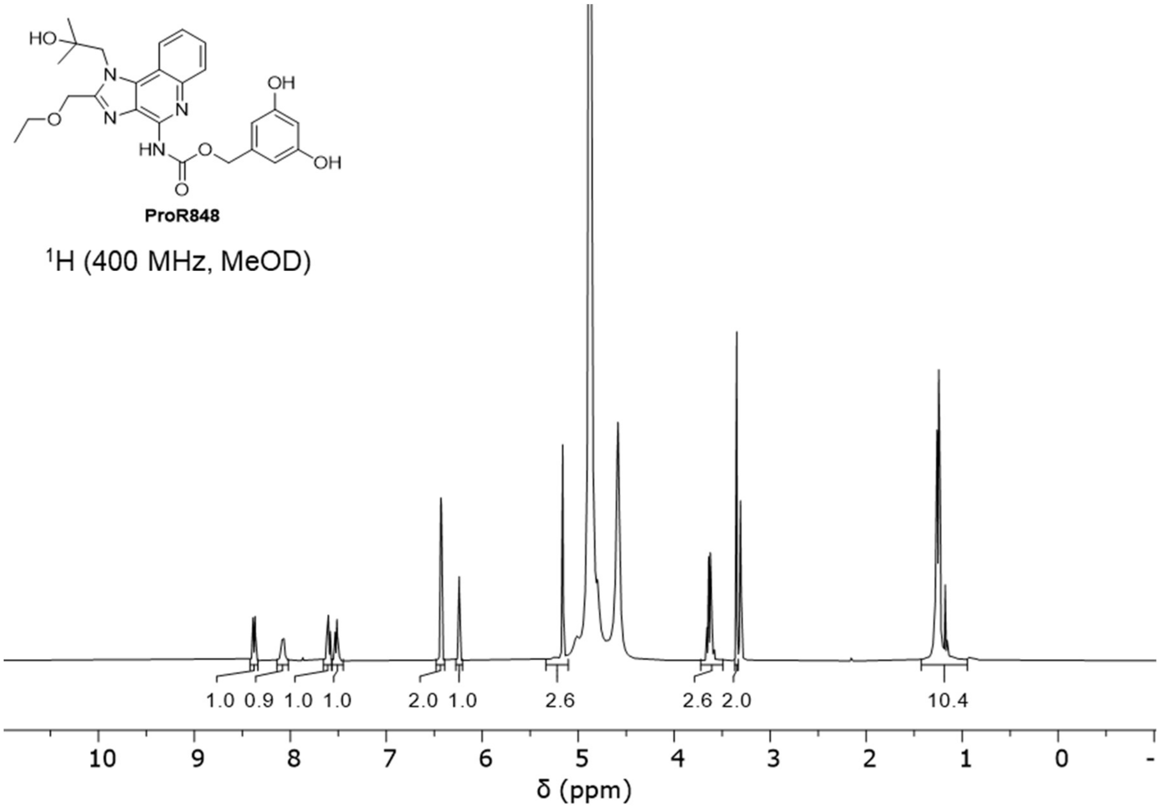
HO
N
N
N
HN
O
O
OH
OH
ProR848
1H (400 MHz, MeOD)
1.0 0.9 1.0 1.0 2.0 1.0 2.6 2.6 2.0 10.4
10 9 8 7 6 5 4 3 2 1 0
δ (ppm)

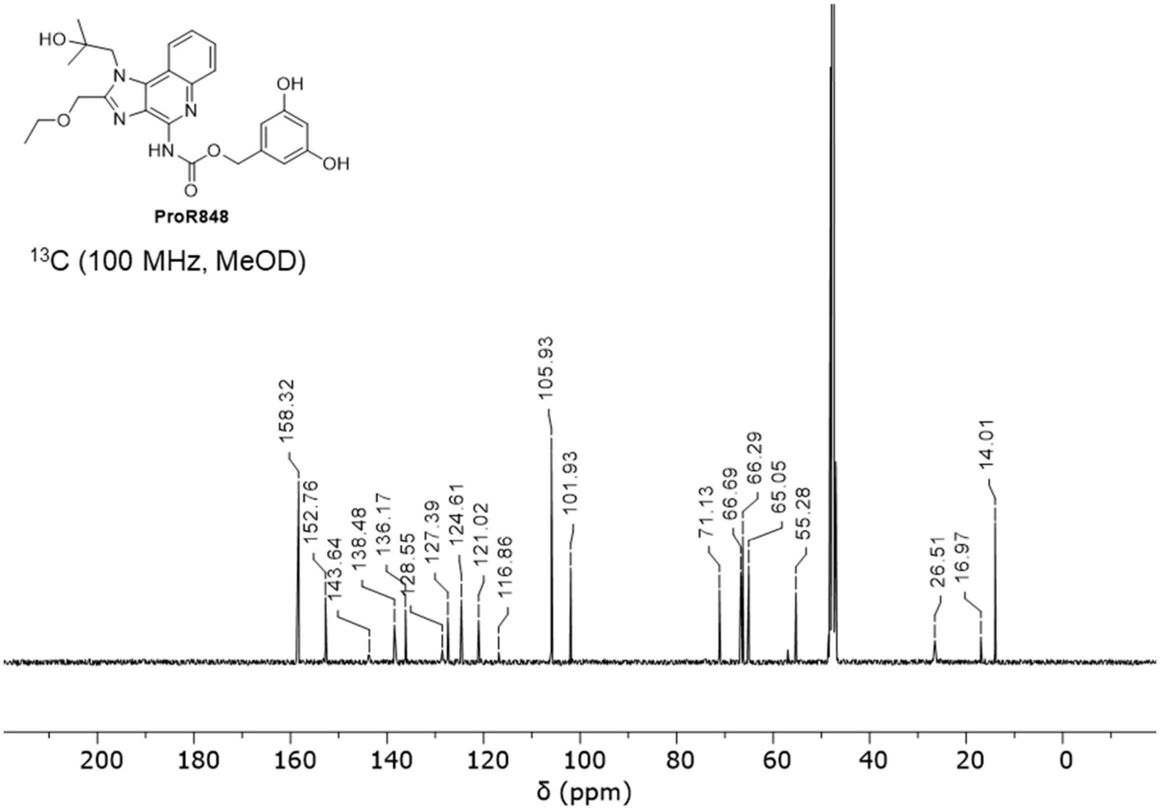
HO
N
N
N
HN
O
O
OH
OH
ProR848
13C (100 MHz, MeOD)
158.32
152.76
143.64
138.48
136.17
128.55
127.39
124.61
121.02
116.86
105.93
101.93
71.13
66.69
66.29
65.05
55.28
26.51
16.97
14.01
200 180 160 140 120 100 80 60 40 20 0
δ (ppm)
